# Strategies for improving the lives of US women aged 40 and above living with HIV/AIDS: an evidence map

**DOI:** 10.1186/s13643-018-0684-y

**Published:** 2018-02-02

**Authors:** Gaelen P. Adam, Mengyang Di, Susan Cu-Uvin, Christopher Halladay, Bryant T. Smith, Suchitra Iyer, Thomas A. Trikalinos

**Affiliations:** 1 Brown Evidence-based Practice Center, Brown University School of Public Health, Box G-S121-8, Providence, RI 02912 USA; 2 Department of Health Services, Policy & Practice, Brown University School of Public Health, Providence, USA; 3 Department of Ob-Gyn and Medicine, Warren Alpert School of Medicine, Brown University, Providence, USA; 4 Agency for Healthcare Research and Quality, U.S. Department of Health and Human Services, Rockville, MD USA

## Abstract

**Background:**

While in its early years the HIV epidemic affected primarily the male and the young, nowadays, the population living with HIV/AIDS is approximately 24% women, and its age composition has shifted towards older ages. Many of the older women who live with HIV/AIDS also live with the medical and social conditions that accompany aging. This work aims to identify and characterize empirical studies of strategies for the comprehensive management of women over 40, including transgender women, who live with HIV/AIDS. Forty was chosen as an operational age cutoff to identify premenopausal women who are less likely to bear children, as well as peri- and postmenopausal women.

**Methods:**

We conducted a literature search after discussions with a diverse panel of content experts and other stakeholders and developed an evidence map that identified 890 citations that address questions having to do with programs and barriers to engaging with programs, as well as the role of insurance and comorbidities, and have enrolled older women who live with HIV/AIDS.

**Results:**

Of these, only 37 (4%) reported results of interest for women over 40 who live with HIV/AIDS, or examined interactions between gender and older age that would allow predictions in this subgroup. Few of the 37 eligible studies focused on women facing obvious challenges, such as immigrants, transgender, physically abused, or those recently released from prison. No studies focused on women caring for dependents, including children and grandchildren, or those diagnosed after age 40.

**Conclusion:**

The evidence base that is directly applicable to women over 40 who live with HIV/AIDS in the USA is limited, and the research need is broad. We propose research prioritization strategies for this population.

**Electronic supplementary material:**

The online version of this article (10.1186/s13643-018-0684-y) contains supplementary material, which is available to authorized users.

## Background

At the end of 2014, an estimated 955,081 people were living with human immunodeficiency virus (HIV) infection in the USA. While in its early years the epidemic affected primarily males and young people [1], nowadays, the population living with HIV/AIDS is approximately 24% women and its age composition has shifted [2]. The percentage of people living with HIV/AIDS aged 50 years and older grew from 24% in 2005 to 45% in 2014. This number is projected to top 50% by 2020 [2, 3], but may have already done so [4]. People aged 40 and over comprise 72.5% of the population [2]. Thus, the management of this population who live with HIV/AIDS represents a relatively new challenge.

It is well understood that the population of older people who live with HIV (for this report, all people over the age of 40) is heterogeneous with respect to a multitude of factors that are associated with length and quality of life [2].

*Age at HIV infection*, *rather than age at diagnosis*, *may be important*, because the natural history and prognosis of HIV and acquired immunodeficiency syndrome (AIDS) among those with HIV surviving into older age and those who were diagnosed at an older age appear to be distinct [5]. Further, compared to people who were infected young, people contracting HIV at an older age are not diagnosed as promptly and tend to start treatment at a more advanced stage of the disease. Of the 428,724 people aged 50 and above living with AIDS at the end 2014, only 1.6% were diagnosed at age 50 and above; for those age 40 and above, the percentage was 1.9.

*Aging with HIV infection presents special challenges for preventing and managing comorbid conditions*. Older women with HIV/AIDS may have an increased risk for cardiovascular disease, osteoporosis, and certain cancers than their uninfected counterparts [6–9]. According to a 2013 study by the CDC, the most common conditions among women infected with HIV were viral hepatitis B or C coinfection (29.0%) or were related to cardiovascular disease or metabolic syndrome, including dyslipidemia (67.3%), hypertension (57.4%), obesity (31.7%), and low high-density lipoprotein cholesterol (27.3%). They also noted that 73.7% of women across races and ethnicities had more than one comorbidity [10]. HIV-positive antiretroviral-treated older women who achieve viral suppression are in a generalized status of immune activation and therefore may be at an increased risk of age-associated end-organ diseases compared to uninfected age-matched controls [11]. Polypharmacy is another common concern [12, 13].

*Women over 40 living with HIV/AIDS also face issues related to the interaction between reproductive hormones, HIV/AIDS, and antiretroviral treatments*. Some women may still have childbearing potential and will have unique contraceptive or fertility needs [14, 15], and there have been inconsistent reports regarding HIV and menopause [16–19].

*Older women with HIV face challenges related to mood and psychosocial wellbeing*. Compared to older women without HIV, older women with HIV infection are more likely to be depressed and lonely [20, 21], and many are burdened with taking care of elderly parents or grandchildren [22]. Stigmatization can also contribute to depression and worry and may prevent women from disclosing their HIV status or from seeking care [23].

*African American/Black women are disproportionally affected by HIV, compared to women of other race/ethnicities.* An estimated 61% of women who were living with HIV/AIDS in 2014 were African American [2]. Race is often a marker of socioeconomic and demographic factors related to health inequities, which may have a bearing on the comprehensive management of women living with HIV/AIDS. HIV rates are also highest among women living in areas where more than 21% of residents were below the federal poverty level [2].

*Special consideration should be given to women who identify as lesbian, bisexual, or transgender* (assigned male sex at birth but identify as women). For women who identify as lesbian, HIV infection may be diagnosed belatedly, and transgender women are a vulnerable subgroup for a number of medical and social reasons [24–29].

Ideally, the care of older women living with HIV/AIDS should support patient-centered approaches to managing HIV and comorbidities; incorporate patient goals, including quality of life issues; and integrate biomedical, behavioral, and social interventions [13, 30, 31]. Because people with HIV/AIDS may have an accelerated aging process and often suffer from multiple comorbidities, a geriatrics approach may be appropriate, even for those who are not chronologically geriatric [30, 31].

However, the current system of care in the USA does not support a comprehensive model that can provide a tailored approach for older women living with HIV/AIDS. This paper comprises an evidence map of the published literature on questions pertinent to older women, aged 40 and above, who live with HIV/AIDS to serve as a compendium of the evidence base (enabling further systematic study of specific questions) that can be used to identify and prioritize gaps in the evidence, without recording what the findings were or assessing risk of bias and the strength of the evidence (which was infeasible given the scope and heterogeneity of interventions and outcomes).

## Methods

The protocol for this project was not registered with PROSPERO, but was posted prospectively on the Agency for Healthcare Research and Quality (AHRQ) Web site (https://effectivehealthcare.ahrq.gov/topics/women-hiv/research-protocol/).

### Key informants

We assembled a panel of eight Key Informants that included experts in the care of HIV patients, nationally recognized researchers, policy makers, state government (Department of Health) officials, and nationally recognized advocates for women who live with HIV/AIDS to share their perspective on the issues that the guiding questions address. Based on these discussions, we refined study eligibility criteria and data extraction items for the evidence map of empirical studies that apply to women over 40 who live with HIV/AIDS. We grouped the studies into three areas of interest: (Area 1) studies that measure the impact of or describe barriers to engaging with existing resources; (Area 2) studies that measure the impact of insurance coverage on outcomes related to engaging with a program or accessing care; and (Area 3) studies that assess the performance of diagnostics for co-occurring disease or assess the effects of treating co-occurring diseases or risk factors.

### Eligibility criteria

Eligible studies reported results stratified by age and included at least 75% of the participants were women 40 years and older who live with HIV, an operational age cutoff to identify premenopausal women who are less likely to bear children, and peri- and postmenopausal women. All patient-level and system-level outcomes were eligible. Eligible services provided medical, behavioral, or social support or were programs that bundled several such services.

All study designs were eligible, including qualitative studies, which informed on barriers to accessing care. We excluded studies that analyzed fewer than 10 women who are older than 40 and live with HIV/AIDS, because they were unlikely to yield precise or broadly applicable conclusions. We also excluded studies completed longer than 10 years ago, because older empirical data are less likely to be relevant to today’s setting. Because the main focus of the evidence map is to inform about the US setting, we did not include studies conducted exclusively in other countries. Finally, we excluded studies not reporting empirical data.

### Literature identification and data abstraction

A medical librarian designed and implemented searches in PubMed, PsycINFO, CINAHL, and SocINDEX for terms related to HIV or AIDS, crossed with terms on interventions, policies, services, or programs. We limited the search to English language reports published after January 01, 2005, having at least one author with a US affiliation. The search was last run on February 10, 2016, and is reported in full in Additional file 1: Appendix 1 in the full report of the project [32]. After a pilot phase to ensure that the eligibility criteria was being correctly and uniformly applied, abstracts were single-screened by a human reviewer and double-checked against the predictions of the machine learning algorithms implemented in *Abstrackr* [33]. All potentially eligible citations were retrieved and screened in full text for eligibility. For these papers, we recorded reasons for exclusion. For papers on studies that were excluded only because they did not report subgroup-specific information or pertinent interaction analyses, we recorded the area of interest the paper fell into.

All data were extracted in a predefined electronic form by a single investigator. The form recorded bibliographic information; demographic details for women over 40 where available, and for all women in the study where not; along with details about the programs, barriers, insurance impact, and comorbidities. Due to the nature of the project, we did not extract results, nor did we make strength of evidence assessments for individual studies or for the whole evidence base.

## Results

**Fig. 1:**
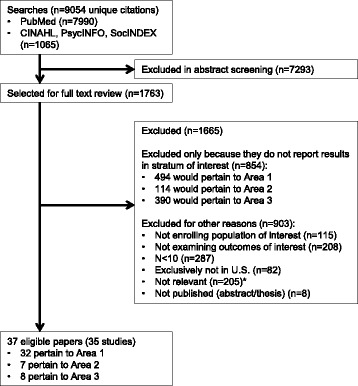
PRISMA literature flowchart [61]. * “Not relevant”: these studies were excluded on the grounds of more than one reason (e.g., did not describe eligible outcomes of eligible programs in an eligible population)

We screened 9054 unique abstracts for eligibility, of which 1763 were selected for full text review. In the end, 32, 7, and 8 papers were eligible for the three areas of interest, respectively (Fig. 1). Of the citations that were excluded in full text review, 854 were excluded because they did not provide information on strata of women who live with HIV/AIDS and are at least 40 years old or did not analyze interactions between gender and older age. Details on these studies are available in Additional file 1: Appendix B.

### Area 1: studies measuring the impact of strategies for engaging resources or describing barriers to accessing resources

Thirty-two papers were eligible in Area 1; 16 quantified the impact of strategies for engaging resources, and 18 were about barriers to accessing resources (one paper was about both). Additional file 1: Appendix B, Table S1 summarizes the characteristics of the women enrolled in these studies. Most studies were conducted in urban centers, though a few focused on rural populations or examined differences between rural and urban populations. Studies included an average of 70 women (nine included more than 200 women). Mean ages ranged between 36 and 57 years, and no study provided results in the elderly. No study reported the women’s menopausal status or the age at which they were diagnosed with HIV.

Among the 28 studies that reported information on the race/ethnicity of participants, the median proportion of African American, Latino/Hispanic, and White women was 78, 15, and 14%, respectively. Three studies enrolled a small proportion of Native American women, and one included Pacific Islander women. No study enrolled Asian women.

On average, 20% of women reported having a spouse or partner, 58% perceived having adequate social support, and 31% cared for a dependent, but such information was provided in less than a third of the studies. Among studies listing such information, the average proportion of women who had annual income less than approximately $10,000 were unemployed, had not finished high school, used illegal substances, or faced mental health challenges ranged between 31 and 75%. Only one or two studies reported on the proportion of women who were undocumented immigrants (one study), experienced violence (two studies), or had a history of incarceration (one study).

Cumulative data from seven studies reported that a median of 73% of women had some form of public insurance (Medicare or Medicaid), 15% had private insurance, and 27% had no insurance. However, this may have changed or be changing as the Affordable Care Act is implemented.

Finally, the vast majority of studies did not explicitly report information on sex of women’s sexual partners or gender self-identification. No study reported the proportion of women who have more than one concurrent sexual partner, four studies included women who were identified in the study as lesbian or bisexual (ranging from 6.6 to 33% of the study population), and one study enrolled only transgender women.

### Studies measuring the impact of strategies for engaging resources

Sixteen papers on 13 unique studies measured the impact of engaging with various services or with programs bundling several services. Eight RCTs (described in 10 papers) randomized between 30 and 184 participants to receive a specific program or an alternative (no program or a different program). An additional six studies qualitatively assessed the impact of engaging with programs. Three were based on interviews of 15 to 21 participants [34–36], a fourth examined a cohort of 364 participants in a stress-management intervention [37], and two examined programs aimed at reducing substance use in 46 and 76 participants, respectively [38, 39]. Details of all programs and measured outcomes are listed in Table 1.

**Table 1:** Characteristics of studies of strategies to promote women’s engagement with resources

Author (year), state	Mean age	Intervention or comparison	Intervention objective	Medical component	Psychosocial component	Case management component	Health education component	STD prevention component	Outcomes measured
Randomized controlled trials									
Abel (2006), Texas [62]	44	Writing intervention	Reducing perceived HIV stigma	No	Yes	No	No	No	Perceived HIV stigma
Chander (2015), Maryland [49]	44	Brief alcohol intervention	Reducing drinking; reducing risky sexual behavior	No	Yes	No	Yes	Yes	Alcohol consumption, sexual behavior, HIV outcomes
Echenique (2013), Florida [63]	NS	Project ROADMAP, Reeducating Older Adults in Maintaining AIDS Prevention	Reducing risky sexual behavior	No	Yes	No	Yes	Yes	Sexual risk behavior/HIV knowledge
Feaster (2010), Florida [64]^a^	36	Structural Ecosystems Therapy vs usual care	Promote healthy family and social relationships	No	Yes	No	Yes	No	Self-reported medication adherence
Feaster (2010), Florida [46]^a^	43	Structural Ecosystems Therapy vs usual care	Address relapse prevention and medication adherence	No	Yes	No	Yes	No	Self-reported substance abuse, medication adherence
Mitrani (2012), Florida [47]^a^	43	Structural Ecosystems Therapy vs usual care	Address relapse prevention and medication adherence	No	Yes	No	Yes	No	Psychological Distress and Drug Abstinence (Brief Symptom Inventory, self-reported illicit drug use)
Teti (2010), Pennsylvania [65]	40	Protect and Respect vs educational information	Decrease risky sexual practices	No	Yes	No	Yes	Yes	Self-reported disclosure of HIV status to partners; condom use
El-Bassel (2011), Georgia, California, New York, Pennsylvania [66]	NS	Risk reduction intervention vs health promotion intervention	Influence behaviors linked to chronic disease, including diet and exercise	Yes	No	No	Yes	Yes	Mammography screening
DeMarco (2013), Massachusetts [67]	NS	Sistah Powah Structured Writing Intervention vs attention control	Increased use of cognitive behavioral self-help programs, regular medical and HIV care, psychosocial support	No	Yes	No	No	No	Adherence to health care: keeping appointments, risky sexual behavior, testing for comorbidities, needle care, lifestyle factors
Manuel (2013), California [68]	49	Motivational Interviewing vs prescribed advice	Smoking cessation	Yes	Yes	No	Yes	No	Smoking intensity and cessation
Observational studies									
Cocohoba (2013), California [35]	NS	Pharmacy intervention	Promote ART adherence	Yes	No	Yes	Yes	No	ART adherence
Dutcher (2011), USA [34]	NS	Peer support	Social support	No	Yes	No	Yes	Yes	Care adherence/housing status
Kupprat (2009), New York [38]	47	Social support substance use and mental health services	Substance abuse treatment, mental health services, case manager, support groups	Yes	Yes	Yes	No	No	Attendance, reception of therapy
Proeschold-Bell (2016), North Carolina [39]	46.5	Substance Use Treatment Integrated Care from Social Workers and HIV Medical Providers	Substance use treatment	Yes	Yes	No	Yes	No	Alcohol/drug use
Sullivan (2015), North Carolina [36]	45	Guide to Healing Program	Link HIV infected people to care	No	No	Yes	Yes	No	Self-reported ART adherence and medication management; accessing resources, including medication assistance, and community based services
Weiss (2015), Florida, New York, New Jersey [37]	45	SMART/EST Women’s Program	Enhance quality of life	No	Yes	No	No	No	Depression, medication adherence

*SMART/EST* Stress Management And Relaxation Training/Emotional Supportive Therapy

^a^These papers refer to the same study

### Studies of barriers to accessing resources

Eighteen observational studies evaluated barriers to care in populations ranging from 17 to 1701 women. Almost all of the barriers studied involved engaging or retention in HIV care, though three studies exclusively assessed outcomes not related to HIV, including cancer screening (one study), accessing program services online (one study), and using the internet (one study) (Table 2). Of the 14 studies that evaluated barriers to accessing care or retention in care, the barriers were sociodemographic in 12, cultural in 6, psychosocial in 11, having to do with experience with incarceration in 1, having to do with medical history in 12, and having to do with mental history in 6. Two studies reported on barriers to cancer screening that were sociodemographic, psychosocial, and medical in nature [40, 41], and a third evaluated barriers to adherence to treatment in the context of a pharmacy program that were psychosocial and medical [35]. Two studies, both by Blackstock et al., evaluated barriers having to do with using the Internet, either in general or to access social support. These studies identified barriers from nearly every aspect (sociodemographic, cultural, psychosocial, medical history, and mental history) [42, 43], which may inform the design of Web-based programs, as well as strategies to enhance their uptake and use.

**Table 2:** Characteristics of studies of barriers to accessing resources

Author (year)	Mean age	Study design (data collection method)	Barriers to...	Person-level modifiers examined						System-level (including caregiver-related) modifiers examined
				Sociodemographic	Cultural	Psychosocial	Exp. with incarceration	Medical history	Mental history	
Studies of barriers to accessing or remaining in care										
Burke-Miller (2006), Multiple^a^ [69]	NS	Observational (interviews and examinations)	Engaging in care	Yes	Yes	Yes	No	Yes	Yes	None
Blackstock (2015), Multiple^b^ [53]	42	Observational (interviews)	Engaging in care	Yes	No	Yes	No	Yes	Yes	Transportation
Williams (2013), Multiple^c^ [55]	41	Observational (survey)	Engaging in care	Yes	No	Yes	Yes	Yes	Yes	Intensity of care services received in jail
Tello (2010), Maryland [41]	46	Observational (survey + focus groups)	Engaging in care; cancer screening	Yes	No	Yes	No	Yes	No	Transportation, relationship with provider
Toth (2013), North Carolina [70]	46	Observational (interviews)	Engaging in care	Yes	Yes	Yes	No	Yes	Yes	Transportation, financial, other logistical
Sevelius (2014), California [54]	NS	Observational (interviews + focus groups)	Engaging in care	Yes	Yes	Yes	No	Yes	No	Provider/staff cultural competence, integrated transgender and /HIV care, confidentiality
Stevens (2009), Wisconsin [71]	41	Observational (interviews)	Engaging in care	No	No	No	No	No	No	Insurance, transportation, financial, provider turnover
Fletcher (2014), Texas [40]	51	Observational (focus groups)	Cervical cancer screening	No	No	Yes	No	Yes	No	Transportation, wait times, scheduling
Quinlivan (2013), North Carolina [72]	45	Observational (interviews)	Engaging in care	Yes	Yes	Yes	No	Yes	Yes	Navigating labs, transportation and parking, relationship with providers
Vyava-harkar (2008), South Carolina [73]	44	Observational (focus groups)	Engaging in care	Yes	Yes	Yes	No	Yes	No	Relationship with provider
Pivnick (2010), New York [52]	48	Observational (interviews + focus groups)	Engaging in care	Yes	Yes	Yes	No	Yes	No	None
McDoom (2015), Massachusetts [74]	57	Observational (interviews)	Engaging in care	No	No	Yes	No	No	No	Relationship with provider
Kempf (2010), Alabama [75]	46	Observational (focus groups)	Retention in care	Yes	No	Yes	No	Yes	No	Transportation, clinic hours, flexible scheduling
Kupprat 2009, New York [38]	47	Observational (chart review)	Engaging in care	Yes	No	No	No	Yes	Yes	Unclear
Sarnquist 2011, California [76]	NS	Observational (interviews)	Engaging in care	Yes	No	No	No	Yes	No	Transportation, navigating healthcare system
Studies of barriers to other goals										
Blackstock (2015), New York [42]	50	Observational (interviews)	Using the Web	Yes	Yes	Yes	No	Yes	Yes	Place of medical care (clinic, private PCP vs healthcare for homeless, methadone clinic, visiting PCP)
Blackstock (2015), New York [43]	49	Observational (interviews)	Accessing Web-based social support	Yes	Yes	Yes	No	Yes	No	None
Cocohoba (2013), California [35]	NS	Observational (interviews)	Adhering to ART	No	No	Yes	No	Yes	No	Privacy, pharmacy location, presence of drug-seeking or intoxicated pharmacy patrons, relationship with provider

*ART* antiretroviral therapy, *NS* not stated

^a^NYC (NY), Washington (DC), Chicago (IL), LA and San Francisco (CA)

^b^Brooklyn (NY), Chicago (IL), LA (CA), Miami (FL), San Antonio (TX), Springfield (MA), Alabama (AL), (NC), Longview (TX)

^c^Atlanta (GA), Chester (PA), Chicago (IL), Cleveland (OH), Columbia (SC), New Haven (CT), New York city (NY), Philadelphia (PA), Providence (RI), Springfield (MA)

The most commonly cited system- or provider-level factors were lack of transportation and logistical challenges, including navigating, wait times, clinic hours, insurance-related bureaucracy (six studies), and relationship with the provider (four studies).

### Area 2: studies measuring the effect of insurance

Seven eligible papers evaluated the overall effect of insurance coverage levels on outcomes related to engaging with a program or accessing care. Six of these are included among the studies of barriers described in the previous section. Full details for the population are in Additional file 1: Appendix B, Table S2. Of these, six studies reported the impact of different insurance coverage on engagement with or retention in care, satisfaction with care, antiretroviral adherence, and Internet use. The seventh study evaluated the association between insurance coverage and successful management of major depressive disorder [44].

In no study did more than 18% of the women have private insurance. Where it was reported, between 60 and 100% of women had public insurance and between 7 and 35% had no insurance. In all studies, insurance coverage was one of several factors in a regression model for engaging with or accessing care. Although we did not perform risk of bias assessments, we deem it unlikely that such analyses can yield good estimates of the causal effect of insurance on outcomes. These studies were not designed to estimate the causal effect of insurance, and each study considered and controlled for different variables.

### Area 3: studies on the diagnosis or management of comorbidity, or on predictive models

Eight papers (seven studies), with sample sizes between 46 and 1234 participants, were deemed eligible. Full details for the population are in Additional file 1: Appendix B, Table S3. No study informed on diagnosis or screening for comorbidities among older women living with HIV/AIDS; six reported information on the management of comorbidities; and two reported on predictive models of HIV progression or overall mortality in the presence of comorbid conditions.

Of the six studies that included information on management of comorbidities, two pertained to depression, three to alcohol and other substance abuse, and one to cardiovascular disease. Of the two studies on depression, one examined whether psychopharmacologic and psychotherapeutic treatment of depressed HIV-women met standards defined in the best practice literature and assessed predictors of standard-concordant care [44], and the other examined whether antidepressant use by HIV-positive patients results in better employment rates [45]. Among the three studies of drug and alcohol abuse, one evaluated a family-based intervention (Structural Ecosystems Therapy) compared to a psychoeducational intervention [46, 47]. A second looked at associations between heavy alcohol consumption and antiretroviral treatment initiation and mortality [48], and a third evaluated a brief intervention to reduce or eliminate alcohol consumption in seropositive women with heavy drinking problems [49]. Finally, a cohort study reported rates of acute myocardial infarction among HIV-positive patients with cardiovascular risk factors [50].

Two eligible cohort studies described models predicting HIV progression or overall mortality among older women living with HIV/AIDS who also have comorbidities. The first predicted clinical progression of HIV disease based on illicit drug use and various sociodemographic factors (such as age, race, and sexual history) and medical history factors (such as antiretroviral use, CD4 cell counts, and CDC category of HIV disease at baseline) [51]. The second predicted all-cause mortality among older women living with HIV/AIDS who also have substance and alcohol abuse problems based on sociodemographic factors (age and race), medical history factors (including using various drugs, alcohol and tobacco, having hepatitis C, prior antiretroviral therapy, CD4 counts, and viral load), and mental health factors (including a history of depression, anxiety, or psychosis) [48].

## Discussion

We identified 890 citations that address questions in the three areas of interest and have enrolled women age 40 and over, who live with HIV/AIDS. Of these, only 37 (4%) studies reported results of interest among older women who live with HIV/AIDS or examined interactions between gender and older age that would allow extrapolation to this subgroup. An additional 494, 114, and 390 papers would have been eligible for the three areas of interest, had they reported the required information on women age 40 and over. This observation is not surprising, because the page restrictions on journal articles limit the examination of the many subgroups of interest. It is, however, congruent with the prediction of the Key Informants that evidence for age grouping would be sparse.

While some of the 37 eligible studies focused on populations facing obvious challenges, such as immigrant women [52], women who had suffered physical abuse [53], transgender women [54], and women recently released from prison [55], more studies are needed in these subgroups. No study focused on women caring for dependents, those diagnosed in old age, or those who face less obvious challenges. For example, no published data have assessed the safety and efficacy of hormone therapy on symptoms of menopause, cardiovascular risk, and bone disease among women living with HIV/AIDS [56]. The pharmacological interactions between hormone therapy and antiretroviral treatments are not well understood, with some studies reporting menopause at an earlier age among women living with HIV/AIDS compared to uninfected women [16–18, 56–59], and other studies suggesting that women with HIV have more vasomotor symptoms (e.g., hot flashes, night sweats) and mood issues during menopause [56].

We identified eight generally small randomized trials that examined the impact of strategies for promoting healthy family and social relationships and healthier lifestyle. Most included psychosocial and health education components, but none included a comprehensive case-management-based approach. A handful of nonrandomized studies also evaluated the effectiveness of strategies for promoting care. We identified 18 studies of barriers to accessing care, which most commonly examined barriers related to sociodemographic, psychosocial, and medical factors. A minority examined barriers related to cultural factors, experience with incarceration, or mental health. Several studies identified system-level barriers to engaging with care, including transportation, scheduling, insurance referrals, financial issues, and practical challenges, such as navigating a hospital building. Finally, very few studies examined the overall impact of insurance on outcomes, or the impact of comorbidity on the management of HIV infection or its progression. Thus, although we did not analyze the findings of the aforementioned studies in all clinical areas, it is highly unlikely that the identified body of evidence is close to maturity.

### Limitations

This work has several limitations. We developed an evidence map to describe the amount and type of practically available evidence, but did not summarize findings of eligible studies. For feasibility, we did not consider research completed longer than 10 years ago, because older empirical data are less likely to be relevant to today’s setting and for informing the future research agenda. We decided not to search EMBASE or any other large, international general database, because we deemed that the added benefit of searching multiple targeted databases was more cost and time efficient and would yield more relevant studies than a second large general database search [60].

We did not conduct a detailed analysis of the 37 eligible papers, prioritize outcomes by their importance, or do a detailed analysis of the risk of bias or the strength of evidence of individual studies or of the entire evidence base, because these tasks were not relevant to the goal of this project, which was to describe the literature in broad terms.

Because the main focus of the evidence map was to identify studies that inform about the US setting, we did not consider studies conducted exclusively in other countries. To operationalize this, we limited our search to papers in which at least one author had a US affiliation. In screening, we excluded a study only if it reported that it had been done exclusively outside the USA.

Because of limited time and resources, screening and data extraction were done by a single experienced reviewer. We believe that the overall picture of the research is accurate.

### Research needs

The 37 eligible studies do not represent a mature evidence base. The evidence base is even more sparse if one considers that the identified studies focused on different research questions and cannot possibly cover the range of practically implementable interventions that should be examined. By their nature such interventions are complex, in that they have many versions, and their implementation and deployment can depend on the targeted population.

More generally, when the range of questions of interest is so large, and the directly applicable evidence base is so limited, most, if not all, questions can be characterized as under-researched. The following are some general observations, which may help those looking to prioritize this research:

1. There is a need for better quality demographic information for women over the age of 40 living with HIV/AIDS in the USA, to allow better understanding of their care needs.
2. A large amount of data have already been collected on women who are over 40 years and live with HIV in the context of studies that enrolled broader populations, but have not been analyzed for this subgroup. Substantial economy of resources can be had by encouraging re-analyses of existing datasets, with a focus on older women who live with HIV/AIDS.
3. It is also possible that inferences about women who live with HIV/AIDS in the USA and are older than 40 years can be generalized from studies that included broader populations. A plausible approach would be to analyze existing large studies to see whether and how inferences would change in subgroups defined by age or other factors. Other sources of this sort of data include claims data, the CDC Medical Monitoring Project, and large cohort studies, such as Women’s interagency HIV study (WIHS), the NA Accord, the SUN study, and Ryan White data (HRSA).
4. Observing the great successes of clinical research consortia that were formed to address medical questions, such as the AIDS Clinical Trials Group network, and the Women’s Interagency Health Study, we believe that encouraging analogous consortia for addressing questions related to health services research, barriers, social interventions, economics, and other aspects of living and aging with HIV might be fruitful.

## Conclusions

The population of women over 40 living with HIV/AIDS is both growing and under-researched. More research is needed to learn about the challenges faced by these women and how they differ across demographics. It is important to identify the needs of women over 40 living with HIV/AIDS and engage them in the prioritization, design, and interpretation of research. This research should be aimed at facilitating a comprehensive model of care that can provide a tailored approach for older women living with HIV/AIDS across the continuum of care.

## Additional file

Additional file 1: Appendix A. Complete search strategies and Appendix B. Characteristics of women in included studies. (DOCX 428 kb)

## Abbreviations

AHRQ: Agency for Healthcare Research and Quality
AIDS: Acquired immunodeficiency syndrome
HIV: Human immunodeficiency virus
