# Supplementary material for: Strategies for improving the lives of US women aged 40 and above living with HIV/AIDS: an evidence map
Source: Syst Rev. 2018 Feb 2;7:25. doi: 10.1186/s13643-018-0684-y (PMC5796491; doi:10.1186/s13643-018-0684-y)
Supplement: Additional file 1: — Appendix A. Complete search strategies and Appendix B. Characteristics of women in included studies. (DOCX 428 kb) [file 13643_2018_684_MOESM1_ESM.docx]

Additional file 1

Appendix A. Search Strategy

PubMed Programs search 11/30/2015, updated 2/10/2016

((older or “middle age” or “middle aged” or “middle-age” or “middle-aged” or "Middle Aged"[Mesh] OR "Aged"[Mesh] or elderly or post-menopause or postmenopause or "Postmenopause"[Mesh])

AND (HIV or "HIV"[Mesh] or “Human immunodeficiency virus” or "HIV Infections"[Mesh] or (“Acquired Immunodeficiency Syndrome”) or "Acquired Immunodeficiency Syndrome"[Mesh])

AND (USA[Affiliation] or US or “United States” OR Alabama[Affiliation] OR AL[Affiliation] OR Alaska[Affiliation] OR AK[Affiliation] OR Arizona[Affiliation] OR AZ[Affiliation] OR Arkansas[Affiliation] OR AR[Affiliation] OR California[Affiliation] OR CA[Affiliation] OR Colorado[Affiliation] OR CO[Affiliation] OR Connecticut[Affiliation] OR CT[Affiliation] OR Delaware[Affiliation] OR “DE”[Affiliation] OR Florida[Affiliation] OR FL[Affiliation] OR Georgia[Affiliation] OR GA[Affiliation] OR Hawaii[Affiliation] OR HI[Affiliation] OR Idaho[Affiliation] OR ID[Affiliation] OR Illinois[Affiliation] OR IL[Affiliation] OR Indiana[Affiliation] OR Iowa[Affiliation] OR IA[Affiliation] OR Kansas[Affiliation] OR KS[Affiliation] OR Kentucky[Affiliation] OR KY[Affiliation] OR Louisiana[Affiliation] OR LA[Affiliation] OR Maine[Affiliation] OR ME[Affiliation] OR Maryland[Affiliation] OR MD[Affiliation] OR Massachusetts[Affiliation] OR MA[Affiliation] OR Michigan[Affiliation] OR MI[Affiliation] OR Minnesota[Affiliation] OR MN[Affiliation] OR Mississippi[Affiliation] OR MS[Affiliation] OR Missouri[Affiliation] OR MO[Affiliation] OR Montana[Affiliation] OR MT[Affiliation] OR Nebraska[Affiliation] OR NE[Affiliation] OR Nevada[Affiliation] OR NV[Affiliation] OR New Hampshire[Affiliation] OR NH[Affiliation] OR New Jersey[Affiliation] OR NJ[Affiliation] OR New Mexico[Affiliation] OR NM[Affiliation] OR New York[Affiliation] OR NY[Affiliation] OR North Carolina[Affiliation] OR NC[Affiliation] OR North Dakota[Affiliation] OR ND[Affiliation] OR Ohio[Affiliation] OR OH[Affiliation] OR Oklahoma[Affiliation] OR OK[Affiliation] OR Oregon[Affiliation] OR Pennsylvania[Affiliation] OR PA[Affiliation] OR Rhode Island[Affiliation] OR RI[Affiliation] OR South Carolina[Affiliation] OR SC[Affiliation] OR South Dakota[Affiliation] OR SD[Affiliation] OR Tennessee[Affiliation] OR TN[Affiliation] OR Texas[Affiliation] OR TX[Affiliation] OR Utah[Affiliation] OR UT Vermont[Affiliation] OR VT[Affiliation] OR Virginia[Affiliation] OR VA[Affiliation] OR Washington[Affiliation] OR WA[Affiliation] OR West Virginia[Affiliation] OR WV[Affiliation] OR Wisconsin[Affiliation] OR WI[Affiliation] OR Wyoming[Affiliation] OR WY[Affiliation] OR Puerto Rico[Affiliation] OR PR[Affiliation])

AND (women or female or "Women"[Mesh])

AND (program* OR treatment* OR intervention* or "Policy"[Mesh] or polic* or "Insurance"[Mesh] or insurance or "Government Agencies"[Mesh] or non-profit or “non profit” or nonprofit or "Organizations, Nonprofit"[Mesh] or “cascade of care”))

NOT ("Africa"[Mesh] OR (“addresses”[pt] or “autobiography”[pt] or “bibliography”[pt] or “biography”[pt] or “case reports”[pt] or “comment”[pt] or “congresses”[pt] or “dictionary”[pt] or “directory”[pt] or “editorial”[pt] or “festschrift”[pt] or “government publications”[pt] or “historical article”[pt] or “interview”[pt] or “lectures”[pt] or “legal cases”[pt] or “legislation”[pt] or “letter”[pt] or “news”[pt] or “newspaper article”[pt] or “patient education handout”[pt] or “periodical index”[pt] or "comment on" or review[tw] OR ("Animals"[Mesh] NOT "Humans"[Mesh]) OR rats[tw] or cow[tw] or cows[tw] or chicken*[tw] or horse[tw] or horses[tw] or mice[tw] or mouse[tw] or bovine[tw] or sheep or ovine or murinae))

Limit to Female

Limit to 2005-

Limit to humans

Limit to English

PubMed Comorbidities search 11/30/2016

((older or “middle age” or “middle aged” or “middle-age” or “middle-aged” or "Middle Aged"[Mesh] OR "Aged"[Mesh] or elderly or post-menopause or postmenopause or "Postmenopause"[Mesh])

AND (HIV or "HIV"[Mesh] or “Human immunodeficiency virus” or "HIV Infections"[Mesh] or (“Acquired Immunodeficiency Syndrome”) or "Acquired Immunodeficiency Syndrome"[Mesh])

AND (women or female or "Women"[Mesh])

AND ("Comorbidity"[Mesh] or comorbid*)

NOT ("Africa"[Mesh] OR (“addresses”[pt] or “autobiography”[pt] or “bibliography”[pt] or “biography”[pt] or “case reports”[pt] or “comment”[pt] or “congresses”[pt] or “dictionary”[pt] or “directory”[pt] or “editorial”[pt] or “festschrift”[pt] or “government publications”[pt] or “historical article”[pt] or “interview”[pt] or “lectures”[pt] or “legal cases”[pt] or “legislation”[pt] or “letter”[pt] or “news”[pt] or “newspaper article”[pt] or “patient education handout”[pt] or “periodical index”[pt] or "comment on" or review[tw] OR ("Animals"[Mesh] NOT "Humans"[Mesh]) OR rats[tw] or cow[tw] or cows[tw] or chicken*[tw] or horse[tw] or horses[tw] or mice[tw] or mouse[tw] or bovine[tw] or sheep or ovine or murinae))

Limit to Female

Limit to 2005-

Limit to humans

Limit to English

**Appendix B. Characteristics of women in included studies by area of interest**

**Table S1. Characteristics of women in papers measuring the impact of strategies for engaging resources, or assessing barriers to engaging resources (Area 1)**

| Characteristic | Papers reporting information  (% out of 32) | Median  (25th, 75th) | Range |
| --- | --- | --- | --- |
| Publication year | 32 (100) | 2013 (2010, 2014) | 2006 to 2016 |
| Number of included women (all ages) | 32 (100) | 70 (30, 157) | 15 to 1701 |
| Enrollment start year | 24 (75) | 2006 (2002, 2010) | 1994 to 2014 |
| Enrollment duration, years | 24 (75) | 2 (1, 4) | 1 to 6 |
| *Information on age* |  |  |  |
| Women, proportion >40 years* | 10 (31) | 79 (76, 100) | 48 to 100 |
| Mean age, years | 24 (75) | 45 (43, 47) | 36 to 57 |
| At or after menopause, % | 0 (0) | NA | NA |
| Contracted HIV at older age, % | 0 (0) | NA | NA |
| *Racial and ethnic descent* |  |  |  |
| White, % | 15 (47) | 14 (8, 30) | 1 to 50 |
| Black/African American, % | 28 (88) | 78 (59, 86) | 16 to 100 |
| Latino/Hispanic, % | 18 (56) | 15 (10, 28) | 2 to 55 |
| Asian, % | 0 (0) | NA | NA |
| Native American, % | 3 (9) | 7 (4, 9) | 4 to 9 |
| Pacific Islander, % | 1 (3) | 7 (7, 7) | 7 to 7 |
| Other racial/ethnic descent, % | 12 (38) | 6 (3, 15) | 1 to 100 |
| *Other sociodemographic factors* |  |  |  |
| With a social support circle, % | 7 (22) | 58 (47, 62) | 11 to 85 |
| Married/with partner, % | 12 (38) | 20 (16, 30) | 12 to 73 |
| Caring for a dependent, % | 7 (22) | 31 (24, 55) | 16 to 100 |
| With annual income <$10,000, % | 15 (47) | 64 (50, 76) | 33 to 94 |
| Illegal immigrants, % | 1 (3) | 48 (48, 48) | 48 to 48 |
| Unemployed, % | 13 (41) | 75 (68, 80) | 33 to 100 |
| Have not finished high school, % | 22 (69) | 39 (32, 50) | 7 to 80 |
| With history of incarceration, % | 1 (3) | 100 (100, 100) | 100 to 100 |
| Experiencing violence, % | 2 (6) | 17 (5, 29) | 5 to 29 |
| Using illegal substances, % | 8 (25) | 46 (27, 100) | 14 to 100 |
| With mental health problems, % | 7 (22) | 55 (38, 76) | 4 to 78 |
| *Insurance status* |  |  |  |
| Without insurance, % | 5 (16) | 27 (9, 35) | 8 to 39 |
| With public insurance, % | 7 (22) | 73 (61, 100) | 60 to 100 |
| With private insurance, % | 5 (16) | 15 (5, 15) | 4 to 18 |
| *Gender identity and sexual orientation* |  |  |  |
| Transgender women, % | 2 (6) | 50 (0, 100) | 0 to 100 |
| Lesbian or bisexual women, % | 5 (16) | 11 (7, 17) | 0 to 33 |
| Without sexual concurrency, % | 0 (0) | NA | NA |

* Or older than another eligible age-cutoff

**Table S2. Characteristics of women in papers measuring the impact of insurance on outcomes (Area 2)**

| Characteristic | Papers reporting information  (% out of 7) | Median  (25th, 75th) | Range |
| --- | --- | --- | --- |
| Publication year | 7 (100) | 2013 (2009, 2015) | 2006 to 2015 |
| Number of included women (all ages) | 7 (100) | 164 (55, 748) | 17 to 1701 |
| Enrollment start year | 6 (86) | 2005 (1994, 2010) | 1994 to 2014 |
| Enrollment duration, years | 6 (86) | 2 (2, 4) | 1 to 4 |
| *Information on age* |  |  |  |
| Women, proportion >40 years* | 2 (29) | 74 (48, 100) | 48 to 100 |
| Mean age, years | 5 (71) | 43 (42, 46) | 41 to 50 |
| At or after menopause, % | 0 (0) | NA | NA |
| Contracted HIV at older age, % | 0 (0) | NA | NA |
| *Race/ethnicity* |  |  |  |
| White, % | 5 (71) | 18 (11, 32) | 1 to 36 |
| Black/African American, % | 7 (100) | 60 (55, 68) | 53 to 85 |
| Latino/Hispanic, % | 6 (86) | 26 (7, 30) | 3 to 35 |
| Asian, % | 0 (0) | NA | NA |
| Native American, % | 2 (29) | 6 (4, 9) | 4 to 9 |
| Pacific Islander, % | 0 (0) | NA | NA |
| Other, % | 3 (43) | 5 (2, 6) | 2 to 6 |
| *Other sociodemographic factors* |  |  |  |
| With a social support circle, % | 0 (0) | NA | NA |
| Married/with partner, % | 3 (43) | 16 (16, 34) | 16 to 34 |
| Caring for a dependent, % | 2 (29) | 27 (24, 31) | 24 to 31 |
| With annual income <$10,000, % | 3 (43) | 63 (50, 73) | 50 to 73 |
| Illegal immigrants, % | 0 (0) | NA | NA |
| Unemployed, % | 3 (43) | 70 (58, 78) | 58 to 78 |
| Have not finished high school, % | 5 (71) | 35 (35, 37) | 17 to 47 |
| With history of incarceration, % | 0 (0) | NA | NA |
| Experiencing violence, % | 1 (14) | 29 (29, 29) | 29 to 29 |
| Using illegal substances, % | 3 (43) | 28 (14, 38) | 14 to 38 |
| With mental health problems, % | 5 (71) | 51 (38, 55) | 4 to 100 |
| *Insurance status* |  |  |  |
| Without insurance, % | 4 (57) | 11 (8, 20) | 8 to 27 |
| With public insurance, % | 2 (29) | 67 (61, 73) | 61 to 73 |
| With private insurance, % | 3 (43) | 15 (15, 18) | 15 to 18 |
| *Gender identity and sexual orientation* |  |  |  |
| Transgender women, % | 0 (0) | NA | NA |
| Lesbian or bisexual women, % | 0 (0) | NA | NA |
| Without sexual concurrency, % | 0 (0) | NA | NA |

* Or older than another eligible age-cutoff

**Table S3. Characteristics of women in papers on the diagnosis or management of comorbidity, or of predictive models (Area 3)**

| Characteristic | Papers reporting information  (% out of 8) | Median  (25th, 75th) | Range |
| --- | --- | --- | --- |
| Publication year | 8 (100) | 2010 (2008, 2013) | 2006 to 2015 |
| Number of included women (all ages) | 8 (100) | 700 (137, 1101) | 126 to 1234 |
| Enrollment start year | 8 (100) | 1996 (1995, 1998) | 1994 to 2006 |
| Enrollment duration, years | 8 (100) | 4 (3, 8) | 2 to 10 |
| *Information on age* | 0 (0) | NA | NA |
| Women, proportion >40 years* | 7 (88) | 43 (38, 43) | 36 to 44 |
| Mean age, years | 0 (0) | NA | NA |
| At or after menopause, % | 0 (0) | NA | NA |
| Contracted HIV at older age, % | 4 (50) | 14 (13, 28) | 11 to 40 |
| *Race/ethnicity* | 7 (88) | 79 (53, 84) | 35 to 86 |
| White, % | 5 (62) | 15 (12, 30) | 12 to 30 |
| Black/African American, % | 1 (12) | 1 (1, 1) | 1 to 1 |
| Latino/Hispanic, % | 0 (0) | NA | NA |
| Asian, % | 0 (0) | NA | NA |
| Native American, % | 2 (25) | 13 (9, 16) | 9 to 16 |
| Pacific Islander, % | 3 (38) | 58 (33, 58) | 33 to 58 |
| Other, % | 0 (0) | NA | NA |
| *Other sociodemographic factors* | 0 (0) | NA | NA |
| With a social support circle, % | 4 (50) | 50 (50, 55) | 50 to 60 |
| Married/with partner, % | 0 (0) | NA | NA |
| Caring for a dependent, % | 2 (25) | 19 (5, 33) | 5 to 33 |
| With annual income <$10,000, % | 4 (50) | 57 (38, 75) | 35 to 75 |
| Illegal immigrants, % | 0 (0) | NA | NA |
| Unemployed, % | 0 (0) | NA | NA |
| Have not finished high school, % | 4 (50) | 100 (80, 100) | 59 to 100 |
| With history of incarceration, % | 3 (38) | 100 (32, 100) | 32 to 100 |
| Experiencing violence, % | 1 (12) | 14 (14, 14) | 14 to 14 |
| Using illegal substances, % | 1 (12) | 74 (74, 74) | 74 to 74 |
| With mental health problems, % | 0 (0) | NA | NA |
| *Insurance status* | 0 (0) | NA | NA |
| Without insurance, % | 0 (0) | NA | NA |
| With public insurance, % | 0 (0) | NA | NA |
| With private insurance, % | 8 (100) | 2010 (2008, 2013) | 2006 to 2015 |
| *Gender identity and sexual orientation* | 8 (100) | 700 (137, 1101) | 126 to 1234 |
| Transgender women, % | 8 (100) | 1996 (1995, 1998) | 1994 to 2006 |
| Lesbian or bisexual women, % | 8 (100) | 4 (3, 8) | 2 to 10 |
| Without sexual concurrency, % | 0 (0) | NA | NA |

* Or older than another eligible age-cutoff

Appendix C. Studies Excluded Because They Did Not Have an Analysis for HIV+ Women >40 Years old

| **PubMed ID** | **Title** | **Journal** | **Authors** | **Area 1 impact (yes/no)** | **Area 1 barriers (yes/no)** | **Area 2 (yes/no)** | **Area 3 comorbidities (yes/no)** | **Area 3 (what comorbidity)** | **Area 3 predictive model (yes/no)** |
| --- | --- | --- | --- | --- | --- | --- | --- | --- | --- |
| 25874644 | Patient Symptomatology in Anal Dysplasia | JAMA Surg | Hicks, C. W.; Wick, E. C.; Leeds, I. L.; Efron, J. E.; Gearhart, S. L.; Safar, B.; Fang, S. H. | no | no | no | yes | Anal dysplasia | no |
| 25005803 | Anemia in a cohort of HIV-infected Hispanics: prevalence, associated factors and impact on one-year mortality | BMC Res Notes | Santiago-Rodriguez, E. J.; Mayor, A. M.; Fernandez-Santos, D. M.; Ruiz-Candelaria, Y.; Hunter-Mellado, R. F. | no | no | no | no | Anemia | yes |
| 23664855 | A nationwide study of comorbidity and risk of reinfection after Staphylococcus aureus bacteraemia | J Infect | Wiese, L.; Mejer, N.; Schonheyder, H. C.; Westh, H.; Jensen, A. G.; Larsen, A. R.; Skov, R.; Benfield, T. | no | no | no | no | Bacteraemia | yes |
| 20940199 | Second malignancy risks after non-Hodgkin's lymphoma and chronic lymphocytic leukemia: differences by lymphoma subtype | J Clin Oncol | Morton, L. M.; Curtis, R. E.; Linet, M. S.; Bluhm, E. C.; Tucker, M. A.; Caporaso, N.; Ries, L. A.; Fraumeni, J. F., Jr. | no | no | no | yes | Cancer | yes |
| 16794245 | Liposomal doxorubicin, cyclophosphamide, and etoposide and antiretroviral therapy for patients with AIDS-related lymphoma: a pilot study | Oncologist | Combs, S.; Neil, N.; Aboulafia, D. M. | no | no | no | yes | Cancer | no |
| 25150257 | A new prognostic score for AIDS-related lymphomas in the rituximab-era | Haematologica | Barta, S. K.; Xue, X.; Wang, D.; Lee, J. Y.; Kaplan, L. D.; Ribera, J. M.; Oriol, A.; Spina, M.; Tirelli, U.; Boue, F.; Wilson, W. H.; Wyen, C.; Dunleavy, K.; Noy, A.; Sparano, J. A. | no | no | no | yes | Cancer | yes |
| 25251326 | Prognostic factors for advanced-stage human immunodeficiency virus-associated classical Hodgkin lymphoma treated with doxorubicin, bleomycin, vinblastine, and dacarbazine plus combined antiretroviral therapy: a multi-institutional retrospective study | Cancer | Castillo, J. J.; Bower, M.; Bruhlmann, J.; Novak, U.; Furrer, H.; Tanaka, P. Y.; Besson, C.; Montoto, S.; Cwynarski, K.; Abramson, J. S.; Dalia, S.; Bibas, M.; Connors, J. M.; Furman, M.; Nguyen, M. L.; Cooley, T. P.; Beltran, B. E.; Collins, J. A.; Vose, J. M.; Xicoy, B.; Ribera, J. M. | no | no | no | yes | Cancer | yes |
| 23595542 | The epidemic of non-Hodgkin lymphoma in the United States: disentangling the effect of HIV, 1992-2009 | Cancer Epidemiol Biomarkers Prev | Shiels, M. S.; Engels, E. A.; Linet, M. S.; Clarke, C. A.; Li, J.; Hall, H. I.; Hartge, P.; Morton, L. M. | no | no | no | yes | Cancer | yes |
| 20653483 | Cervical cancer screening among HIV-infected women in a health department setting | AIDS Patient Care STDS | Logan, J. L.; Khambaty, M. Q.; D'Souza, K. M.; Menezes, L. J. | no | yes | yes | yes | Cancer | no |
| 20859159 | Changes in knowledge of cervical cancer prevention and human papillomavirus among women with human immunodeficiency virus | Obstet Gynecol | Massad, L. S.; Evans, C. T.; Weber, K. M.; Goderre, J. L.; Hessol, N. A.; Henry, D.; Colie, C.; Strickler, H. D.; Watts, D. H.; Wilson, T. E. | no | no | no | yes | Cancer | yes |
| 19003664 | Correlation of cervical cytology and visual inspection with acetic acid in HIV-positive women | J Obstet Gynaecol | Akinwuntan, A. L.; Adesina, O. A.; Okolo, C. A.; Oluwasola, O. A.; Oladokun, A.; Ifemeje, A. A.; Adewole, I. F. | no | no | no | yes | Cancer | no |
| 22267344 | Perceptions of HPV and cervical cancer among Haitian immigrant women: implications for vaccine acceptability | Educ Health (Abingdon) | Kobetz, E.; Menard, J.; Hazan, G.; Koru-Sengul, T.; Joseph, T.; Nissan, J.; Barton, B.; Blanco, J.; Kornfeld, J. | yes | yes | no | yes | Cancer | no |
| 22736272 | The risk of hepatocellular carcinoma among individuals with acquired immunodeficiency syndrome in the United States | Cancer | Sahasrabuddhe, V. V.; Shiels, M. S.; McGlynn, K. A.; Engels, E. A. | no | no | no | yes | Cancer | yes |
| 18669717 | Cervical intraepithelial neoplasia grade 2 or worse in human immunodeficiency virus-infected women with mildly abnormal cervical cytology | Obstet Gynecol | Boardman, L. A.; Cotter, K.; Raker, C.; Cu-Uvin, S. | no | no | yes | yes | Cancer | yes |
| 21339382 | Evaluation of the addition of rituximab to CODOX-M/IVAC for Burkitt's lymphoma: a retrospective analysis | Ann Oncol | Barnes, J. A.; Lacasce, A. S.; Feng, Y.; Toomey, C. E.; Neuberg, D.; Michaelson, J. S.; Hochberg, E. P.; Abramson, J. S. | no | no | no | yes | Cancer | no |
| 22011239 | Minimally abnormal Pap testing and cervical histology in HIV-infected women | J Womens Health (Larchmt) | Curry, C. L.; Sage, Y. H.; Vragovic, O.; Stier, E. A. | no | no | no | yes | Cancer | no |
| 20605046 | Outcomes after an excisional procedure for cervical intraepithelial neoplasia in HIV-infected women | Gynecol Oncol | Reimers, L. L.; Sotardi, S.; Daniel, D.; Chiu, L. G.; Van Arsdale, A.; Wieland, D. L.; Leider, J. M.; Xue, X.; Strickler, H. D.; Garry, D. J.; Goldberg, G. L.; Einstein, M. H. | no | no | no | yes | Cancer | no |
| 17415113 | Outcomes after treatment of cervical intraepithelial neoplasia among women with HIV | J Low Genit Tract Dis | Massad, L. S.; Fazzari, M. J.; Anastos, K.; Klein, R. S.; Minkoff, H.; Jamieson, D. J.; Duerr, A.; Celentano, D.; Gange, S.; Cu-Uvin, S.; Young, M.; Watts, D. H.; Levine, A. M.; Schuman, P.; Harris, T. G.; Strickler, H. D. | no | no | no | yes | Cancer | no |
| 15914552 | Rituximab does not improve clinical outcome in a randomized phase 3 trial of CHOP with or without rituximab in patients with HIV-associated non-Hodgkin lymphoma: AIDS-Malignancies Consortium Trial 010 | Blood | Kaplan, L. D.; Lee, J. Y.; Ambinder, R. F.; Sparano, J. A.; Cesarman, E.; Chadburn, A.; Levine, A. M.; Scadden, D. T. | no | no | no | yes | Cancer | no |
| 22308010 | Rituximab in combination with chemotherapy versus chemotherapy alone in HIV-associated non-Hodgkin lymphoma: a pooled analysis of 15 prospective studies | Am J Hematol | Castillo, J. J.; Echenique, I. A. | no | no | no | yes | Cancer | no |
| 18760524 | Surgical management of cervical intraepithelial neoplasia in HIV-infected women | Eur J Obstet Gynecol Reprod Biol | Foulot, H.; Heard, I.; Potard, V.; Costagliola, D.; Chapron, C. | no | no | no | yes | Cancer | no |
| 15875183 | Decreased number of granzyme B+ activated CD8+ cytotoxic T lymphocytes in the inflammatory background of HIV-associated Hodgkin's lymphoma | Ann Hematol | Bosch Princep, R.; Lejeune, M.; Salvado Usach, M. T.; Jaen Martinez, J.; Pons Ferre, L. E.; Alvaro Naranjo, T. | no | no | no | no | Cancer | yes |
| 21139489 | The effect of HAART and calendar period on Kaposi's sarcoma and non-Hodgkin lymphoma: results of a match between an AIDS and cancer registry | Aids | Pipkin, S.; Scheer, S.; Okeigwe, I.; Schwarcz, S.; Harris, D. H.; Hessol, N. A. | no | no | no | no | Cancer | yes |
| 23332770 | Outcome of hepatocellular carcinoma in human immunodeficiency virus-infected patients | Dig Liver Dis | Gramenzi, A.; Tedeschi, S.; Cantarini, M. C.; Erroi, V.; Tumietto, F.; Attard, L.; Calza, L.; Foschi, F. G.; Caraceni, P.; Pavoni, M.; Cucchetti, A.; Bernardi, M.; Viale, P.; Verucchi, G.; Trevisani, F. | no | no | no | no | Cancer | yes |
| 20861396 | Trends in Kaposi's sarcoma survival disparities in the United States: 1980 through 2004 | Cancer Epidemiol Biomarkers Prev | Datta, G. D.; Kawachi, I.; Delpierre, C.; Lang, T.; Grosclaude, P. | no | no | no | no | Cancer | yes |
| 23079809 | Disparities in the treatment and outcomes of lung cancer among HIV-infected individuals | Aids | Suneja, G.; Shiels, M. S.; Melville, S. K.; Williams, M. A.; Rengan, R.; Engels, E. A. | no | no | no | no | Cancer | yes |
| 24220684 | Do HIV-infected non-small cell lung cancer patients receive guidance-concordant care? | Med Care | Lee, J. Y.; Moore, P. C.; Steliga, M. A. | no | no | no | no | Cancer | yes |
| 26077242 | Elevated Cancer-Specific Mortality Among HIV-Infected Patients in the United States | J Clin Oncol | Coghill, A. E.; Shiels, M. S.; Suneja, G.; Engels, E. A. | no | no | no | no | Cancer | yes |
| 22422679 | Evaluation of the impact of HIV serostatus, tobacco smoking and CD4 counts on epidermoid anal cancer survival | Int J STD AIDS | Linam, J. M.; Chand, R. R.; Broudy, V. C.; Liu, K. C.; Back, A. L.; Lin, E. H.; Patel, S. A. | no | no | no | no | Cancer | yes |
| 22697293 | High survivin expression as a risk factor in patients with anal carcinoma treated with concurrent chemoradiotherapy | Radiat Oncol | Fraunholz, I.; Rodel, C.; Distel, L.; Rave-Frank, M.; Kohler, D.; Falk, S.; Rodel, F. | no | no | no | no | Cancer | yes |
| 21160411 | Mortality after cancer diagnosis in HIV-infected individuals treated with antiretroviral therapy | Aids | Achenbach, C. J.; Cole, S. R.; Kitahata, M. M.; Casper, C.; Willig, J. H.; Mugavero, M. J.; Saag, M. S. | no | no | no | no | Cancer | yes |
| 18332290 | Ongoing improvement in outcomes for patients diagnosed as having Non-Hodgkin lymphoma from the 1990s to the early 21st century | Arch Intern Med | Pulte, D.; Gondos, A.; Brenner, H. | no | no | no | no | Cancer | yes |
| 20526793 | Primary CNS lymphoma in HIV positive and negative patients: comparison of clinical characteristics, outcome and prognostic factors | J Neurooncol | Bayraktar, S.; Bayraktar, U. D.; Ramos, J. C.; Stefanovic, A.; Lossos, I. S. | no | no | no | no | Cancer | yes |
| 20023215 | Rituximab plus concurrent infusional EPOCH chemotherapy is highly effective in HIV-associated B-cell non-Hodgkin lymphoma | Blood | Sparano, J. A.; Lee, J. Y.; Kaplan, L. D.; Levine, A. M.; Ramos, J. C.; Ambinder, R. F.; Wachsman, W.; Aboulafia, D.; Noy, A.; Henry, D. H.; Von Roenn, J.; Dezube, B. J.; Remick, S. C.; Shah, M. H.; Leichman, L.; Ratner, L.; Cesarman, E.; Chadburn, A.; Mitsuyasu, R. | no | no | no | no | Cancer | yes |
| 22112600 | Treatment of AIDS-related lymphomas: rituximab is beneficial even in severely immunosuppressed patients | Aids | Wyen, C.; Jensen, B.; Hentrich, M.; Siehl, J.; Sabranski, M.; Esser, S.; Gillor, D.; Muller, M.; Van Lunzen, J.; Wolf, T.; Bogner, J. R.; Wasmuth, J. C.; Christ, H.; Fatkenheuer, G.; Hoffmann, C. | no | no | no | no | Cancer | yes |
| 19203845 | Outcomes of chemoradiotherapy with 5-Fluorouracil and mitomycin C for anal cancer in immunocompetent versus immunodeficient patients | Int J Radiat Oncol Biol Phys | Seo, Y.; Kinsella, M. T.; Reynolds, H. L.; Chipman, G.; Remick, S. C.; Kinsella, T. J. | no | no | no | no | Cancer | yes |
| 16280699 | Predictors of mortality for patients with advanced disease in an HIV palliative care program | J Acquir Immune Defic Syndr | Shen, J. M.; Blank, A.; Selwyn, P. A. | no | no | no | no | Cancer, pneumonia | yes |
| 24121754 | High risk of obesity and weight gain for HIV-infected uninsured minorities | J Acquir Immune Defic Syndr | Taylor, B. S.; Liang, Y.; Garduno, L. S.; Walter, E. A.; Gerardi, M. B.; Anstead, G. M.; Bullock, D.; Turner, B. J. | no | no | no | yes | Cardiovascular disease | yes |
| 16914390 | Heart positive: design of a randomized controlled clinical trial of intensive lifestyle intervention, niacin and fenofibrate for HIV lipodystrophy/dyslipidemia | Contemp Clin Trials | Samson, S. L.; Pownall, H. J.; Scott, L. W.; Ballantyne, C. M.; Smith, E. O.; Sekhar, R. V.; Balasubramanyam, A. | no | no | no | yes | Cardiovascular disease | no |
| 25324353 | HIV infection and cardiovascular disease in women | J Am Heart Assoc | Womack, J. A.; Chang, C. C.; So-Armah, K. A.; Alcorn, C.; Baker, J. V.; Brown, S. T.; Budoff, M.; Butt, A. A.; Gibert, C.; Goetz, M. B.; Gottdiener, J.; Gottlieb, S.; Justice, A. C.; Leaf, D.; McGinnis, K.; Rimland, D.; Rodriguez-Barradas, M. C.; Sico, J.; Skanderson, M.; Tindle, H.; Tracy, R. P.; Warner, A.; Freiberg, M. S. | no | no | no | yes | Cardiovascular disease | yes |
| 22942209 | Underutilization of aspirin for primary prevention of cardiovascular disease among HIV-infected patients | Clin Infect Dis | Burkholder, G. A.; Tamhane, A. R.; Salinas, J. L.; Mugavero, M. J.; Raper, J. L.; Westfall, A. O.; Saag, M. S.; Willig, J. H. | no | no | no | yes | Cardiovascular disease | no |
| 21565796 | Combination of niacin and fenofibrate with lifestyle changes improves dyslipidemia and hypoadiponectinemia in HIV patients on antiretroviral therapy: results of 'heart positive,' a randomized, controlled trial | J Clin Endocrinol Metab | Balasubramanyam, A.; Coraza, I.; Smith, E. O.; Scott, L. W.; Patel, P.; Iyer, D.; Taylor, A. A.; Giordano, T. P.; Sekhar, R. V.; Clark, P.; Cuevas-Sanchez, E.; Kamble, S.; Ballantyne, C. M.; Pownall, H. J. | no | no | no | yes | Cardiovascular disease | yes |
| 22762716 | Comparison of in-hospital mortality from acute myocardial infarction in HIV sero-positive versus sero-negative individuals | Am J Cardiol | Pearce, D.; Ani, C.; Espinosa-Silva, Y.; Clark, R.; Fatima, K.; Rahman, M.; Diebolt, E.; Ovbiagele, B. | no | no | no | yes | Cardiovascular disease | yes |
| 20597691 | Low CD4+ T cell count is a risk factor for cardiovascular disease events in the HIV outpatient study | Clin Infect Dis | Lichtenstein, K. A.; Armon, C.; Buchacz, K.; Chmiel, J. S.; Buckner, K.; Tedaldi, E. M.; Wood, K.; Holmberg, S. D.; Brooks, J. T. | no | no | no | yes | Cardiovascular disease | yes |
| 18620492 | Peripheral arterial disease in HIV patients older than 50 years of age | AIDS Res Hum Retroviruses | Palacios, R.; Alonso, I.; Hidalgo, A.; Aguilar, I.; Sanchez, M. A.; Valdivielso, P.; Gonzalez-Santos, P.; Santos, J. | no | no | no | yes | Cardiovascular disease | yes |
| 24870935 | Severity of cardiovascular disease outcomes among patients with HIV is related to markers of inflammation and coagulation | J Am Heart Assoc | Nordell, A. D.; McKenna, M.; Borges, A. H.; Duprez, D.; Neuhaus, J.; Neaton, J. D. | no | no | no | yes | Cardiovascular disease | yes |
| 104175579. Language: | Hospitalizations for Cardiovascular Disease in African Americans and Whites with HIV/AIDS | Population Health Management | Oramasionwu, Christine U.; Morse, Gene D.; Lawson, Kenneth A.; Brown, Carolyn M.; Koeller, Jim M.; Frei, Christopher R. | no | no | no | yes | Cardiovascular disease | yes |
| 26358261 | Depression and human immunodeficiency virus infection are risk factors for incident heart failure among veterans: Veterans Aging Cohort Study | Circulation | White, J. R. | no | no | no | yes | Cardiovascular disease | yes |
| 23386631 | Effect of statin therapy in reducing the risk of serious non-AIDS-defining events and nonaccidental death | Clin Infect Dis | Overton, E. T.; Kitch, D.; Benson, C. A.; Hunt, P. W.; Stein, J. H.; Smurzynski, M.; Ribaudo, H. J.; Tebas, P. | no | no | no | yes | Cardiovascular disease | yes |
| 105434102. Language: | Efficacy and safety of atazanavir-ritonavir plus abacavir-lamivudine or tenofovir-emtricitabine in patients with hyperlipidaemia switched from a stable protease inhibitor-based regimen including one thymidine analogue | AIDS Patient Care & STDs | Calza, L.; Manfredi, R.; Colangeli, V.; Pocaterra, D.; Rosseti, N.; Pavoni, M.; Chiodo, F. | no | no | no | yes | Cardiovascular disease | no |
| 22866506 | Cardiovascular health of HIV-infected African-American women at the Miriam Hospital Immunology Center in Providence, RI | Med Health R I | Zachary, D.; Gillani, F. S.; Najfi, N.; Casarella, R.; Tashima, K. | no | no | no | yes | Cardiovascular disease | no |
| 25588033 | HIV infection, cardiovascular disease risk factor profile, and risk for acute myocardial infarction | J Acquir Immune Defic Syndr | Paisible, A. L.; Chang, C. C.; So-Armah, K. A.; Butt, A. A.; Leaf, D. A.; Budoff, M.; Rimland, D.; Bedimo, R.; Goetz, M. B.; Rodriguez-Barradas, M. C.; Crane, H. M.; Gibert, C. L.; Brown, S. T.; Tindle, H. A.; Warner, A. L.; Alcorn, C.; Skanderson, M.; Justice, A. C.; Freiberg, M. S. | no | no | no | yes | Cardiovascular disease | no |
| 17453589 | Protease inhibitors and cardiovascular disease: analysis of the Los Angeles County adult spectrum of disease cohort | AIDS Care | Vaughn, G.; Detels, R. | no | no | no | yes | Cardiovascular disease | no |
| 22739396 | Effects of rosuvastatin versus pravastatin on low-density lipoprotein diameter in HIV-1-infected patients receiving ritonavir-boosted protease inhibitor | Aids | Bittar, R.; Giral, P.; Aslangul, E.; Assoumou, L.; Valantin, M. A.; Kalmykova, O.; Federspiel, M. C.; Cherfils, C.; Costagliola, D.; Bonnefont-Rousselot, D. | no | no | no | yes | Cardiovascular disease | no |
| 24065316 | Prehypertension, hypertension, and the risk of acute myocardial infarction in HIV-infected and -uninfected veterans | Clin Infect Dis | Armah, K. A.; Chang, C. C.; Baker, J. V.; Ramachandran, V. S.; Budoff, M. J.; Crane, H. M.; Gibert, C. L.; Goetz, M. B.; Leaf, D. A.; McGinnis, K. A.; Oursler, K. K.; Rimland, D.; Rodriguez-Barradas, M. C.; Sico, J. J.; Warner, A. L.; Hsue, P. Y.; Kuller, L. H.; Justice, A. C.; Freiberg, M. S. | no | no | no | yes | Cardiovascular disease | no |
| 16770290 | Antiretroviral therapy in HIV-positive women is associated with increased apolipoproteins and total cholesterol | J Acquir Immune Defic Syndr | Rimland, D.; Guest, J. L.; Hernandez-Ramos, I.; Del Rio, C.; Le, N. A.; Brown, W. V. | no | no | no | yes | Cardiovascular disease | yes |
| 20078196 | Paraoxonase-1 gene haplotypes are associated with metabolic disturbances, atherosclerosis, and immunologic outcome in HIV-infected patients | J Infect Dis | Parra, S.; Marsillach, J.; Aragones, G.; Beltran, R.; Montero, M.; Coll, B.; Mackness, B.; Mackness, M.; Alonso-Villaverde, C.; Joven, J.; Camps, J. | no | no | no | yes | Cardiovascular disease | yes |
| 20626901 | Low cholesterol? Don't brag yet ... hypocholesterolemia blunts HAART effectiveness: a longitudinal study | J Int AIDS Soc | Miguez, M. J.; Lewis, J. E.; Bryant, V. E.; Rosenberg, R.; Burbano, X.; Fishman, J.; Asthana, D.; Duan, R.; Madhavan, N.; Malow, R. M. | no | no | no | yes | Cardiovascular disease | yes |
| 16954725 | Effects of a lifestyle modification program in HIV-infected patients with the metabolic syndrome | Aids | Fitch, K. V.; Anderson, E. J.; Hubbard, J. L.; Carpenter, S. J.; Waddell, W. R.; Caliendo, A. M.; Grinspoon, S. K. | yes | no | no | yes | Cardiovascular disease | no |
| 22828718 | Metabolic syndrome before and after initiation of antiretroviral therapy in treatment-naive HIV-infected individuals | J Acquir Immune Defic Syndr | Krishnan, S.; Schouten, J. T.; Atkinson, B.; Brown, T.; Wohl, D.; McComsey, G. A.; Glesby, M. J.; Shikuma, C.; Haubrich, R.; Tebas, P.; Campbell, T. B.; Jacobson, D. L. | no | no | no | yes | Cardiovascular disease | yes |
| 25414089 | Venous thromboembolism among HIV-positive patients and anticoagulation clinic outcomes integrated within the HIV primary care setting | Int J STD AIDS | Modi, R. A. | no | yes | no | yes | Cardiovascular disease | no |
| 21450595 | Are HIV patients undertreated? Cardiovascular risk factors in HIV: results of the HIV-HEART study | Eur J Prev Cardiol | Reinsch, N.; Neuhaus, K.; Esser, S.; Potthoff, A.; Hower, M.; Mostardt, S.; Neumann, A.; Brockmeyer, N. H.; Gelbrich, G.; Erbel, R.; Neumann, T. | no | no | no | yes | Cardiovascular disease | yes |
| 21189273 | Comparative Effectiveness and Toxicity of Statins Among HIV-Infected Patients | Clin Infect Dis | Singh, S.; Willig, J. H.; Mugavero, M. J.; Crane, P. K.; Harrington, R. D.; Knopp, R. H.; Kosel, B. W.; Saag, M. S.; Kitahata, M. M.; Crane, H. M. | no | no | no | yes | Cardiovascular disease | no |
| 16218799 | A randomized trial of the efficacy and safety of fenofibrate versus pravastatin in HIV-infected subjects with lipid abnormalities: AIDS Clinical Trials Group Study 5087 | AIDS Res Hum Retroviruses | Aberg, J. A.; Zackin, R. A.; Brobst, S. W.; Evans, S. R.; Alston, B. L.; Henry, W. K.; Glesby, M. J.; Torriani, F. J.; Yang, Y.; Owens, S. I.; Fichtenbaum, C. J. | no | no | no | yes | Cardiovascular disease | no |
| 21248273 | Increasing incidence of ischemic stroke in patients with HIV infection | Neurology | Ovbiagele, B.; Nath, A. | no | no | no | no | Cardiovascular disease | yes |
| 23604043 | Validation of Medicaid Claims-based Diagnosis of Myocardial Infarction Using an HIV Clinical Cohort | Medical Care | Brouwer, Emily S.; Napravnik, Sonia; Eron Jr, Joseph J.; Simpson Jr, Ross J.; Brookhart, M. Alan; Stalzer, Brant; Vinikoor, Michael; Floris-Moore, Michelle; StÃ¼rmer, Til | no | no | no | no | Cardiovascular disease | yes |
| 23062726 | Validation of the Registry to Evaluate Early and Long-Term Pulmonary Arterial Hypertension Disease Management (REVEAL) pulmonary hypertension prediction model in a unique population and utility in the prediction of long-term survival | J Heart Lung Transplant | Cogswell, R.; Kobashigawa, E.; McGlothlin, D.; Shaw, R.; De Marco, T. | no | no | no | no | Cardiovascular disease | yes |
| 16630034 | The use of the Framingham equation to predict myocardial infarctions in HIV-infected patients: comparison with observed events in the D:A:D Study | HIV Med | Law, M. G.; Friis-Moller, N.; El-Sadr, W. M.; Weber, R.; Reiss, P.; D'Arminio Monforte, A.; Thiebaut, R.; Morfeldt, L.; De Wit, S.; Pradier, C.; Calvo, G.; Kirk, O.; Sabin, C. A.; Phillips, A. N.; Lundgren, J. D. | no | no | no | no | Cardiovascular disease | yes |
| 19515371 | Lipoprotein particle subclasses, cardiovascular disease and HIV infection | Atherosclerosis | Duprez, D. A.; Kuller, L. H.; Tracy, R.; Otvos, J.; Cooper, D. A.; Hoy, J.; Neuhaus, J.; Paton, N. I.; Friis-Moller, N.; Lampe, F.; Liappis, A. P.; Neaton, J. D. | no | no | no | no | Cardiovascular disease | yes |
| 23651103 | Metabolic syndrome predicts all-cause mortality in persons with human immunodeficiency virus | AIDS Patient Care STDS | Jarrett, O. D.; Wanke, C. A.; Ruthazer, R.; Bica, I.; Isaac, R.; Knox, T. A. | no | no | no | no | Cardiovascular disease | yes |
| 21251183 | Rates of cardiovascular disease following smoking cessation in patients with HIV infection: results from the D:A:D study(*) | HIV Med | Petoumenos, K.; Worm, S.; Reiss, P.; de Wit, S.; d'Arminio Monforte, A.; Sabin, C.; Friis-Moller, N.; Weber, R.; Mercie, P.; Pradier, C.; El-Sadr, W.; Kirk, O.; Lundgren, J.; Law, M. | no | no | no | no | Cardiovascular disease | yes |
| 22313955 | Association of tenofovir exposure with kidney disease risk in HIV infection | Aids | Scherzer, R.; Estrella, M.; Li, Y.; Choi, A. I.; Deeks, S. G.; Grunfeld, C.; Shlipak, M. G. | no | no | no | yes | Cardiovascular disease, substance abuse | no |
| 25173116 | Outcomes of patients with human immunodeficiency virus infection undergoing cardiovascular surgery in the United States | J Thorac Cardiovasc Surg | Robich, M. P.; Schiltz, N.; Johnston, D. R.; Mick, S.; Tse, W.; Koch, C.; Soltesz, E. G. | no | no | no | yes | Cardiovascular disease, substance abuse | no |
| 24637542 | HIV protease inhibitor exposure predicts cerebral small vessel disease | Aids | Soontornniyomkij, V.; Umlauf, A.; Chung, S. A.; Cochran, M. L.; Soontornniyomkij, B.; Gouaux, B.; Toperoff, W.; Moore, D. J.; Masliah, E.; Ellis, R. J.; Grant, I.; Achim, C. L. | no | no | no | yes | Cerebral small vessel disease | yes |
| 103751175. Language: | Examining Associations Between Cognitive-Affective Vulnerability and HIV Symptom Severity, Perceived Barriers to Treatment Adherence, and Viral Load Among HIV-Positive Adults | International Journal of Behavioral Medicine | Leyro, Teresa; Vujanovic, Anka; Bonn-Miller, Marcel | no | yes | no | no | cognitive impariment | yes |
| 18042509 | Selegiline transdermal system (STS) for HIV-associated cognitive impairment: open-label report of ACTG 5090 | HIV Clin Trials | Evans, S. R.; Yeh, T. M.; Sacktor, N.; Clifford, D. B.; Simpson, D.; Miller, E. N.; Ellis, R. J.; Valcour, V.; Marra, C. M.; Millar, L.; Schifitto, G. | no | no | no | yes | cognitive impariment | no |
| 16840235 | Timed Gait test: normative data for the assessment of the AIDS dementia complex | J Clin Exp Neuropsychol | Robertson, K. R.; Parsons, T. D.; Sidtis, J. J.; Hanlon Inman, T.; Robertson, W. T.; Hall, C. D.; Price, R. W. | no | no | no | yes | Cognitive impariment | no |
| 17852582 | Predictive validity of demographically adjusted normative standards for the HIV Dementia Scale | J Clin Exp Neuropsychol | Morgan, E. E.; Woods, S. P.; Scott, J. C.; Childers, M.; Beck, J. M.; Ellis, R. J.; Grant, I.; Heaton, R. K. | no | no | no | yes | Cognitive impariment | no |
| 17721095 | Memantine and HIV-associated cognitive impairment: a neuropsychological and proton magnetic resonance spectroscopy study | Aids | Schifitto, G.; Navia, B. A.; Yiannoutsos, C. T.; Marra, C. M.; Chang, L.; Ernst, T.; Jarvik, J. G.; Miller, E. N.; Singer, E. J.; Ellis, R. J.; Kolson, D. L.; Simpson, D.; Nath, A.; Berger, J.; Shriver, S. L.; Millar, L. L.; Colquhoun, D.; Lenkinski, R.; Gonzalez, R. G.; Lipton, S. A. | no | no | no | yes | Cognitive impariment | no |
| 22067661 | A randomized, double-blind, controlled study of NGX-4010, a capsaicin 8% dermal patch, for the treatment of painful HIV-associated distal sensory polyneuropathy | J Acquir Immune Defic Syndr | Clifford, D. B.; Simpson, D. M.; Brown, S.; Moyle, G.; Brew, B. J.; Conway, B.; Tobias, J. K.; Vanhove, G. F. | no | no | no | yes | Cognitive impariment | no |
| 16271919 | Nephrotic range proteinuria and CD4 count as noninvasive indicators of HIV-associated nephropathy | Am J Med | Atta, M. G.; Choi, M. J.; Longenecker, J. C.; Haymart, M.; Wu, J.; Nagajothi, N.; Racusen, L. C.; Scheel, P. J., Jr.; Brancati, F. L.; Fine, D. M. | no | no | no | yes | Cognitive impariment | no |
| 18154490 | Spanish validation of the HIV dementia scale in women | AIDS Patient Care STDS | Wojna, V.; Skolasky, R. L.; McArthur, J. C.; Maldonado, E.; Hechavarria, R.; Mayo, R.; Selnes, O.; Ginebra, T.; de la Torre, T.; Garcia, H.; Kraiselburd, E.; Melendez-Guerrero, L. M.; Zorrilla, C. D.; Nath, A. | no | no | no | yes | Cognitive impariment | no |
| 24135776 | A brief and feasible paper-based method to screen for neurocognitive impairment in HIV-infected patients: the NEU screen | J Acquir Immune Defic Syndr | Munoz-Moreno, J. A.; Prats, A.; Perez-Alvarez, N.; Fumaz, C. R.; Garolera, M.; Doval, E.; Negredo, E.; Ferrer, M. J.; Clotet, B. | no | no | no | yes | Cognitive impariment | no |
| 18844464 | Nadir CD4 cell count predicts neurocognitive impairment in HIV-infected patients | AIDS Res Hum Retroviruses | Munoz-Moreno, J. A.; Fumaz, C. R.; Ferrer, M. J.; Prats, A.; Negredo, E.; Garolera, M.; Perez-Alvarez, N.; Molto, J.; Gomez, G.; Clotet, B. | no | no | no | yes | Cognitive impariment | yes |
| 20509050 | Psychometric evaluation of the functional assessment of HIV Infection (FAHI) questionnaire and its usefulness in clinical trials | Qual Life Res | Viala-Danten, M.; Dubois, D.; Gilet, H.; Martin, S.; Peeters, K.; Cella, D. | no | no | no | yes | Cognitive impariment | no |
| 20400412 | Memantine for AIDS dementia complex: open-label report of ACTG 301 | HIV Clin Trials | Zhao, Y.; Navia, B. A.; Marra, C. M.; Singer, E. J.; Chang, L.; Berger, J.; Ellis, R. J.; Kolson, D. L.; Simpson, D.; Miller, E. N.; Lipton, S. A.; Evans, S. R.; Schifitto, G. | no | no | no | yes | Cognitive impariment | yes |
| 108095547. Language: | Randomized trial of minocycline in the treatment of HIV-associated cognitive impairment | Neurology | Nakasujja, N.; Miyahara, S.; Evans, S.; Lee, A.; Musisi, S.; Katabira, E.; Robertson, K.; Ronald, A.; Clifford, D. B.; Sacktor, N. | no | no | no | yes | Cognitive impariment | yes |
| 25201556 | A history of alcohol dependence augments HIV-associated neurocognitive deficits in persons aged 60 and older | J Neurovirol | Gongvatana, A.; Morgan, E. E.; Iudicello, J. E.; Letendre, S. L.; Grant, I.; Woods, S. P. | no | no | no | yes | Cognitive impariment | no |
| 17652642 | A multicenter trial of selegiline transdermal system for HIV-associated cognitive impairment | Neurology | Schifitto, G.; Zhang, J.; Evans, S. R.; Sacktor, N.; Simpson, D.; Millar, L. L.; Hung, V. L.; Miller, E. N.; Smith, E.; Ellis, R. J.; Valcour, V.; Singer, E.; Marra, C. M.; Kolson, D.; Weihe, J.; Remmel, R.; Katzenstein, D.; Clifford, D. B. | no | no | no | yes | Cognitive impariment | no |
| 24554483 | An active lifestyle is associated with better neurocognitive functioning in adults living with HIV infection | J Neurovirol | Fazeli, P. L.; Woods, S. P.; Heaton, R. K.; Umlauf, A.; Gouaux, B.; Rosario, D.; Moore, R. C.; Grant, I.; Moore, D. J. | no | no | no | yes | Cognitive impariment | no |
| 23111573 | Concurrent classification accuracy of the HIV dementia scale for HIV-associated neurocognitive disorders in the CHARTER Cohort | J Acquir Immune Defic Syndr | Sakamoto, M.; Marcotte, T. D.; Umlauf, A.; Franklin, D., Jr.; Heaton, R. K.; Ellis, R. J.; Letendre, S.; Alexander, T.; McCutchan, J. A.; Morgan, E. E.; Woods, S. P.; Collier, A. C.; Marra, C. M.; Clifford, D. B.; Gelman, B. B.; McArthur, J. C.; Morgello, S.; Simpson, D.; Grant, I. | no | no | no | yes | Cognitive impariment | no |
| 17950469 | CSF proteomic fingerprints for HIV-associated cognitive impairment | J Neuroimmunol | Laspiur, J. P.; Anderson, E. R.; Ciborowski, P.; Wojna, V.; Rozek, W.; Duan, F.; Mayo, R.; Rodriguez, E.; Plaud-Valentin, M.; Rodriguez-Orengo, J.; Gendelman, H. E.; Melendez, L. M. | no | no | no | yes | Cognitive impariment | no |
| 21900636 | Minocycline treatment for HIV-associated cognitive impairment: results from a randomized trial | Neurology | Sacktor, N.; Miyahara, S.; Deng, L.; Evans, S.; Schifitto, G.; Cohen, B. A.; Paul, R.; Robertson, K.; Jarocki, B.; Scarsi, K.; Coombs, R. W.; Zink, M. C.; Nath, A.; Smith, E.; Ellis, R. J.; Singer, E.; Weihe, J.; McCarthy, S.; Hosey, L.; Clifford, D. B. | no | no | no | yes | Cognitive impariment | no |
| 19937504 | Modafinil effects on cognitive function in HIV+ patients treated for fatigue: a placebo controlled study | J Clin Exp Neuropsychol | McElhiney, M.; Rabkin, J.; Van Gorp, W.; Rabkin, R. | no | no | no | yes | Cognitive impariment | no |
| 22648011 | Selective neurocognitive deficits and poor life functioning are associated with significant depressive symptoms in alcoholism-HIV infection comorbidity | Psychiatry Res | Sassoon, S. A.; Rosenbloom, M. J.; Fama, R.; Sullivan, E. V.; Pfefferbaum, A. | no | no | no | yes | Cognitive impariment | no |
| 24378603 | Visuospatial temporal order memory deficits in older adults with HIV infection | Cogn Behav Neurol | Woods, S. P.; Hoebel, C.; Pirogovsky, E.; Rooney, A.; Cameron, M. V.; Grant, I.; Gilbert, P. E. | no | no | no | yes | Cognitive impariment | no |
| 25600199 | Incidence and long-term outcomes of the human immunodeficiency virus neuroretinal disorder in patients with AIDS | Ophthalmology | Jabs, D. A.; Drye, L.; Van Natta, M. L.; Thorne, J. E.; Holland, G. N. | no | no | no | no | Cognitive impariment | yes |
| 17065131 | Lowest ever CD4 lymphocyte count (CD4 nadir) as a predictor of current cognitive and neurological status in human immunodeficiency virus type 1 infection--The Hawaii Aging with HIV Cohort | J Neurovirol | Valcour, V.; Yee, P.; Williams, A. E.; Shiramizu, B.; Watters, M.; Selnes, O.; Paul, R.; Shikuma, C.; Sacktor, N. | no | no | no | yes | Cognitive impariment | no |
| 19441980 | A phase II randomized trial of amphotericin B alone or combined with fluconazole in the treatment of HIV-associated cryptococcal meningitis | Clin Infect Dis | Pappas, P. G.; Chetchotisakd, P.; Larsen, R. A.; Manosuthi, W.; Morris, M. I.; Anekthananon, T.; Sungkanuparph, S.; Supparatpinyo, K.; Nolen, T. L.; Zimmer, L. O.; Kendrick, A. S.; Johnson, P.; Sobel, J. D.; Filler, S. G. | no | no | no | yes | Cryptococcal meningitis | yes |
| 23555970 | Predictors of mortality and differences in clinical features among patients with Cryptococcosis according to immune status | PLoS One | Brizendine, K. D.; Baddley, J. W.; Pappas, P. G. | no | no | no | yes | Cryptococcosis | yes |
| 24845948 | Assessment of the efficacy and safety of pre-emptive anti-cytomegalovirus (CMV) therapy in HIV-infected patients with CMV viraemia | Int J STD AIDS | Mattioni, S.; Pavie, J.; Porcher, R.; Scieux, C.; Denis, B.; De Castro, N.; Simon, F.; Molina, J. M. | no | no | no | yes | Cytomegalovirus | yes |
| 22265144 | Clinical outcomes in patients with cytomegalovirus retinitis treated with ganciclovir implant | Am J Ophthalmol | Oktavec, K. C.; Nolan, K.; Brown, D. M.; Dunn, J. P.; Livingston, A. G.; Thorne, J. E. | no | no | no | yes | Cytomegalovirus | no |
| 16258865 | Incidence and prognosis of CMV disease in HIV-infected patients before and after introduction of combination antiretroviral therapy | Infection | Salzberger, B.; Hartmann, P.; Hanses, F.; Uyanik, B.; Cornely, O. A.; Wohrmann, A.; Fatkenheuer, G. | no | no | no | yes | Cytomegalovirus | no |
| 23419804 | Comparison of treatment regimens for cytomegalovirus retinitis in patients with AIDS in the era of highly active antiretroviral therapy | Ophthalmology | Jabs, D. A.; Ahuja, A.; Van Natta, M.; Dunn, J. P.; Yeh, S. | no | no | no | yes | Cytomegalovirus | no |
| 25892019 | Long-term Outcomes of Cytomegalovirus Retinitis in the Era of Modern Antiretroviral Therapy: Results from a United States Cohort | Ophthalmology | Jabs, D. A.; Ahuja, A.; Van Natta, M. L.; Lyon, A. T.; Yeh, S.; Danis, R. | no | no | no | no | Cytomegalovirus | yes |
| 22324292 | Glycated Hemoglobin A(1c) as screening for diabetes mellitus in HIV-infected individuals | AIDS Patient Care STDS | Eckhardt, B. J.; Holzman, R. S.; Kwan, C. K.; Baghdadi, J.; Aberg, J. A. | no | no | no | yes | Diabetes | no |
| 23446088 | NGX-4010, a capsaicin 8% dermal patch, for the treatment of painful HIV-associated distal sensory polyneuropathy: results of a 52-week open-label study | Clin J Pain | Simpson, D. M.; Brown, S.; Tobias, J. K.; Vanhove, G. F. | no | no | no | yes | Distal sensory polyneuropathy | no |
| 21851265 | Impact of Tenofovir Versus Abacavir on HIV-Related Endothelial Dysfunction | AIDS Patient Care & STDs | Francisci, Daniela; Falcinelli, Emanuela; Belfiori, Barbara; Petito, Eleonora; Fierro, Tiziana; Baldelli, Franco; Gresele, Paolo | no | no | no | yes | Endothelial Dysfunction | no |
| 21705396 | Acupuncture and the relaxation response for treating gastrointestinal symptoms in HIV patients on highly active antiretroviral therapy | Acupunct Med | Chang, B. H.; Sommers, E. | no | no | no | yes | Gastrointestinal disease | no |
| 25314249 | Association of markers of hemostasis with death in HIV-infected women | J Acquir Immune Defic Syndr | Kiefer, E.; Hoover, D. R.; Shi, Q.; Kuniholm, M. H.; Augenbraun, M.; Cohen, M. H.; Golub, E. T.; Kaplan, R. C.; Liu, C.; Nowicki, M.; Tien, P. C.; Tracy, R. P.; Anastos, K. | no | no | no | no | Hemostasis | yes |
| 105175989. Language: | Hepatitis B virus drug resistance in HIV-1-infected patients taking lamivudine-containing antiretroviral therapy | AIDS Patient Care & STDs | Wongprasit, P.; Manosuthi, W.; Kiertiburanakul, S.; Sungkanuparph, S. | no | no | no | yes | Hepatitis B | yes |
| 24606220 | Use of hepatitis B surface and 'e' antigen quantification during extensive treatment with tenofovir in patients co-infected with HIV-HBV | Liver Int | Boyd, A.; Maylin, S.; Gozlan, J.; Delaugerre, C.; Simon, F.; Girard, P. M.; Lacombe, K. | no | no | no | no | Hepatitis B | yes |
| 24286128 | Survival of HIV-positive individuals with hepatitis B and C infection in Michigan | Epidemiol Infect | Butt, Z. A.; Wilkins, M. J.; Hamilton, E.; Todem, D.; Gardiner, J. C.; Saeed, M. | no | no | no | yes | Hepatitis B, C | yes |
| 15812611 | Drug sharing among heroin networks: implications for HIV and hepatitis B and C prevention | AIDS Behav | Koester, S.; Glanz, J.; Baron, A. | no | no | no | yes | Hepatitis B, C | yes |
| 25065513 | Clinical outcomes of hepatitis B virus coinfection in a United States cohort of hepatitis C virus-infected patients | Hepatology | Kruse, R. L.; Kramer, J. R.; Tyson, G. L.; Duan, Z.; Chen, L.; El-Serag, H. B.; Kanwal, F. | no | no | no | no | Hepatitis B, C | yes |
| 19682317 | Longitudinal evaluation of viral interactions in treated HIV-hepatitis B co-infected patients with additional hepatitis C and D virus | J Viral Hepat | Boyd, A.; Lacombe, K.; Miailhes, P.; Gozlan, J.; Bonnard, P.; Molina, J. M.; Lascoux-Combe, C.; Serfaty, L.; Gault, E.; Desvarieux, M.; Girard, P. M. | no | no | no | no | Hepatitis B, C, D | yes |
| 20205279 | Predictors of loss of hepatitis B surface antigen in HIV-infected patients | World J Gastroenterol | Psevdos, G., Jr.; Kim, J. H.; Suh, J. S.; Sharp, V. L. | no | no | no | yes | Hepatitis C | no |
| 16163639 | Liver injury and changes in hepatitis C Virus (HCV) RNA load associated with protease inhibitor-based antiretroviral therapy for treatment-naive HCV-HIV-coinfected patients: lopinavir-ritonavir versus nelfinavir | Clin Infect Dis | Sherman, K. E.; Shire, N. J.; Cernohous, P.; Rouster, S. D.; Omachi, J. H.; Brun, S.; Da Silva, B. | no | no | no | yes | Hepatitis C | yes |
| 22155913 | Relative impact of ribavirin monitoring and HIV coinfection on sustained virological response in patients with chronic hepatitis C | Antivir Ther | Piedoux, S.; Monnet, E.; Piroth, L.; Montange, D.; Royer, B.; Thevenot, T.; Kantelip, J. P.; Di Martino, V.; Muret, P. | no | no | no | yes | Hepatitis C | yes |
| 21376083 | HCV RNA decline in the first 24 h exhibits high negative predictive value of sustained virologic response in HIV/HCV genotype 1 co-infected patients treated with peginterferon and ribavirin | Antiviral Res | Laufer, N.; Bolcic, F.; Rolon, M. J.; Martinez, A.; Reynoso, R.; Perez, H.; Salomon, H.; Cahn, P.; Quarleri, J. | no | no | no | yes | Hepatitis C | yes |
| 17194480 | Impact of peginterferon alpha-2b and ribavirin treatment on liver tissue in patients with HCV or HCV-HIV co-infection | J Infect | Sarmento-Castro, R.; Horta, A.; Vasconcelos, O.; Coelho, H.; Mendez, J.; Tavares, A. P.; Seabra, J.; Duarte, M.; Chaves, L.; Fortes, O.; Recalde, C.; Ventura, A.; Pires, N.; Pinho, L.; Dias, N.; Carneiro, F. | no | no | no | yes | Hepatitis C | yes |
| 18596682 | Role of pegylated interferon-alpha-2a and ribavirin concentrations in sustained viral response in HCV/HIV-coinfected patients | Clin Pharmacol Ther | Lopez-Cortes, L. F.; Valera-Bestard, B.; Gutierrez-Valencia, A.; Ruiz-Valderas, R.; Jimenez, L.; Arizcorreta, A.; Terron, A.; Viciana, P. | no | no | no | yes | Hepatitis C | yes |
| 18486266 | Sexual transmission is associated with spontaneous HCV clearance in HIV-infected patients | J Hepatol | Shores, N. J.; Maida, I.; Soriano, V.; Nunez, M. | no | no | no | yes | Hepatitis C | yes |
| 20921898 | Sustained long-term antiviral maintenance therapy in HCV/HIV-coinfected patients (SLAM-C) | J Acquir Immune Defic Syndr | Sherman, K. E.; Andersen, J. W.; Butt, A. A.; Umbleja, T.; Alston, B.; Koziel, M. J.; Peters, M. G.; Sulkowski, M.; Goodman, Z. D.; Chung, R. T. | no | no | no | yes | Hepatitis C | yes |
| 105904536. Language: | Predictors of treatment of hepatitis C virus (HCV) infection in drug users | Substance Abuse | Reed, C.; Stuver, S. O.; Tumilty, S.; Nunes, D.; Murray, J. E.; Graham, C. S.; Koziel, M. J.; Craven, D. E.; Skolnik, P. R.; Horsburgh, C. R., Jr. | no | no | no | yes | Hepatitis C | yes |
| 25766991 | TLR3 polymorphisms are associated with virologic response to hepatitis C virus (HCV) treatment in HIV/HCV coinfected patients | J Clin Virol | Torre 3rd, Peter and Hoffman, Howard J. and Springer, Gayle | no | no | no | yes | Hepatitis C | no |
| 21900715 | Effect of an induction period of pegylated interferon-alpha2a and ribavirin on early virological response in HIV-HCV-coinfected patients: results from the CORAL-2 study | Antivir Ther | Tural, C.; Sola, R.; Alvarez, N. P.; Molto, J.; Sanchez, M.; Zamora, A. M.; Ornelas, A.; Laguno, M.; Gonzalez, J.; von Wichmann, M. A.; Tellez, M. J.; Paredes, R.; Clotet, B. | no | no | no | yes | Hepatitis C | no |
| 22592094 | Peginterferon alfa-2a plus ribavirin for HIV-HCV genotype 1 coinfected patients: a randomized international trial | HIV Clin Trials | Rodriguez-Torres, M.; Slim, J.; Bhatti, L.; Sterling, R.; Sulkowski, M.; Hassanein, T.; Serrao, R.; Sola, R.; Bertasso, A.; Passe,; Stancic, S. | no | no | no | yes | Hepatitis C | no |
| 25503739 | Boceprevir and telaprevir-based regimens for the treatment of hepatitis C virus in HIV/HCV coinfected patients | Eur J Gastroenterol Hepatol | Beste, L. A.; Green, P. K.; Ioannou, G. N. | no | no | no | yes | Hepatitis C | no |
| 17659577 | Clinical progression of hepatitis C virus-related chronic liver disease in human immunodeficiency virus-infected patients undergoing highly active antiretroviral therapy | Hepatology | Pineda, J. A.; Garcia-Garcia, J. A.; Aguilar-Guisado, M.; Rios-Villegas, M. J.; Ruiz-Morales, J.; Rivero, A.; del Valle, J.; Luque, R.; Rodriguez-Bano, J.; Gonzalez-Serrano, M.; Camacho, A.; Macias, J.; Grilo, I.; Gomez-Mateos, J. M. | no | no | no | yes | Hepatitis C | no |
| 17349451 | Cost-effectiveness of treatment for hepatitis C in an urban cohort co-infected with HIV | Am J Med | Campos, N. G.; Salomon, J. A.; Servoss, J. C.; Nunes, D. P.; Samet, J. H.; Freedberg, K. A.; Goldie, S. J. | no | no | no | yes | Hepatitis C | no |
| 26196502 | Daclatasvir plus Sofosbuvir for HCV in Patients Coinfected with HIV-1 | N Engl J Med | Wyles, D. L.; Ruane, P. J.; Sulkowski, M. S.; Dieterich, D.; Luetkemeyer, A.; Morgan, T. R.; Sherman, K. E.; Dretler, R.; Fishbein, D.; Gathe, J. C., Jr.; Henn, S.; Hinestrosa, F.; Huynh, C.; McDonald, C.; Mills, A.; Overton, E. T.; Ramgopal, M.; Rashbaum, B.; Ray, G.; Scarsella, A.; Yozviak, J.; McPhee, F.; Liu, Z.; Hughes, E.; Yin, P. D.; Noviello, S.; Ackerman, P. | no | no | no | yes | Hepatitis C | no |
| 25467560 | Efficacy and safety of 8 weeks versus 12 weeks of treatment with grazoprevir (MK-5172) and elbasvir (MK-8742) with or without ribavirin in patients with hepatitis C virus genotype 1 mono-infection and HIV/hepatitis C virus co-infection (C-WORTHY): a randomised, open-label phase 2 trial | Lancet | Sulkowski, M.; Hezode, C.; Gerstoft, J.; Vierling, J. M.; Mallolas, J.; Pol, S.; Kugelmas, M.; Murillo, A.; Weis, N.; Nahass, R.; Shibolet, O.; Serfaty, L.; Bourliere, M.; DeJesus, E.; Zuckerman, E.; Dutko, F.; Shaughnessy, M.; Hwang, P.; Howe, A. Y.; Wahl, J.; Robertson, M.; Barr, E.; Haber, B. | no | no | no | yes | Hepatitis C | no |
| 18854330 | Efficacy of pegylated interferon plus ribavirin treatment in HIV/hepatitis C virus co-infected patients receiving abacavir plus lamivudine or tenofovir plus either lamivudine or emtricitabine as nucleoside analogue backbone | J Antimicrob Chemother | Mira, J. A.; Lopez-Cortes, L. F.; Barreiro, P.; Tural, C.; Torres-Tortosa, M.; de Los Santos Gil, I.; Martin-Rico, P.; Rios-Villegas, M. J.; Hernandez-Burruezo, J. J.; Merino, D.; Lopez-Ruz, M. A.; Rivero, A.; Munoz, L.; Gonzalez-Serrano, M.; Collado, A.; Macias, J.; Viciana, P.; Soriano, V.; Pineda, J. A. | no | no | no | yes | Hepatitis C | no |
| 25032989 | Hepatitis C virus testing in adults living with HIV: a need for improved screening efforts | PLoS One | Yehia, B. R.; Herati, R. S.; Fleishman, J. A.; Gallant, J. E.; Agwu, A. L.; Berry, S. A.; Korthuis, P. T.; Moore, R. D.; Metlay, J. P.; Gebo, K. A. | no | no | no | yes | Hepatitis C | no |
| 24438679 | Impact of the peginterferon-alpha 2a and ribavirin plasma levels on viral kinetics and sustained virological response in genotype 1 HCV/HIV-co-infected patients with the unfavourable non-CC IL28B genotypes | J Viral Hepat | Torres-Cornejo, A.; Ruiz-Valderas, R.; Jimenez-Jimenez, L.; Abad-Molina, C.; Gutierrez-Valencia, A.; Viciana, P.; Lopez-Cortes, L. F. | no | no | no | yes | Hepatitis C | no |
| 25038354 | Sofosbuvir and ribavirin for hepatitis C in patients with HIV coinfection | Jama | Sulkowski, M. S.; Naggie, S.; Lalezari, J.; Fessel, W. J.; Mounzer, K.; Shuhart, M.; Luetkemeyer, A. F.; Asmuth, D.; Gaggar, A.; Ni, L.; Svarovskaia, E.; Brainard, D. M.; Symonds, W. T.; Subramanian, G. M.; McHutchison, J. G.; Rodriguez-Torres, M.; Dieterich, D. | no | no | no | yes | Hepatitis C | no |
| 17561878 | The treatment of chronic hepatitis C in HIV-infected patients: a meta-analysis | HIV Med | Kim, A. I.; Dorn, A.; Bouajram, R.; Saab, S. | no | no | no | yes | Hepatitis C | no |
| 20367803 | Treatment of hepatitis C virus (HCV) infection in patients coinfected with HIV in the HIV Outpatient Study (HOPS), 1999-2007 | J Viral Hepat | Vellozzi, C.; Buchacz, K.; Baker, R.; Spradling, P. R.; Richardson, J.; Moorman, A.; Tedaldi, E.; Durham, M.; Ward, J.; Brooks, J. T. | no | no | no | yes | Hepatitis C | no |
| 25706232 | Virologic response following combined ledipasvir and sofosbuvir administration in patients with HCV genotype 1 and HIV co-infection | Jama | Osinusi, A.; Townsend, K.; Kohli, A.; Nelson, A.; Seamon, C.; Meissner, E. G.; Bon, D.; Silk, R.; Gross, C.; Price, A.; Sajadi, M.; Sidharthan, S.; Sims, Z.; Herrmann, E.; Hogan, J.; Teferi, G.; Talwani, R.; Proschan, M.; Jenkins, V.; Kleiner, D. E.; Wood, B. J.; Subramanian, G. M.; Pang, P. S.; McHutchison, J. G.; Polis, M. A.; Fauci, A. S.; Masur, H.; Kottilil, S. | no | no | no | yes | Hepatitis C | no |
| 20622678 | Women experience higher rates of adverse events during hepatitis C virus therapy in HIV infection: a meta-analysis | J Acquir Immune Defic Syndr | Bhattacharya, D.; Umbleja, T.; Carrat, F.; Chung, R. T.; Peters, M. G.; Torriani, F.; Andersen, J.; Currier, J. S. | no | no | no | yes | Hepatitis C | no |
| 103940993. Language: | Therapeutic Potential of and Treatment with Boceprevir/Telaprevir-Based Triple-Therapy in HIV/Chronic Hepatitis C Co-Infected Patients in a Real-World Setting | AIDS Patient Care & STDs | Mandorfer, Mattias; Payer, Berit A.; Niederecker, Alexander; Lang, Gerold; Aichelburg, Maximilian C.; Strassl, Robert; Boesecke, Christoph; Rieger, Armin; Trauner, Michael; Peck-Radosavljevic, Markus; Reiberger, Thomas | no | no | no | yes | Hepatitis C | no |
| 20163031 | Treatment of chronic hepatitis C in patients with human immunodeficiency virus (HIV) with weekly peginterferon alpha-2b plus ribavirin: a multi-centred Belgian study | Acta Gastroenterol Belg | Michielsen, P.; Bottieau, E.; Van Vlierberghe, H.; Van Marck, E.; Vandemaele, E.; Denys, M.; Brasseur, J. P.; Popan, M. | no | no | no | yes | Hepatitis C | no |
| 21526398 | Comparison of FIB-4 and APRI in HIV-HCV coinfected patients with normal and elevated ALT | Dig Dis Sci | Shah, A. G.; Smith, P. G.; Sterling, R. K. | no | no | no | yes | Hepatitis C | no |
| 24717818 | Rapid hepatitis C testing among persons at increased risk for infection--Wisconsin, 2012-2013 | MMWR Morb Mortal Wkly Rep | Stockman, L. J.; Guilfoye, S. M.; Benoit, A. L.; Vergeront, J. M.; Davis, J. P. | no | no | no | yes | Hepatitis C | yes |
| 25814041 | Adherence to highly active antiretroviral therapy impact on clinical and economic outcomes for Medicaid enrollees with human immunodeficiency virus and hepatitis C coinfection | AIDS Care | Zhang, S. | no | no | yes | yes | Hepatitis C | yes |
| 25706092 | Ombitasvir, paritaprevir co-dosed with ritonavir, dasabuvir, and ribavirin for hepatitis C in patients co-infected with HIV-1: a randomized trial | Jama | Sulkowski, M. S.; Eron, J. J.; Wyles, D.; Trinh, R.; Lalezari, J.; Wang, C.; Slim, J.; Bhatti, L.; Gathe, J.; Ruane, P. J.; Elion, R.; Bredeek, F.; Brennan, R.; Blick, G.; Khatri, A.; Gibbons, K.; Hu, Y. B.; Fredrick, L.; Schnell, G.; Pilot-Matias, T.; Tripathi, R.; Da Silva-Tillmann, B.; McGovern, B.; Campbell, A. L.; Podsadecki, T. | no | no | no | yes | Hepatitis C | no |
| 16251820 | Provider assessment of eligibility for hepatitis C treatment in HIV-infected homeless and marginally housed persons | Aids | Thompson, V. V.; Ragland, K. E.; Hall, C. S.; Morgan, M.; Bangsberg, D. R. | no | no | no | yes | Hepatitis C | no |
| 17166077 | Outcomes of Project Wall Talk: an HIV/AIDS peer education program implemented within the Texas State Prison system | AIDS Educ Prev | Ross, M. W.; Harzke, A. J.; Scott, D. P.; McCann, K.; Kelley, M. | yes | no | no | yes | Hepatitis C | no |
| 22510354 | Extended therapy with pegylated interferon and weight-based ribavirin for HCV-HIV coinfected patients | HIV Clin Trials | Chung, R. T.; Umbleja, T.; Chen, J. Y.; Andersen, J. W.; Butt, A. A.; Sherman, K. E. | no | no | no | yes | Hepatitis C | yes |
| 22471341 | Outcome of HCV/HIV-coinfected liver transplant recipients: a prospective and multicenter cohort study | Am J Transplant | Miro, J. M.; Montejo, M.; Castells, L.; Rafecas, A.; Moreno, S.; Aguero, F.; Abradelo, M.; Miralles, P.; Torre-Cisneros, J.; Pedreira, J. D.; Cordero, E.; de la Rosa, G.; Moyano, B.; Moreno, A.; Perez, I.; Rimola, A. | no | no | no | yes | Hepatitis C | yes |
| 23396734 | Response predictors and clinical benefits of hepatitis C retreatment with pegylated interferon and ribavirin in HIV/HCV coinfection | Ann Hepatol | Peribanez-Gonzalez, M.; da Silva, M. H.; Vilar, F. C.; Seixas-Santos Nastri, A. C.; Ferreira, P. A.; Focaccia, R.; Mendes Correa, M. C. | no | no | no | yes | Hepatitis C | yes |
| 24065317 | A 24-week treatment strategy with pegylated interferon/ribavirin in HIV/hepatitis C virus genotype 3-coinfected patients who achieved a rapid virologic response results in a high sustained virologic response rate | Clin Infect Dis | Rivero-Juarez, A.; Lopez-Cortes, L. F.; Camacho, A.; Mira, J. A.; Tellez, F.; Marquez, M.; Merino, D.; Pineda, J. A.; Rivero, A. | no | no | no | yes | Hepatitis C | no |
| 25041708 | Acoustic Radiation Force Impulse (ARFI) and Transient Elastography (TE) for evaluation of liver fibrosis in HIV-HCV co-infected patients | BMC Infect Dis | Frulio, N.; Trillaud, H.; Perez, P.; Asselineau, J.; Vandenhende, M.; Hessamfar, M.; Bonnet, F.; Maire, F.; Delaune, J.; De Ledinghen, V.; Morlat, P. | no | no | no | yes | Hepatitis C | no |
| 24334180 | Addition of nitazoxanide to PEG-IFN and ribavirin to improve HCV treatment response in HIV-1 and HCV genotype 1 coinfected persons naive to HCV therapy: results of the ACTG A5269 trial | HIV Clin Trials | Amorosa, V. K.; Luetkemeyer, A.; Kang, M.; Johnson, V. A.; Umbleja, T.; Haas, D. W.; Yesmin, S.; Bardin, M. C.; Chung, R. T.; Alston-Smith, B.; Tebas, P.; Peters, M. G. | no | no | no | yes | Hepatitis C | no |
| 26181704 | Brief Report: Reduced Cell-Associated HTLV-2 DNA in Antiretroviral Treated HIV-1-HCV-Coinfected Patients Who Either Received Interferon-alpha/Ribavirin-Based Hepatitis C Therapy or Had Spontaneous HCV RNA Clearance | J Acquir Immune Defic Syndr | Abad-Fernandez, M.; Dronda, F.; Moreno, A.; Casado, J. L.; Perez-Elias, M. J.; Quereda, C.; Moreno, S.; Vallejo, A. | no | no | no | yes | Hepatitis C | no |
| 22734850 | Course of liver fibrosis in HIV-hepatitis C virus-coinfected patients depending on the response to hepatitis C therapy | AIDS Res Hum Retroviruses | Carton, J. A.; Collazos, J.; de la Fuente, B.; Asensi, V. | no | no | no | yes | Hepatitis C | no |
| 17316873 | Effect of hepatitis C virus treatment in fibrosis progression rate (FPR) and time to cirrhosis (TTC) in patients co-infected with human immunodeficiency virus: a paired liver biopsy study | J Hepatol | Rodriguez-Torres, M.; Rodriguez-Orengo, J. F.; Rios-Bedoya, C. F.; Fernandez-Carbia, A.; Marxuach-Cuetara, A. M.; Lopez-Torres, A.; Jimenez-Rivera, J. | no | no | no | yes | Hepatitis C | no |
| 20101190 | HIV/Hepatitis C virus-coinfected virologic responders to pegylated interferon and ribavirin therapy more frequently incur interferon-related adverse events than nonresponders do | J Acquir Immune Defic Syndr | Osinusi, A.; Rasimas, J. J.; Bishop, R.; Proschan, M.; McLaughlin, M.; Murphy, A.; Cortez, K. J.; Polis, M. A.; Masur, H.; Rosenstein, D.; Kottilil, S. | no | no | no | yes | Hepatitis C | no |
| 22563020 | Impact of peginterferon alpha and ribavirin treatment on lipid profiles and insulin resistance in Hepatitis C virus/HIV-coinfected persons: the AIDS Clinical Trials Group A5178 Study | Clin Infect Dis | Butt, A. A.; Umbleja, T.; Andersen, J. W.; Sherman, K. E.; Chung, R. T. | no | no | no | yes | Hepatitis C | no |
| 21781946 | Influence of interferon-based therapy on liver fibrosis progression in HIV/HCV coinfected patients: a retrospective repeated liver biopsy analysis | J Hepatol | Ingiliz, P.; Valantin, M. A.; Preziosi, P.; Finzi, L.; Pais, R.; Fedchuk, L.; Dominguez, S.; Katlama, C.; Poynard, T.; Benhamou, Y. | no | no | no | yes | Hepatitis C | no |
| 15986873 | Pegylated interferon alpha-2a with or without ribavirin in HCV/HIV coinfection: partially blinded, randomized multicenter trial | Dig Dis Sci | Khalili, M.; Bernstein, D.; Lentz, E.; Barylski, C.; Hoffman-Terry, M. | no | no | no | yes | Hepatitis C | no |
| 15688281 | Pilot study of low-dose interleukin-2, pegylated interferon-alpha 2b, and ribavirin for the treatment of hepatitis C virus infection in patients with HIV infection | J Infect Dis | Glesby, M. J.; Bassett, R.; Alston-Smith, B.; Fichtenbaum, C.; Jacobson, E. L.; Brass, C.; Owens, S.; Sulkowski, M.; Race, E. M.; Sherman, K. E. | no | no | no | yes | Hepatitis C | no |
| 16706615 | Poor response to hepatitis C virus (HCV) therapy in HIV- and HCV-coinfected patients is not due to lower adherence to treatment | AIDS Res Hum Retroviruses | Sola, R.; Galeras, J. A.; Montoliu, S.; Tural, C.; Force, L.; Torra, S.; Montull, S.; Castro, E. R.; Coll, S.; Fuster, D.; Barrufet, P.; Sirera, G.; Gimenez, M. D.; Clotet, B.; Planas, R. | no | no | no | yes | Hepatitis C | no |
| 23873217 | The association of cytopenias and weight loss with hepatitis C virus virologic response in HIV/HCV-coinfected patients treated with PEG-IFN and RBV | J Int Assoc Provid AIDS Care | Slim, J.; Mildvan, D.; Han, J.; Korner, E. | no | no | no | yes | Hepatitis C | no |
| 24670522 | The cost-effectiveness of improved hepatitis C virus therapies in HIV/hepatitis C virus coinfected patients | Aids | Linas, B. P.; Barter, D. M.; Leff, J. A.; DiLorenzo, M.; Schackman, B. R.; Horsburgh, C. R.; Assoumou, S. A.; Salomon, J. A.; Weinstein, M. C.; Kim, A. Y.; Freedberg, K. A. | no | no | no | yes | Hepatitis C | no |
| 25036553 | The hepatitis C cascade of care among HIV infected patients: a call to address ongoing barriers to care | PLoS One | Cachay, E. R.; Hill, L.; Wyles, D.; Colwell, B.; Ballard, C.; Torriani, F.; Mathews, W. C. | no | yes | no | yes | Hepatitis C | no |
| 23835502 | The impact of interleukin 28B rs12979860 single nucleotide polymorphism and liver fibrosis stage on response-guided therapy in HIV/HCV-coinfected patients | Aids | Mandorfer, M.; Neukam, K.; Reiberger, T.; Payer, B. A.; Rivero, A.; Puoti, M.; Boesecke, C.; Baumgarten, A.; Grzeszczuk, A.; Zangerle, R.; Meyer-Olson, D.; Rockstroh, J. K.; Trauner, M.; Pineda, J. A.; Peck-Radosavljevic, M. | no | no | no | yes | Hepatitis C | no |
| 20167995 | The influence of abacavir and other antiretroviral agents on virological response to HCV therapy among antiretroviral-treated HIV-infected patients | Antivir Ther | Amorosa, V. K.; Slim, J.; Mounzer, K.; Bruno, C.; Hoffman-Terry, M.; Dorey-Stein, Z.; Ferrara, T.; Kostman, J. R.; Lo Re, V., 3rd | no | no | no | yes | Hepatitis C | no |
| 16970600 | Zidovudine use but not weight-based ribavirin dosing impacts anaemia during HCV treatment in HIV-infected persons | J Viral Hepat | Alvarez, D.; Dieterich, D. T.; Brau, N.; Moorehead, L.; Ball, L.; Sulkowski, M. S. | no | no | no | yes | Hepatitis C | no |
| 25791727 | A National Study of Outcomes among HIV-Infected Kidney Transplant Recipients | J Am Soc Nephrol | Locke, J. E. | no | no | no | no | Hepatitis C | yes |
| 20151840 | Activation of CD8 T cells predicts progression of HIV infection in women coinfected with hepatitis C virus | J Infect Dis | Kovacs, A.; Karim, R.; Mack, W. J.; Xu, J.; Chen, Z.; Operskalski, E.; Frederick, T.; Landay, A.; Voris, J.; Spencer, L. S.; Young, M. A.; Tien, P. C.; Augenbraun, M.; Strickler, H. D.; Al-Harthi, L. | no | no | no | no | Hepatitis C | yes |
| 19013829 | Comorbidities and their impact on mortality in HCV and HCV-HIV-coinfected persons on dialysis | J Clin Gastroenterol | Butt, A. A.; Khan, U. A.; Skanderson, M. | no | no | no | no | Hepatitis C | yes |
| 22534149 | Mortality in hepatitis C virus-infected patients with a diagnosis of AIDS in the era of combination antiretroviral therapy | Clin Infect Dis | Branch, A. D.; Van Natta, M. L.; Vachon, M. L.; Dieterich, D. T.; Meinert, C. L.; Jabs, D. A. | no | no | no | no | Hepatitis C | yes |
| 22328294 | Outcomes of liver transplant recipients with hepatitis C and human immunodeficiency virus coinfection | Liver Transpl | Terrault, N. A.; Roland, M. E.; Schiano, T.; Dove, L.; Wong, M. T.; Poordad, F.; Ragni, M. V.; Barin, B.; Simon, D.; Olthoff, K. M.; Johnson, L.; Stosor, V.; Jayaweera, D.; Fung, J.; Sherman, K. E.; Subramanian, A.; Millis, J. M.; Slakey, D.; Berg, C. L.; Carlson, L.; Ferrell, L.; Stablein, D. M.; Odim, J.; Fox, L.; Stock, P. G. | no | no | no | no | Hepatitis C | yes |
| 15851720 | Hepatitis C augments cognitive deficits associated with HIV infection and methamphetamine | Neurology | Cherner, M.; Letendre, S.; Heaton, R. K.; Durelle, J.; Marquie-Beck, J.; Gragg, B.; Grant, I. | no | no | no | no | Hepatitis C | yes |
| 20458071 | Hepatitis C seropositivity is not a risk factor for sensory neuropathy among patients with HIV | Neurology | Cherry, C. L.; Affandi, J. S.; Brew, B. J.; Creighton, J.; Djauzi, S.; Hooker, D. J.; Imran, D.; Kamarulzaman, A.; Kamerman, P.; McArthur, J. C.; Moore, R. D.; Price, P.; Smyth, K.; Tan, I. L.; Vanar, S.; Wadley, A.; Wesselingh, S. L.; Yunihastuti, E. | no | no | no | no | Hepatitis C | yes |
| 24127691 | Rates and predictors of response to anti-viral treatment for hepatitis C virus in HIV/HCV co-infection in a nationwide study of 619 patients | Aliment Pharmacol Ther | Ioannou, G. N.; Scott, J. D.; Yang, Y.; Green, P. K.; Beste, L. A. | no | no | no | no | Hepatitis C | yes |
| 19842982 | Meta-analysis: increased mortality associated with hepatitis C in HIV-infected persons is unrelated to HIV disease progression | Clin Infect Dis | Chen, T. Y.; Ding, E. L.; Seage Iii, G. R.; Kim, A. Y. | no | no | no | no | Hepatitis C | yes |
| 24112091 | Chronic hepatitis C virus infection is associated with all-cause and liver-related mortality in a cohort of HIV-infected patients with alcohol problems | Addiction | Fuster, D.; Cheng, D. M.; Quinn, E. K.; Nunes, D.; Saitz, R.; Samet, J. H.; Tsui, J. I. | no | no | no | no | Hepatitis C | yes |
| 16044016 | Effects of hepatitis C virus coinfection on survival in veterans with HIV treated with highly active antiretroviral therapy | J Acquir Immune Defic Syndr | Backus, L. I.; Phillips, B. R.; Boothroyd, D. B.; Mole, L. A.; Burgess, J.; Rigsby, M. O.; Chang, S. W. | no | no | no | no | Hepatitis C | yes |
| 20946441 | Evaluation of the possible influence of hepatitis C virus and liver fibrosis on HIV type 1 immunological and virological outcomes | HIV Med | Collazos, J.; Carton, J. A.; Asensi, V. | no | no | no | no | Hepatitis C | yes |
| 23921611 | Hepatitis C virus/HIV coinfection and responses to initial antiretroviral treatment | Aids | Hua, L.; Andersen, J. W.; Daar, E. S.; Glesby, M. J.; Hollabaugh, K.; Tierney, C. | no | no | no | no | Hepatitis C | yes |
| 19609624 | Race/ethnicity and risk of AIDS and death among HIV-infected patients with access to care | J Gen Intern Med | Silverberg, M. J.; Leyden, W.; Quesenberry, C. P., Jr.; Horberg, M. A. | no | no | no | no | Hepatitis C, mental health | yes |
| 25848927 | Injection Drug Use and Hepatitis C as Risk Factors for Mortality in HIV-Infected Individuals: The Antiretroviral Therapy Cohort Collaboration | J Acquir Immune Defic Syndr | May, M. T.; Justice, A. C.; Birnie, K.; Ingle, S. M.; Smit, C.; Smith, C.; Neau, D.; Guiguet, M.; Schwarze-Zander, C.; Moreno, S.; Guest, J. L.; Monforte, Ad; Tural, C.; Gill, M. J.; Bregenzer, A.; Kirk, O.; Saag, M.; Sterling, T. R.; Crane, H. M.; Sterne, J. A. | no | no | no | no | Hepatitis C, substance abuse | yes |
| 20638118 | Death rates in HIV-positive antiretroviral-naive patients with CD4 count greater than 350 cells per microL in Europe and North America: a pooled cohort observational study | Lancet | Lodwick, R. K.; Sabin, C. A.; Porter, K.; Ledergerber, B.; van Sighem, A.; Cozzi-Lepri, A.; Khaykin, P.; Mocroft, A.; Jacobson, L.; De Wit, S.; Obel, N.; Castagna, A.; Wasmuth, J. C.; Gill, J.; Klein, M. B.; Gange, S.; Riera, M.; Mussini, C.; Gutierrez, F.; Touloumi, G.; Carrieri, P.; Guest, J. L.; Brockmeyer, N. H.; Phillips, A. N. | no | no | no | no | Hepatitis C, substance abuse | yes |
| 16254546 | The impact of highly active antiretroviral therapy and immunodeficiency on human papillomavirus infection of the oral cavity of human immunodeficiency virus-seropositive adults | Sex Transm Dis | Cameron, J. E.; Mercante, D.; O'Brien, M.; Gaffga, A. M.; Leigh, J. E.; Fidel, P. L., Jr.; Hagensee, M. E. | no | no | no | yes | Human papillomavirus | no |
| 17148967 | Effects of metformin and rosiglitazone in HIV-infected patients with hyperinsulinemia and elevated waist/hip ratio | Aids | Mulligan, K.; Yang, Y.; Wininger, D. A.; Koletar, S. L.; Parker, R. A.; Alston-Smith, B. L.; Schouten, J. T.; Fielding, R. A.; Basar, M. T.; Grinspoon, S. | no | no | no | yes | Hyperinsulinemia | no |
| 23277897 | Improved immunogenicity with high-dose seasonal influenza vaccine in HIV-infected persons: a single-center, parallel, randomized trial | Ann Intern Med | McKittrick, N.; Frank, I.; Jacobson, J. M.; White, C. J.; Kim, D.; Kappes, R.; DiGiorgio, C.; Kenney, T.; Boyer, J.; Tebas, P. | no | no | no | yes | Influenza | yes |
| 23947744 | Campath induction in HCV and HCV/HIV-seropositive kidney transplant recipients | Transpl Int | Vivanco, M.; Friedmann, P.; Xia, Y.; Klair, T.; Marfo, K.; de Boccardo, G.; Greenstein, S.; Chapochnick-Friedmann, J.; Kinkhabwala, M.; Ajaimy, M.; Lubetzky, M. L.; Akalin, E.; Kayler, L. K. | no | no | yes | yes | Kidney disease | yes |
| 25015912 | Rosuvastatin preserves renal function and lowers cystatin C in HIV-infected subjects on antiretroviral therapy: the SATURN-HIV trial | Clin Infect Dis | Longenecker, C. T.; Hileman, C. O.; Funderburg, N. T.; McComsey, G. A. | no | no | no | yes | Kidney disease | no |
| 18176076 | Observations on a cohort of HIV-infected patients undergoing native renal biopsy | Am J Nephrol | Berliner, A. R.; Fine, D. M.; Lucas, G. M.; Rahman, M. H.; Racusen, L. C.; Scheel, P. J.; Atta, M. G. | no | no | no | yes | Kidney disease | yes |
| 21372250 | Kidney tubular damage in the absence of glomerular defects in HIV-infected patients on highly active antiretroviral therapy | Nephrol Dial Transplant | Ando, M.; Yanagisawa, N.; Ajisawa, A.; Tsuchiya, K.; Nitta, K. | no | no | no | yes | Kidney disease | no |
| 16686310 | Survival experience of peritoneal dialysis patients with human immunodeficiency virus: a 17-year retrospective study | Adv Perit Dial | Khanna, R.; Tachopoulou, O. A.; Fein, P. A.; Chattopadhyay, J.; Avram, M. M. | no | no | no | no | Kidney disease | yes |
| 22592587 | Comorbid diabetes and the risk of progressive chronic kidney disease in HIV-infected adults: data from the Veterans Aging Cohort Study | J Acquir Immune Defic Syndr | Medapalli, R. K.; Parikh, C. R.; Gordon, K.; Brown, S. T.; Butt, A. A.; Gibert, C. L.; Rimland, D.; Rodriguez-Barradas, M. C.; Chang, C. C.; Justice, A. C.; He, J. C.; Wyatt, C. M. | no | no | no | yes | Kidney disease | yes |
| 20521408 | The morbidity and mortality associated with kidney disease in an HIV-infected cohort in Puerto Rico | Ethn Dis | Mayor, A. M.; Dworkin, M.; Quesada, L.; Rios-Olivares, E.; Hunter-Mellado, R. F. | no | no | no | no | Kidney disease | yes |
| 19133752 | Assessment of ultrasound for use in detecting lipoatrophy in HIV-infected patients taking combination antiretroviral therapy | AIDS Patient Care STDS | Viskovic, K.; Richman, I.; Klasnic, K.; Hernandez, A.; Krolo, I.; Rutherford, G. W.; Romih, V.; Begovac, J. | no | no | no | yes | Lipoatrophy | no |
| 17192148 | Reconstructive treatment for antiretroviral-associated facial lipoatrophy: a prospective study comparing autologous fat and synthetic substances | AIDS Patient Care STDS | Negredo, E.; Higueras, C.; Adell, X.; Martinez, J. C.; Martinez, E.; Puig, J.; Fumaz, C. R.; Munoz-Moreno, J. A.; Perez-Alvarez, N.; Videla, S.; Estany, C.; Cinquegrana, D.; Gonzalez-Mestre, V.; Clotet, B. | no | no | no | yes | Lipoatrophy | no |
| 109797635. Language: | RESEARCH AND PRACTICE. A Comparison of Liver Disease Mortality With HIV and Overdose Mortality Among Georgia Prisoners and Releasees: A 2-Decade Cohort Study of Prisoners Incarcerated in 1991 | American Journal of Public Health | Spaulding, Anne C.; Sharma, Akshay; Messina, Lauren C.; Zlotorynska, Maria; Miller, Lesley; Binswanger, Ingrid | no | no | no | yes | Liver disease | yes |
| 22820790 | Relationship of liver disease stage and antiviral therapy with liver-related events and death in adults coinfected with HIV/HCV | Jama | Limketkai, B. N.; Mehta, S. H.; Sutcliffe, C. G.; Higgins, Y. M.; Torbenson, M. S.; Brinkley, S. C.; Moore, R. D.; Thomas, D. L.; Sulkowski, M. S. | no | no | no | no | Liver disease | yes |
| 25977266 | High Cure Rate With 24 Weeks of Daclatasvir-Based Quadruple Therapy in Treatment-Experienced, Null-Responder Patients With HIV/Hepatitis C Virus Genotype 1/4 Coinfection: The ANRS HC30 QUADRIH Study | Clin Infect Dis | Piroth, L. | no | no | no | yes | Liver disease | no |
| 22698023 | Gender abuse, depressive symptoms, and HIV and other sexually transmitted infections among male-to-female transgender persons: a three-year prospective study | Am J Public Health | Nuttbrock, L.; Bockting, W.; Rosenblum, A.; Hwahng, S.; Mason, M.; Macri, M.; Becker, J. | no | no | no | yes | Mental health | yes |
| 19722288 | Screening for major depression in persons with HIV infection: the concurrent predictive validity of the Profile of Mood States Depression-Dejection Scale | Int J Methods Psychiatr Res | Patterson, K.; Young, C.; Woods, S. P.; Vigil, O.; Grant, I.; Atkinson, J. H. | no | no | no | yes | Mental health | yes |
| 16338766 | Effects of treated and untreated depressive symptoms on highly active antiretroviral therapy use in a US multi-site cohort of HIV-positive women | AIDS Care | Cook, J. A.; Grey, D.; Burke-Miller, J.; Cohen, M. H.; Anastos, K.; Gandhi, M.; Richardson, J.; Wilson, T.; Young, M. | no | yes | no | yes | Mental health | no |
| 16390890 | Placebo-controlled trial of dehydroepiandrosterone (DHEA) for treatment of nonmajor depression in patients with HIV/AIDS | Am J Psychiatry | Rabkin, J. G.; McElhiney, M. C.; Rabkin, R.; McGrath, P. J.; Ferrando, S. J. | no | no | no | yes | Mental health | no |
| 19753412 | A brief survey of awareness of common health conditions, access to health services, and utilization of health services in limited-English-proficiency Hispanic/Latino adults | J Allied Health | Wolff, J.; Ellis, C. | no | no | no | yes | Mental health | no |
| 50791842 | Psychosocial Stressors of Families Affected by HIV/AIDS: Implications for Social Work Practice | Journal of HIV/AIDS & Social Services | Lichtenstein, Bronwen; Sturdevant, Marsha S.; Mujumdar, Anil A. | no | no | no | yes | Mental health | yes |
| 104991042. Language: | Remote semantic memory for public figures in hiv infection, alcoholism, and their comorbidity | Alcoholism: Clinical & Experimental Research | Fama, R. | no | no | no | yes | Mental health | yes |
| 22292903 | Attachment, forgiveness, and physical health quality of life in HIV + adults | AIDS Care | Martin, L. A.; Vosvick, M.; Riggs, S. A. | no | no | no | yes | Mental health | yes |
| 18154491 | Major depression in patients with HIV/AIDS and substance abuse | AIDS Patient Care STDS | Berger-Greenstein, J. A.; Cuevas, C. A.; Brady, S. M.; Trezza, G.; Richardson, M. A.; Keane, T. M. | no | no | no | yes | Mental health | yes |
| 23086427 | Routine depression screening in an HIV clinic cohort identifies patients with complex psychiatric co-morbidities who show significant response to treatment | AIDS Behav | Schumacher, J. E.; McCullumsmith, C.; Mugavero, M. J.; Ingle-Pang, P. E.; Raper, J. L.; Willig, J. H.; You, Z.; Batey, D. S.; Crane, H.; Lawrence, S. T.; Wright, C.; Treisman, G.; Saag, M. S. | no | no | no | yes | Mental health | yes |
| 2006-07312-002 | Predictors of quality of life in HIV-infected rural women: Psychometric test of the chronic illness quality of life ladder | Quality of Life Research: An International Journal of Quality of Life Aspects of Treatment, Care & Rehabilitation | Murdaugh, Carolyn; Moneyham, Linda; Jackson, Kirby; Phillips, Kenneth; Tavakoli, Abbas | no | no | no | yes | Mental health | yes |
| 16782673 | Social support and depressive symptomatology among HIV-positive women: the mediating role of self-esteem and mastery | Women Health | Simoni, J. M.; Montoya, H. D.; Huang, B.; Goodry, E. | no | no | no | yes | Mental health | no |
| 24469525 | Escitalopram treatment of depression in human immunodeficiency virus/acquired immunodeficiency syndrome: a randomized, double-blind, placebo-controlled study | Journal of Nervous & Mental Disease | Hoare, Jacqueline; Carey, Paul; Joska, John A.; Carrara, Henri; Sorsdahl, Katherine; Stein, Dan J. | no | no | no | yes | Mental health | no |
| 104530125. Language: | Medical Outcomes of HIV-Infected Individuals Two Years After Initiating Specialized Psychiatric Treatment Services | Journal of HIV/AIDS & Social Services | Brown, Jennifer L.; Stepleman, Lara M.; Bottonari, Kathryn A. | no | no | no | yes | Mental health | no |
| 16831788 | Strategies for self-management of HIV-related anxiety | AIDS Care | Kemppainen, J. K.; Eller, L. S.; Bunch, E.; Hamilton, M. J.; Dole, P.; Holzemer, W.; Kirksey, K.; Nicholas, P. K.; Corless, I. B.; Coleman, C.; Nokes, K. M.; Reynolds, N.; Sefcik, L.; Wantland, D.; Tsai, Y. F. | no | no | no | yes | Mental health | no |
| 19387832 | Effects of antidepressant treatment on antiretroviral regimen adherence among depressed HIV-infected patients | Psychiatr Q | Kumar, V.; Encinosa, W. | no | yes | no | yes | Mental health | no |
| 105466857. Language: | Factors associated with psychotherapy longevity among HIV-positive patients | AIDS Patient Care & STDs | Bottonari, K. A.; Stepleman, L. M. | no | yes | no | yes | Mental health | no |
| 24313252 | Social care networks and older LGBT adults: challenges for the future | J Homosex | Brennan-Ing, M.; Seidel, L.; Larson, B.; Karpiak, S. E. | yes | yes | yes | yes | Mental health | no |
| 106371089. Language: | A comparison study of homeless and non-homeless HIV-positive persons enrolled in mental health care | Journal of HIV/AIDS & Social Services | Smith, B. D.; DeWeaver, K. L. | yes | no | yes | yes | Mental health | no |
| 19892212 | Use of outpatient mental health services and psychotropic medications among HIV-infected patients in a multisite, multistate study | General Hospital Psychiatry | Himelhoch, S. | yes | no | no | yes | Mental health | no |
| 22085330 | HIV risk among female sex workers in Miami: the impact of violent victimization and untreated mental illness | AIDS Care | Surratt, H. L.; Kurtz, S. P.; Chen, M.; Mooss, A. | no | no | no | yes | Mental health | yes |
| 18001228 | Utility of the Millon Behavioral Medicine Diagnostic (MBMD) to predict adherence to highly active antiretroviral therapy (HAART) medication regimens among HIV-positive men and women | J Pers Assess | Cruess, D. G.; Minor, S.; Antoni, M. H.; Millon, T. | no | no | no | yes | Mental health | yes |
| 18095166 | Antidepressant treatment and adherence to combination antiretroviral therapy among patients with AIDS and diagnosed depression | Psychiatr Q | Walkup, J.; Wei, W.; Sambamoorthi, U.; Crystal, S. | no | no | yes | yes | Mental health | no |
| 24668254 | Changes in sexual behavior of HIV-infected older adults enrolled in a clinical trial of standalone group psychotherapies targeting depression | AIDS Behav | Lovejoy, T. I.; Heckman, T. G.; Sikkema, K. J.; Hansen, N. B.; Kochman, A. | no | no | no | yes | Mental health | no |
| 22296865 | DHEA and cognition in HIV-positive patients with non-major depression | Psychosomatics | Bradley, M.; McElhiney, M.; Rabkin, J. | no | no | no | yes | Mental health | no |
| 19282781 | Psychological well-being among individuals aging with HIV: the value of social relationships | J Acquir Immune Defic Syndr | Mavandadi, S.; Zanjani, F.; Ten Have, T. R.; Oslin, D. W. | no | no | no | yes | Mental health | no |
| 23715264 | The effect of relaxation interventions on cortisol levels in HIV-seropositive women | J Int Assoc Provid AIDS Care | Jones, D.; Owens, M.; Kumar, M.; Cook, R.; Weiss, S. M. | no | no | no | yes | Mental health | no |
| 21667297 | The effects of traumatic stressors and HIV-related trauma symptoms on health and health related quality of life | AIDS Behav | Nightingale, V. R.; Sher, T. G.; Mattson, M.; Thilges, S.; Hansen, N. B. | no | no | no | yes | Mental health | no |
| 22545737 | Cognitive behavioral therapy for adherence and depression (CBT-AD) in HIV-infected injection drug users: a randomized controlled trial | J Consult Clin Psychol | Safren, S. A.; O'Cleirigh, C. M.; Bullis, J. R.; Otto, M. W.; Stein, M. D.; Pollack, M. H. | yes | no | no | yes | Mental health | no |
| 23244367 | Gender-specific effects of an augmented written emotional disclosure intervention on posttraumatic, depressive, and HIV-disease-related outcomes: a randomized, controlled trial | J Consult Clin Psychol | Ironson, G.; O'Cleirigh, C.; Leserman, J.; Stuetzle, R.; Fordiani, J.; Fletcher, M.; Schneiderman, N. | yes | no | no | yes | Mental health | no |
| 23050767 | In-home mental health treatment for individuals with HIV | AIDS Patient Care STDS | Reif, S. S.; Pence, B. W.; LeGrand, S.; Wilson, E. S.; Swartz, M.; Ellington, T.; Whetten, K. | yes | yes | no | yes | Mental health | no |
| 20845112 | Self-efficacy and distress in women with AIDS: the SMART/EST women's project | AIDS Care | Jones, D. L.; Ishii Owens, M.; Lydston, D.; Tobin, J. N.; Brondolo, E.; Weiss, S. M. | yes | no | no | yes | Mental health | no |
| 23644816 | Telephone based cognitive behavioral therapy targeting major depression among urban dwelling, low income people living with HIV/AIDS: results of a randomized controlled trial | AIDS Behav | Himelhoch, S.; Medoff, D.; Maxfield, J.; Dihmes, S.; Dixon, L.; Robinson, C.; Potts, W.; Mohr, D. C. | yes | no | no | yes | Mental health | no |
| 24400124 | The effects of a problem solving-based intervention on depressive symptoms and HIV medication adherence are independent | PLoS One | Gross, R.; Bellamy, S. L.; Chapman, J.; Han, X.; O'Duor, J.; Strom, B. L.; Houts, P. S.; Palmer, S. C.; Coyne, J. C. | yes | no | no | yes | Mental health | no |
| 17502728 | The effect of adherence on the association between depressive symptoms and mortality among HIV-infected individuals first initiating HAART | Aids | Lima, V. D.; Geller, J.; Bangsberg, D. R.; Patterson, T. L.; Daniel, M.; Kerr, T.; Montaner, J.; Hogg, R. S. | no | no | no | no | Mental health | yes |
| 103771485. Language: | Can behavioral theory inform the understanding of depression and medication nonadherence among HIV-positive substance users? | Journal of Behavioral Medicine | Magidson, Jessica; Listhaus, Alyson; Seitz-Brown, C.; Safren, Steven; Lejuez, C.; Daughters, Stacey | no | no | no | no | Mental health | yes |
| 23768670 | Differential associations between perceived and objective measurement of distress tolerance in relation to antiretroviral treatment adherence and response among HIV-positive individuals | Behav Ther | Oser, M. L.; Trafton, J. A.; Lejuez, C. W.; Bonn-Miller, M. O. | no | no | no | no | Mental health | yes |
| 23975476 | Does effective depression treatment alone reduce secondary HIV transmission risk? Equivocal findings from a randomized controlled trial | AIDS Behav | Tsai, A. C.; Mimiaga, M. J.; Dilley, J. W.; Hammer, G. P.; Karasic, D. H.; Charlebois, E. D.; Sorensen, J. L.; Safren, S. A.; Bangsberg, D. R. | no | no | no | no | Mental health | yes |
| 21784531 | Lifetime suicidal ideation and attempt are common among HIV+ individuals | J Affect Disord | Badiee, J.; Moore, D. J.; Atkinson, J. H.; Vaida, F.; Gerard, M.; Duarte, N. A.; Franklin, D.; Gouaux, B.; McCutchan, J. A.; Heaton, R. K.; McArthur, J.; Morgello, S.; Simpson, D.; Collier, A.; Marra, C. M.; Gelman, B.; Clifford, D.; Grant, I. | no | no | no | no | Mental health | yes |
| 21116186 | Psychiatric risk factors for HIV disease progression: the role of inconsistent patterns of antiretroviral therapy utilization | J Acquir Immune Defic Syndr | Carrico, A. W.; Riley, E. D.; Johnson, M. O.; Charlebois, E. D.; Neilands, T. B.; Remien, R. H.; Lightfoot, M. A.; Steward, W. T.; Weinhardt, L. S.; Kelly, J. A.; Rotheram-Borus, M. J.; Morin, S. F.; Chesney, M. A. | no | no | no | no | Mental health | yes |
| 22012149 | The impact of prolonged exposure on PTSD symptoms and associated psychopathology in people living with HIV: a randomized test of concept | AIDS Behav | Pacella, M. L.; Armelie, A.; Boarts, J.; Wagner, G.; Jones, T.; Feeny, N.; Delahanty, D. L. | no | no | no | no | Mental health | yes |
| 105738567. Language: | The relationship of post-traumatic stress disorder and depression to antiretroviral medication adherence in persons with HIV | AIDS Patient Care & STDs | Vranceanu, A. M.; Safren, S. A.; Lu, M.; Coady, W. M.; Skolnik, P. R.; Rogers, W. H.; Wilson, I. B. | no | no | no | no | Mental health | yes |
| 21399478 | Psychiatric correlates of HAART utilization and viral load among HIV-positive impoverished persons | Aids | Carrico, A. W.; Bangsberg, D. R.; Weiser, S. D.; Chartier, M.; Dilworth, S. E.; Riley, E. D. | no | no | no | no | Mental health | yes |
| 103768061. Language: | Implementing an Intimate Partner Violence (IPV) Screening Protocol in HIV Care | AIDS Patient Care & STDs | Raissi, Sadaf E.; Krentz, Hartmut B.; Siemieniuk, Reed A. C.; Gill, M. John | yes | no | no | no | Mental health | yes |
| 16954726 | Psychological resources protect health: 5-year survival and immune function among HIV-infected women from four US cities | Aids | Ickovics, J. R.; Milan, S.; Boland, R.; Schoenbaum, E.; Schuman, P.; Vlahov, D. | yes | no | no | no | Mental health | yes |
| 25081100 | The interaction of active substance use, depression, and antiretroviral adherence in methadone maintenance | Int J Behav Med | Newville, H.; Berg, K. M.; Gonzalez, J. S. | no | no | no | yes | Mental health, substance abuse | no |
| 17019897 | Interrater reliability of the Psychiatric Research Interview for Substance and Mental Disorders in an HIV-infected cohort: experience of the National NeuroAIDS Tissue Consortium | Int J Methods Psychiatr Res | Morgello, S.; Holzer, C. E., 3rd; Ryan, E.; Young, C.; Naseer, M.; Castellon, S. A.; Frol, A. B.; Atkinson, J. H.; Gelman, B. B.; Grant, I.; Singer, E. J. | no | no | no | yes | Mental health, substance abuse | no |
| 20969465 | Mortality after diagnosis of psychiatric disorders and co-occurring substance use disorders among HIV-infected patients | AIDS Patient Care STDS | DeLorenze, G. N.; Satre, D. D.; Quesenberry, C. P.; Tsai, A. L.; Weisner, C. M. | no | no | no | no | Mental health, substance abuse | yes |
| 19008505 | Substance abuse and hospitalization for mood disorder among Medicaid beneficiaries | Am J Public Health | Prince, J. D.; Akincigil, A.; Hoover, D. R.; Walkup, J. T.; Bilder, S.; Crystal, S. | no | no | no | no | Mental health, substance abuse | yes |
| 105608814. Language: | Gender and other psychosocial factors as predictors of adherence to highly active antiretroviral therapy (HAART) in adults with comorbid HIV/AIDS, psychiatric and substance-related disorder | AIDS & Behavior | Applebaum, A. J. and Richardson, M. A. and Brady, S. M. and Brief, D. J. and Keane, T. M. | no | no | no | yes | Mental health, substance abuse | yes |
| 16938671 | Improving health outcomes among individuals with HIV, mental illness, and substance use disorders in the Southeast | AIDS Care | Whetten, K.; Reif, S.; Ostermann, J.; Pence, B. W.; Swartz, M.; Whetten, R.; Conover, C.; Bouis, S.; Thielman, N.; Eron, J. | yes | no | yes | yes | Mental health, substance abuse | no |
| 22115794 | Pain and physical and psychological symptoms in ambulatory HIV patients in the current treatment era | J Pain Symptom Manage | Merlin, J. S.; Cen, L.; Praestgaard, A.; Turner, M.; Obando, A.; Alpert, C.; Woolston, S.; Casarett, D.; Kostman, J.; Gross, R.; Frank, I. | no | no | no | yes | Mental health, substance abuse | no |
| 19537957 | Medication adherence in HIV-infected smokers: the mediating role of depressive symptoms | AIDS Educ Prev | Webb, M. S.; Vanable, P. A.; Carey, M. P.; Blair, D. C. | no | no | no | yes | Mental health, substance abuse | yes |
| 17919095 | Predictors of AIDS-related morbidity and mortality in a southern U.S. Cohort | AIDS Patient Care STDS | Mugavero, M. J.; Pence, B. W.; Whetten, K.; Leserman, J.; Swartz, M.; Stangl, D.; Thielman, N. M. | no | no | no | no | Mental health, substance abuse | yes |
| 22042879 | Patient reported outcomes in routine care: advancing data capture for HIV cohort research | Clin Infect Dis | Kozak, M. S.; Mugavero, M. J.; Ye, J.; Aban, I.; Lawrence, S. T.; Nevin, C. R.; Raper, J. L.; McCullumsmith, C.; Schumacher, J. E.; Crane, H. M.; Kitahata, M. M.; Saag, M. S.; Willig, J. H. | no | no | no | no | Mental health, substance abuse | yes |
| 20098331 | Microalbuminuria is associated with all-cause and AIDS mortality in women with HIV infection | J Acquir Immune Defic Syndr | Wyatt, C. M.; Hoover, D. R.; Shi, Q.; Seaberg, E.; Wei, C.; Tien, P. C.; Karim, R.; Lazar, J.; Young, M. A.; Cohen, M. H.; Klotman, P. E.; Anastos, K. | no | no | no | no | Microalbuminuria | yes |
| 21685547 | Pre-existing albuminuria predicts AIDS and non-AIDS mortality in women initiating antiretroviral therapy | Antivir Ther | Wyatt, C. M.; Hoover, D. R.; Shi, Q.; Tien, P. C.; Karim, R.; Cohen, M. H.; Goderre, J. L.; Seaberg, E. C.; Lazar, J.; Young, M. A.; Klotman, P. E.; Anastos, K. | no | no | no | no | Microalbuminuria | yes |
| 23459863 | HIV infection and the risk of acute myocardial infarction | JAMA Intern Med | Freiberg, M. S.; Chang, C. C.; Kuller, L. H.; Skanderson, M.; Lowy, E.; Kraemer, K. L.; Butt, A. A.; Bidwell Goetz, M.; Leaf, D.; Oursler, K. A.; Rimland, D.; Rodriguez Barradas, M.; Brown, S.; Gibert, C.; McGinnis, K.; Crothers, K.; Sico, J.; Crane, H.; Warner, A.; Gottlieb, S.; Gottdiener, J.; Tracy, R. P.; Budoff, M.; Watson, C.; Armah, K. A.; Doebler, D.; Bryant, K.; Justice, A. C. | no | no | no | no | Multiple | yes |
| 24580808 | Therapy duration and long-term outcomes in extra-pulmonary tuberculosis | BMC Infect Dis | Pusch, T.; Pasipanodya, J. G.; Hall, R. G., 2nd; Gumbo, T. | no | no | no | yes | Multiple | yes |
| 26125093 | Evaluating the Effects of an Interdisciplinary Practice Model with Pharmacist Collaboration on HIV Patient Co-Morbidities | AIDS Patient Care STDS | Cope, R. | yes | no | no | yes | Multiple | no |
| 18681098 | Differences in HIV-related hospitalization among white, black, and Hispanic men and women of Florida | Women Health | Hlaing, W. M.; McCoy, H. V. | no | yes | no | yes | Multiple | no |
| 109820935. Language: | Evaluating the Effects of an Interdisciplinary Practice Model with Pharmacist Collaboration on HIV Patient Co-Morbidities | AIDS Patient Care & STDs | Cope, Rebecca; Berkowitz, Leonard; Arcebido, Rebecca; Yeh, Jun-Yen; Trustman, Nathan; Cha, Agnes | yes | no | no | yes | Multiple | no |
| 17039981 | Predictors of death within six months in patients with advanced AIDS | Palliat Support Care | Brechtl, J. R.; Patrick, P. A.; Visintainer, P.; Brand, D. A. | no | no | no | no | Multiple | yes |
| 16311491 | The effect of socioeconomic status on the survival of people receiving care for HIV infection in the United States | J Health Care Poor Underserved | Cunningham, W. E.; Hays, R. D.; Duan, N.; Andersen, R.; Nakazono, T. T.; Bozzette, S. A.; Shapiro, M. F. | no | no | yes | no | Multiple | yes |
| 21146004 | CD4 count is predictive of outcome in HIV-positive patients undergoing abdominal operations | Am J Surg | Deneve, J. L.; Shantha, J. G.; Page, A. J.; Wyrzykowski, A. D.; Rozycki, G. S.; Feliciano, D. V. | no | no | no | no | Multiple | yes |
| 25202921 | An adapted frailty-related phenotype and the VACS index as predictors of hospitalization and mortality in HIV-infected and uninfected individuals | J Acquir Immune Defic Syndr | Akgun, K. M.; Tate, J. P.; Crothers, K.; Crystal, S.; Leaf, D. A.; Womack, J.; Brown, T. T.; Justice, A. C.; Oursler, K. K. | no | no | no | no | Multiple | yes |
| 23080359 | Antiretroviral adherence among rural compared to urban veterans with HIV infection in the United States | AIDS Behav | Ohl, M. E.; Perencevich, E.; McInnes, D. K.; Kim, N.; Rimland, D.; Akgun, K.; Fiellin, D. A.; Skanderson, M.; Wang, K.; Justice, A. | no | no | no | no | Multiple | yes |
| 23921612 | Food insecurity and HIV clinical outcomes in a longitudinal study of urban homeless and marginally housed HIV-infected individuals | Aids | Weiser, S. D.; Yuan, C.; Guzman, D.; Frongillo, E. A.; Riley, E. D.; Bangsberg, D. R.; Kushel, M. B. | no | no | yes | no | Multiple | yes |
| 20534139 | Survival trends in critically ill HIV-infected patients in the highly active antiretroviral therapy era | Crit Care | Coquet, I.; Pavie, J.; Palmer, P.; Barbier, F.; Legriel, S.; Mayaux, J.; Molina, J. M.; Schlemmer, B.; Azoulay, E. | no | no | no | no | Multiple | yes |
| 25714794 | Thirty-day postoperative mortality among individuals with HIV infection receiving antiretroviral therapy and procedure-matched, uninfected comparators | JAMA Surg | King, J. T., Jr.; Perkal, M. F.; Rosenthal, R. A.; Gordon, A. J.; Crystal, S.; Rodriguez-Barradas, M. C.; Butt, A. A.; Gibert, C. L.; Rimland, D.; Simberkoff, M. S.; Justice, A. C. | no | no | no | no | Multiple | yes |
| 24000053 | Increasing quality of life and reducing HIV burden: the PATH+ intervention | AIDS Behav | Blank, M. B.; Hennessy, M.; Eisenberg, M. M. | yes | no | no | no | Multiple | yes |
| 19072715 | Missed visits and mortality among patients establishing initial outpatient HIV treatment | Clin Infect Dis | Mugavero, M. J.; Lin, H. Y.; Willig, J. H.; Westfall, A. O.; Ulett, K. B.; Routman, J. S.; Abroms, S.; Raper, J. L.; Saag, M. S.; Allison, J. J. | yes | no | yes | no | Multiple | yes |
| 24549970 | HIV-associated distal neuropathic pain is associated with smaller total cerebral cortical gray matter | J Neurovirol | Keltner, J. R.; Fennema-Notestine, C.; Vaida, F.; Wang, D.; Franklin, D. R.; Dworkin, R. H.; Sanders, C.; McCutchan, J. A.; Archibald, S. L.; Miller, D. J.; Kesidis, G.; Cushman, C.; Kim, S. M.; Abramson, I.; Taylor, M. J.; Theilmann, R. J.; Julaton, M. D.; Notestine, R. J.; Corkran, S.; Cherner, M.; Duarte, N. A.; Alexander, T.; Robinson-Papp, J.; Gelman, B. B.; Simpson, D. M.; Collier, A. C.; Marra, C. M.; Morgello, S.; Brown, G.; Grant, I.; Atkinson, J. H.; Jernigan, T. L.; Ellis, R. J. | no | no | no | yes | Neuropathic pain | yes |
| 23558760 | Safety and efficacy of transdermal buprenorphine and transdermal fentanyl in the treatment of neuropathic pain in AIDS patients | Minerva Anestesiol | Canneti, A.; Luzi, M.; Di Marco, P.; Cannata, F.; Pasqualitto, F.; Spinoglio, A.; Reale, C. | no | no | no | yes | Neuropathic pain | no |
| 17662571 | Double-blind, placebo-controlled trial of lamotrigine in combination with other medications for neuropathic pain | J Pain Symptom Manage | Silver, M.; Blum, D.; Grainger, J.; Hammer, A. E.; Quessy, S. | no | no | no | yes | Neuropathic pain | yes |
| 20124207 | Pregabalin for painful HIV neuropathy: a randomized, double-blind, placebo-controlled trial | Neurology | Simpson, D. M.; Schifitto, G.; Clifford, D. B.; Murphy, T. K.; Durso-De Cruz, E.; Glue, P.; Whalen, E.; Emir, B.; Scott, G. N.; Freeman, R. | no | no | no | yes | Neuropathic pain | no |
| 18608693 | An exploratory study of long-term neurocognitive outcomes following recovery from opportunistic brain infections in HIV+ adults | J Clin Exp Neuropsychol | Levine, A. J.; Hinkin, C. H.; Ando, K.; Santangelo, G.; Martinez, M.; Valdes-Sueiras, M.; Saxton, E. H.; Mathisen, G.; Commins, D. L.; Moe, A.; Farthing, C.; Singer, E. J. | no | no | no | no | Opportunistic brain infections | yes |
| 17243069 | Posaconazole for the treatment of azole-refractory oropharyngeal and esophageal candidiasis in subjects with HIV infection | Clin Infect Dis | Skiest, D. J.; Vazquez, J. A.; Anstead, G. M.; Graybill, J. R.; Reynes, J.; Ward, D.; Hare, R.; Boparai, N.; Isaacs, R. | no | no | no | yes | Oropharyngeal and Esophageal Candidiasis | no |
| 16575739 | A multicenter randomized trial evaluating posaconazole versus fluconazole for the treatment of oropharyngeal candidiasis in subjects with HIV/AIDS | Clin Infect Dis | Vazquez, J. A.; Skiest, D. J.; Nieto, L.; Northland, R.; Sanne, I.; Gogate, J.; Greaves, W.; Isaacs, R. | no | no | no | yes | Oropharyngeal Candidiasis | no |
| 106197133. Language: | Infection following treatment of mandible fractures: the role of immunosuppression and polysubstance abuse | Oral Surgery, Oral Medicine, Oral Pathology, Oral Radiology & Endodontology | Senel, F. C.; Jessen, G. S.; Melo, M. D.; Obeid, G. | no | no | no | yes | Osteoporosis | yes |
| 103779008. Language: | Prevalence and Predictors of Low Bone Mineral Density and Fragility Fractures Among HIV-Infected Patients at One Italian Center After Universal DXA Screening: Sensitivity and Specificity of Current Guidelines on Bone Mineral Density Management | AIDS Patient Care & STDs | Mazzotta, Elena; Ursini, Tamara; Agostinone, Adriana; Di Nicola, Angelo Domenico; Polilli, Ennio; Sozio, Federica; Vadini, Francesco; Pieri, Alessandro; Trave, Francesca; De Francesco, Valerio; Capasso, Lorenzo; Borderi, Marco; Manzoli, Lamberto; Viale, Pierluigi; Parruti, Giustino | no | no | no | yes | Osteoporosis | yes |
| 25948863 | Changes in Bone Mineral Density After Initiation of Antiretroviral Treatment With Tenofovir Disoproxil Fumarate/Emtricitabine Plus Atazanavir/Ritonavir, Darunavir/Ritonavir, or Raltegravir | J Infect Dis | Brown, T. T. and Moser, C. and Currier, J. S. and Ribaudo, H. J. | no | no | no | no | Osteoporosis | yes |
| 22207561 | CD4 count is associated with postoperative infection in patients with orthopaedic trauma who are HIV positive | Clin Orthop Relat Res | Guild, G. N.; Moore, T. J.; Barnes, W.; Hermann, C. | no | no | no | no | Othorpaedic trauma | yes |
| 21960715 | The effect of AIDS Clinical Trials Group Protocol 5164 on the time from Pneumocystis jirovecii pneumonia diagnosis to antiretroviral initiation in routine clinical practice: a case study of diffusion, dissemination, and implementation | Clin Infect Dis | Geng, E. H.; Kahn, J. S.; Chang, O. C.; Hare, C. B.; Christopoulos, K. A.; Jones, D.; Petersen, M. L.; Deeks, S. G.; Havlir, D. V.; Gandhi, M. | yes | no | no | yes | Pneumonia | yes |
| 24525448 | Community-acquired lung respiratory infections in HIV-infected patients: microbial aetiology and outcome | Eur Respir J | Cilloniz, C.; Torres, A.; Polverino, E.; Gabarrus, A.; Amaro, R.; Moreno, E.; Villegas, S.; Ortega, M.; Mensa, J.; Marcos, M. A.; Moreno, A.; Miro, J. M. | no | no | no | no | Pneumonia | yes |
| 104354298. Language: | Comparing Outcomes of HIV versus Non-HIV Patients Requiring Mechanical Ventilation | Clinical Medicine & Research | Pathak, Vikas; Samara Hurtado Rendon, Iliana; Atrash, Shebli; Prasad Rao Gagadam, Vinay; Bhunia, Kaushik; Prasad Mallampalli, Syam; Vegesna, Vijay; Mani Dangal, Mahesh; Ciubotaru, Ronald L. | no | no | no | no | Pneumonia | yes |
| 19887771 | Long-term survival after hospitalization for community-acquired and healthcare-associated pneumonia | Respiration | Cecere, L. M.; Rubenfeld, G. D.; Park, D. R.; Root, R. K.; Goss, C. H. | no | no | no | no | Pneumonia | yes |
| 16550266 | Pneumonia severity index in the immunocompromised | Can Respir J | Sanders, K. M.; Marras, T. K.; Chan, C. K. | no | no | no | no | Pneumonia | yes |
| 18796158 | Outcome of HIV-associated Pneumocystis pneumonia in hospitalized patients from 2000 through 2003 | BMC Infect Dis | Radhi, S.; Alexander, T.; Ukwu, M.; Saleh, S.; Morris, A. | no | no | no | no | Pneumonia | yes |
| 21488749 | Predictors of pneumonia severity in HIV-infected adults admitted to an Urban public hospital | AIDS Patient Care STDS | Chew, K. W.; Yen, I. H.; Li, J. Z.; Winston, L. G. | no | no | no | no | Pneumonia | yes |
| 19521925 | Severity and outcomes of Pneumocystis pneumonia in patients newly diagnosed with HIV infection: an observational cohort study | Scand J Infect Dis | Fei, M. W.; Sant, C. A.; Kim, E. J.; Swartzman, A.; Davis, J. L.; Jarlsberg, L. G.; Huang, L. | no | no | no | no | Pneumonia | yes |
| 19710335 | Predictors of mortality among women with AIDS in Illinois, USA | Int J STD AIDS | Borchardt, S. M.; Haufle, V.; Whitaker, E. E.; Dworkin, M. S. | no | no | no | no | Pneumonia, substance abuse | yes |
| 16280060 | Impact of HIV/AIDS on care and outcomes of severe sepsis | Crit Care | Mrus, J. M.; Braun, L.; Yi, M. S.; Linde-Zwirble, W. T.; Johnston, J. A. | no | no | no | no | sepsis | yes |
| 25853590 | Characteristics and Outcomes of HIV-Infected Patients With Severe Sepsis: Continued Risk in the Post-Highly Active Antiretroviral Therapy Era | Crit Care Med | Cribbs, S. K.; Tse, C.; Andrews, J.; Shenvi, N.; Martin, G. S. | no | no | no | no | Sepsis | yes |
| 20698966 | Sepsis is a major determinant of outcome in critically ill HIV/AIDS patients | Crit Care | Japiassu, A. M.; Amancio, R. T.; Mesquita, E. C.; Medeiros, D. M.; Bernal, H. B.; Nunes, E. P.; Luz, P. M.; Grinsztejn, B.; Bozza, F. A. | no | no | no | no | Sepsis | yes |
| 26039931 | Demographic and Health Services Characteristics Associated With Testing for Sexually Transmitted Infections Among a Commercially Insured Population of HIV-Positive Patients | J Acquir Immune Defic Syndr | Pearson, W. S. and Davis, A. D. | no | no | no | yes | Sexually transmitted diseases | no |
| 108202389. Language: | Self-Screening for Neisseria gonorrhoeae and Chlamydia trachomatis in the Human Immunodeficiency Virus Clinic-High Yields and High Acceptability | Sexually Transmitted Diseases | Soni, S.; White, J. A. | no | no | no | yes | Sexually transmitted diseases | yes |
| 22223813 | Gonorrhoea or chlamydia in a U.S. military HIV-positive cohort | Sex Transm Infect | Spaulding, A. B.; Lifson, A. R.; Iverson, E. R.; Ganesan, A.; Landrum, M. L.; Weintrob, A. C.; Agan, B. K.; Bavaro, M. F.; O'Connell, R. J.; Macalino, G. E. | no | no | no | yes | Sexually transmitted diseases | yes |
| 20626193 | Sexually transmitted diseases among American Indians in Arizona: an important public health disparity | Public Health Rep | Winscott, M.; Taylor, M.; Kenney, K. | no | no | no | yes | Sexually transmitted diseases | no |
| 16943224 | Serological response to syphilis treatment in HIV-positive and HIV-negative patients attending sexually transmitted diseases clinics | Sex Transm Infect | Ghanem, K. G.; Erbelding, E. J.; Wiener, Z. S.; Rompalo, A. M. | no | no | no | yes | Sexually transmitted diseases | no |
| 17447147 | Beliefs that condoms reduce sexual pleasure-gender differences in correlates among heterosexual HIV-positive injection drug users (IDUs) | J Urban Health | Mizuno, Y.; Purcell, D. W.; Latka, M. H.; Metsch, L. R.; Gomez, C. A.; Latkin, C. A. | no | no | no | yes | Sexually transmitted diseases | yes |
| 25920801 | Post-treatment drinking among HIV patients: Relationship to pre-treatment marijuana and cocaine use | Drug Alcohol Depend | Elliott, J. C. and Aharonovich, E. and Hasin, D. S. | yes | no | no | yes | Substance abuse | no |
| 16480165 | Acupuncture and spirituality-focused group therapy for the treatment of HIV-positive drug users: a preliminary study | J Psychoactive Drugs | Margolin, A.; Avants, S. K.; Arnold, R. | yes | no | no | yes | Substance abuse | no |
| 21317593 | Improved quality of life for opioid-dependent patients receiving buprenorphine treatment in HIV clinics | J Acquir Immune Defic Syndr | Korthuis, P. T.; Tozzi, M. J.; Nandi, V.; Fiellin, D. A.; Weiss, L.; Egan, J. E.; Botsko, M.; Acosta, A.; Gourevitch, M. N.; Hersh, D.; Hsu, J.; Boverman, J.; Altice, F. L. | no | no | no | yes | Substance abuse | no |
| 103949714. Language: | A Comparison of Characteristics and Outcomes of Opioid-Dependent Patients Initiating Office-Based Buprenorphine or Methadone Maintenance Treatment | Substance Abuse | Fingerhood, Michael I.; King, Van L.; Brooner, Robert K.; Rastegar, Darius A. | no | no | no | yes | Substance abuse | no |
| 25542824 | Mediators of a smoking cessation intervention for persons living with HIV/AIDS | Drug Alcohol Depend | Vidrine, D. J. | yes | no | no | yes | Substance abuse | no |
| 16838244 | Gender differences in illicit substance use among middle-aged drug users with or at risk for HIV infection | Clin Infect Dis | Hartel, D. M.; Schoenbaum, E. E.; Lo, Y.; Klein, R. S. | no | no | no | yes | Substance abuse | yes |
| 19806485 | Associations between substance use, sexual risk taking and HIV treatment adherence among homeless people living with HIV | AIDS Care | Friedman, M. S.; Marshal, M. P.; Stall, R.; Kidder, D. P.; Henny, K. D.; Courtenay-Quirk, C.; Wolitski, R. J.; Aidala, A.; Royal, S.; Holtgrave, D. R. | no | no | no | yes | Substance abuse | yes |
| 16046874 | A temporal and dose-response association between alcohol consumption and medication adherence among veterans in care | Alcohol Clin Exp Res | Braithwaite, R. S.; McGinnis, K. A.; Conigliaro, J.; Maisto, S. A.; Crystal, S.; Day, N.; Cook, R. L.; Gordon, A.; Bridges, M. W.; Seiler, J. F.; Justice, A. C. | no | no | no | yes | Substance abuse | yes |
| 20024773 | Adherence to antiretroviral therapy: a survey of factors associated with medication usage | AIDS Care | Duggan, J. M.; Locher, A.; Fink, B.; Okonta, C.; Chakraborty, J. | no | no | no | yes | Substance abuse | yes |
| 17711381 | Adherence, drug use, and treatment failure in a methadone-clinic-based program of directly administered antiretroviral therapy | AIDS Patient Care STDS | Lucas, G. M.; Mullen, B. A.; McCaul, M. E.; Weidle, P. J.; Hader, S.; Moore, R. D. | no | no | no | yes | Substance abuse | yes |
| 17942835 | Affect regulation, stimulant use, and viral load among HIV-positive persons on anti-retroviral therapy | Psychosom Med | Carrico, A. W.; Johnson, M. O.; Moskowitz, J. T.; Neilands, T. B.; Morin, S. F.; Charlebois, E. D.; Steward, W. T.; Remien, R. H.; Wong, F. L.; Rotheram-Borus, M. J.; Lightfoot, M. A.; Chesney, M. A. | no | no | no | yes | Substance abuse | yes |
| 21345624 | Alcohol consumption and depressive symptoms over time: a longitudinal study of patients with and without HIV infection | Drug Alcohol Depend | Sullivan, L. E.; Goulet, J. L.; Justice, A. C.; Fiellin, D. A. | no | no | no | yes | Substance abuse | yes |
| 17685843 | HIV-related communication and perceived norms: an analysis of the connection among injection drug users | AIDS Educ Prev | Davey-Rothwell, M. A.; Latkin, C. A. | no | no | no | yes | Substance abuse | yes |
| 17530995 | Increased mortality in rural patients with HIV in New England | AIDS Res Hum Retroviruses | Lahey, T.; Lin, M.; Marsh, B.; Curtin, J.; Wood, K.; Eccles, B.; von Reyn, C. F. | no | no | no | yes | Substance abuse | yes |
| 26009831 | Long-term Prescription of Opioids and/or Benzodiazepines and Mortality Among HIV-Infected and Uninfected Patients | J Acquir Immune Defic Syndr | Weisberg, D. F.; Gordon, K. S.; Barry, D. T.; Becker, W. C.; Crystal, S.; Edelman, E. J.; Gaither, J.; Gordon, A. J.; Goulet, J.; Kerns, R. D.; Moore, B. A.; Tate, J.; Justice, A. C.; Fiellin, D. A. | no | no | no | yes | Substance abuse | yes |
| 19662550 | Mental health in HIV seronegative and seropositive IDUs in South Florida | AIDS Care | Jones, D. L.; Waldrop-Valverde, D.; Gonzalez, P.; Mack, A.; Kumar, A. M.; Ownby, R.; Weiss, S. M.; Kumar, M. | no | no | no | yes | Substance abuse | yes |
| 20024705 | More than ancillary: HIV social services, intermediate outcomes and quality of life | AIDS Care | Chin, J. J.; Botsko, M.; Behar, E.; Finkelstein, R. | no | no | no | yes | Substance abuse | yes |
| 20024732 | Patients and family care givers' experiences around highly active antiretroviral therapy (HAART) | AIDS Care | Sacajiu, G.; Raveis, V. H.; Selwyn, P. | no | no | no | yes | Substance abuse | yes |
| 17721398 | Rethinking approaches to risk reduction for injection drug users: differences in drug type affect risk for HIV and hepatitis C virus infection through drug-injecting networks | J Acquir Immune Defic Syndr | De, P.; Cox, J.; Boivin, J. F.; Platt, R. W.; Jolly, A. M. | no | no | no | yes | Substance abuse | yes |
| 20450418 | Temporal trends in highly active antiretroviral therapy initiation among injection drug users in Baltimore, Maryland, 1996-2008 | Clin Infect Dis | Mehta, S. H.; Kirk, G. D.; Astemborski, J.; Galai, N.; Celentano, D. D. | no | no | no | yes | Substance abuse | yes |
| 18058397 | The impact of illicit drug use and substance abuse treatment on adherence to HAART | AIDS Care | Hicks, P. L.; Mulvey, K. P.; Chander, G.; Fleishman, J. A.; Josephs, J. S.; Korthuis, P. T.; Hellinger, J.; Gaist, P.; Gebo, K. A. | no | no | no | yes | Substance abuse | yes |
| 104235586. Language: | Post-Release Substance Abuse Outcomes Among HIV-Infected Jail Detainees: Results from a Multisite Study | AIDS & Behavior | Krishnan, Archana | no | no | no | yes | Substance abuse | yes |
| 21780981 | An assessment of brief group interventions to increase condom use by heterosexual crack smokers living with HIV infection | AIDS Care | Williams, M.; Bowen, A.; Atkinson, J. S.; Nilsson-Schonnesson, L.; Diamond, P. M.; Ross, M. W.; Pallonen, U. E. | no | no | no | yes | Substance abuse | yes |
| 25658949 | Cost-effectiveness analysis of brief and expanded evidence-based risk reduction interventions for HIV-infected people who inject drugs in the United States | PLoS One | Song, D. L. and Altice, F. L. and Copenhaver, M. M. and Long, E. F. | no | no | no | yes | Substance abuse | yes |
| 25314042 | Medication adherence challenges among HIV positive substance abusers: the role of food and housing insecurity | AIDS Care | Surratt, H. L.; O'Grady, C. L.; Levi-Minzi, M. A.; Kurtz, S. P. | no | no | no | yes | Substance abuse | yes |
| 25920799 | Utilizing mHealth methods to identify patterns of high risk illicit drug use | Drug Alcohol Depend | Linas, B. S. | no | no | no | yes | Substance abuse | yes |
| 18278624 | Finding meaning: African American injection drug users' interpretations of testing HIV-positive | AIDS Care | Valle, M.; Levy, J. | no | no | no | yes | Substance abuse | no |
| 22719814 | Retention on buprenorphine is associated with high levels of maximal viral suppression among HIV-infected opioid dependent released prisoners | PLoS One | Springer, S. A.; Qiu, J.; Saber-Tehrani, A. S.; Altice, F. L. | no | no | no | yes | Substance abuse | no |
| 23143526 | Risk factors for falls in HIV-infected persons | J Acquir Immune Defic Syndr | Erlandson, K. M.; Allshouse, A. A.; Jankowski, C. M.; Duong, S.; MaWhinney, S.; Kohrt, W. M.; Campbell, T. B. | no | no | no | yes | Substance abuse | no |
| 24726429 | The impact of buprenorphine/naloxone treatment on HIV risk behaviors among HIV-infected, opioid-dependent patients | Drug Alcohol Depend | Edelman, E. J.; Chantarat, T.; Caffrey, S.; Chaudhry, A.; O'Connor, P. G.; Weiss, L.; Fiellin, D. A.; Fiellin, L. E. | no | no | no | yes | Substance abuse | no |
| 16652321 | Directly administered antiretroviral therapy in methadone clinics is associated with improved HIV treatment outcomes, compared with outcomes among concurrent comparison groups | Clin Infect Dis | Lucas, G. M.; Mullen, B. A.; Weidle, P. J.; Hader, S.; McCaul, M. E.; Moore, R. D. | yes | no | no | yes | Substance abuse | no |
| 106127579. Language: | Age difference in rates of mental health/substance abuse and behavioral care in HIV-positive adults | AIDS Patient Care & STDs | Zanjani, F.; Saboe, K.; Oslin, D. | yes | no | no | yes | Substance abuse | no |
| 105064550. Corporate | Antiretroviral use among active injection-drug users: the role of patient-provider engagement and structural factors | AIDS Patient Care & STDs | Knowlton, A. R.; Arnsten, J. H.; Eldred, L. J.; Wilkinson, J. D.; Shade, S. B.; Bohnert, A. S.; Yang, C.; Wissow, L. S.; Purcell, D. W. | yes | no | no | yes | Substance abuse | no |
| 15735456 | Choosing HIV Counseling and Testing Strategies for Outreach Settings: A Randomized Trial | J Acquir Immune Defic Syndr | Spielberg, F.; Branson, B. M.; Goldbaum, G. M.; Lockhart, D.; Kurth, A.; Rossini, A.; Wood, R. W. | yes | no | no | yes | Substance abuse | no |
| 17935929 | Does readiness to change predict reduced crack use in human immunodeficiency virus prevention? | J Subst Abuse Treat | Schlosser, A. V.; Abdallah, A. B.; Callahan, C. L.; Bradford, S.; Cottler, L. B. | yes | no | no | yes | Substance abuse | no |
| 17084795 | Substance abuse treatment and receipt of liver specialty care among persons coinfected with HIV/HCV who have alcohol problems | J Subst Abuse Treat | Palepu, A.; Cheng, D. M.; Kim, T.; Nunes, D.; Vidaver, J.; Alperen, J.; Saitz, R.; Samet, J. H. | yes | no | no | yes | Substance abuse | no |
| 21302180 | Testing an optimized community-based human immunodeficiency virus (HIV) risk reduction and antiretroviral adherence intervention for HIV-infected injection drug users | Subst Abus | Copenhaver, M. M.; Lee, I. C.; Margolin, A.; Bruce, R. D.; Altice, F. L. | yes | no | no | yes | Substance abuse | no |
| 17506154 | Risk factors for methadone outside treatment programs: implications for HIV treatment among injection drug users | Addiction | Vlahov, D.; O'Driscoll, P.; Mehta, S. H.; Ompad, D. C.; Gern, R.; Galai, N.; Kirk, G. D. | no | no | no | yes | Substance abuse | yes |
| 21725250 | Neurocognitive impact of substance use in HIV infection | J Acquir Immune Defic Syndr | Byrd, D. A.; Fellows, R. P.; Morgello, S.; Franklin, D.; Heaton, R. K.; Deutsch, R.; Atkinson, J. H.; Clifford, D. B.; Collier, A. C.; Marra, C. M.; Gelman, B.; McCutchan, J. A.; Duarte, N. A.; Simpson, D. M.; McArthur, J.; Grant, I. | no | no | no | yes | Substance abuse | yes |
| 24674234 | An evaluation of hepatic enzyme elevations among HIV-infected released prisoners enrolled in two randomized placebo-controlled trials of extended release naltrexone | J Subst Abuse Treat | Vagenas, P.; Di Paola, A.; Herme, M.; Lincoln, T.; Skiest, D. J.; Altice, F. L.; Springer, S. A. | no | no | no | yes | Substance abuse | no |
| 18432391 | An experimental study of the agreement of self-administration and telephone administration of the Timeline Followback interview | J Stud Alcohol Drugs | Maisto, S. A.; Conigliaro, J. C.; Gordon, A. J.; McGinnis, K. A.; Justice, A. C. | no | no | no | yes | Substance abuse | no |
| 2014-31238-001 | Cigarette smoking and drug use among a nationally representative sample of HIVâ€positive individuals | The American Journal on Addictions | Pacek, Lauren R.; Harrell, Paul T.; Martins, Silvia S. | no | no | no | yes | Substance abuse | no |
| 23991690 | Distress tolerance and use of antiretroviral therapy among HIV-infected individuals in substance abuse treatment | AIDS Patient Care STDS | Magidson, J. F.; Seitz-Brown, C. J.; Listhaus, A.; Lindberg, B.; Anderson, K. E.; Daughters, S. B. | no | no | no | yes | Substance abuse | no |
| 18494840 | Effects of behavioral intervention on substance use among people living with HIV: the Healthy Living Project randomized controlled study | Addiction | Wong, F. L.; Rotheram-Borus, M. J.; Lightfoot, M.; Pequegnat, W.; Comulada, W. S.; Cumberland, W.; Weinhardt, L. S.; Remien, R. H.; Chesney, M.; Johnson, M. | no | no | no | yes | Substance abuse | no |
| 20099954 | Group-based randomized trial of contingencies for health and abstinence in HIV patients | J Consult Clin Psychol | Petry, N. M.; Weinstock, J.; Alessi, S. M.; Lewis, M. W.; Dieckhaus, K. | no | no | no | yes | Substance abuse | no |
| 16702714 | Health care utilization and risk behaviors among HIV positive minority drug users | J Health Care Poor Underserved | Kang, S. Y.; Goldstein, M. F.; Deren, S. | no | yes | yes | yes | Substance abuse | no |
| 24368003 | Health correlates of co-occurring substance use for women with HIV in cocaine use recovery | Addict Behav | McCabe, B. E.; Feaster, D. J.; Mitrani, V. B. | no | no | no | yes | Substance abuse | no |
| 21317590 | HIV treatment outcomes among HIV-infected, opioid-dependent patients receiving buprenorphine/naloxone treatment within HIV clinical care settings: results from a multisite study | J Acquir Immune Defic Syndr | Altice, F. L.; Bruce, R. D.; Lucas, G. M.; Lum, P. J.; Korthuis, P. T.; Flanigan, T. P.; Cunningham, C. O.; Sullivan, L. E.; Vergara-Rodriguez, P.; Fiellin, D. A.; Cajina, A.; Botsko, M.; Nandi, V.; Gourevitch, M. N.; Finkelstein, R. | no | no | no | yes | Substance abuse | no |
| 23340238 | Integrated recovery management model for ex-offenders with co-occurring mental health and substance use disorders and high rates of HIV risk behaviors | J Assoc Nurses AIDS Care | Rasch, R. F.; Davidson, D.; Seiters, J.; MacMaster, S. A.; Adams, S.; Darby, K.; Cooper, R. L. | no | no | no | yes | Substance abuse | no |
| 19142824 | Outcomes of a peer HIV prevention program with injection drug and crack users: the Risk Avoidance Partnership | Subst Use Misuse | Weeks, M. R.; Li, J.; Dickson-Gomez, J.; Convey, M.; Martinez, M.; Radda, K.; Clair, S. | no | no | no | yes | Substance abuse | no |
| 2015-38774-012 | Outcomes of a tailored intervention for cigarette smoking cessation among Latinos living with HIV/AIDS | Nicotine & Tobacco Research | Stanton, Cassandra A.; Papandonatos, George D.; Shuter, Jonathan; Bicki, Alexandra; Lloyd-Richardson, Elizabeth E.; de Dios, Marcel A.; Morrow, Kathleen M.; Makgoeng, Solomon B.; Tashima, Karen T.; Niaura, Raymond S. | yes | no | no | yes | Substance abuse | no |
| 20846009 | A cross-site, comparative effectiveness study of an integrated HIV and substance use treatment program | AIDS Patient Care STDS | Proeschold-Bell, R. J.; Heine, A.; Pence, B. W.; McAdam, K.; Quinlivan, E. B. | yes | no | yes | yes | Substance abuse | no |
| 16511419 | A randomized trial of a proactive cellular telephone intervention for smokers living with HIV/AIDS | Aids | Vidrine, D. J.; Arduino, R. C.; Lazev, A. B.; Gritz, E. R. | yes | no | no | yes | Substance abuse | no |
| 21669958 | Efficacy of cell phone-delivered smoking cessation counseling for persons living with HIV/AIDS: 3-month outcomes | Nicotine Tob Res | Vidrine, D. J.; Marks, R. M.; Arduino, R. C.; Gritz, E. R. | yes | no | no | yes | Substance abuse | no |
| 17491177 | Impact of a cell phone intervention on mediating mechanisms of smoking cessation in individuals living with HIV/AIDS | Nicotine Tob Res | Vidrine, D. J.; Arduino, R. C.; Gritz, E. R. | yes | no | no | yes | Substance abuse | no |
| 19131891 | Persistence of virological benefits following directly administered antiretroviral therapy among drug users: results from a randomized controlled trial | J Acquir Immune Defic Syndr | Maru, D. S.; Bruce, R. D.; Walton, M.; Springer, S. A.; Altice, F. L. | no | no | no | no | Substance abuse | yes |
| 20390515 | Substance abuse treatment in an urban HIV clinic: who enrolls and what are the benefits? | AIDS Care | Pisu, M.; Cloud, G.; Austin, S.; Raper, J. L.; Stewart, K. E.; Schumacher, J. E. | no | no | no | no | Substance abuse | yes |
| 21911892 | Alcohol use among patients with HIV infection | Ann Hepatol | Bonacini, M. | no | no | no | no | Substance abuse | yes |
| 18046634 | Association of alcohol abuse and injection drug use with immunologic and virologic responses to HAART in HIV-positive patients from urban community health clinics | J Community Health | Henrich, T. J.; Lauder, N.; Desai, M. M.; Sofair, A. N. | no | no | no | no | Substance abuse | yes |
| 18089980 | Microsocial environmental influences on highly active antiretroviral therapy outcomes among active injection drug users: the role of informal caregiving and household factors | J Acquir Immune Defic Syndr | Knowlton, A. R.; Arnsten, J. H.; Gourevitch, M. N.; Eldred, L.; Wilkinson, J. D.; Rose, C. D.; Buchanan, A.; Purcell, D. W. | no | no | no | no | Substance abuse | yes |
| 25151662 | Self-efficacy to quit in HIV-infected smokers | Nicotine Tob Res | Shuter, J.; Moadel, A. B.; Kim, R. S.; Weinberger, A. H.; Stanton, C. A. | no | no | no | no | Substance abuse | yes |
| 18777131 | Smoking among HIV positive New Yorkers: prevalence, frequency, and opportunities for cessation | AIDS Behav | Tesoriero, J. M.; Gieryic, S. M.; Carrascal, A.; Lavigne, H. E. | no | yes | no | no | Substance abuse | yes |
| 18823189 | The temporal relationship between alcohol consumption and HIV-medication adherence: a multilevel model of direct and moderating effects | Health Psychol | Parsons, J. T.; Rosof, E.; Mustanski, B. | no | no | no | no | Substance abuse | yes |
| 20832196 | Directly observed antiretroviral therapy improves adherence and viral load in drug users attending methadone maintenance clinics: a randomized controlled trial | Drug Alcohol Depend | Berg, K. M.; Litwin, A.; Li, X.; Heo, M.; Arnsten, J. H. | yes | no | no | no | Substance abuse | yes |
| 25123240 | Does HIV infection increase the risk of perioperative complications after THA? A nationwide database study | Clin Orthop Relat Res | Naziri, Q.; Boylan, M. R.; Issa, K.; Jones, L. C.; Khanuja, H. S.; Mont, M. A. | no | no | no | yes | Surgical complications | no |
| 22675242 | Complications of common gynecologic surgeries among HIV-infected women in the United States | Infect Dis Obstet Gynecol | Penman-Aguilar, A.; Whiteman, M. K.; Cox, S.; Posner, S. F.; Meikle, S. F.; Kourtis, A. P.; Jamieson, D. J. | no | no | no | yes | Surgical complications | no |
| 21423852 | A randomized treatment trial: single versus 7-day dose of metronidazole for the treatment of Trichomonas vaginalis among HIV-infected women | J Acquir Immune Defic Syndr | Kissinger, P.; Mena, L.; Levison, J.; Clark, R. A.; Gatski, M.; Henderson, H.; Schmidt, N.; Rosenthal, S. L.; Myers, L.; Martin, D. H. | no | no | no | yes | Trichomonas vaginalis | no |
| 20502393 | Patient-delivered partner treatment and Trichomonas vaginalis repeat infection among human immunodeficiency virus-infected women | Sex Transm Dis | Gatski, M.; Mena, L.; Levison, J.; Clark, R. A.; Henderson, H.; Schmidt, N.; Rosenthal, S. L.; Martin, D. H.; Kissinger, P. | yes | no | no | yes | Trichomonas vaginalis | no |
| 106352024. Language: | Mycobacterium africanum elicits an attenuated T cell response to early secreted antigenic target, 6 kDa, in patients with tuberculosis and their household contacts | Journal of Infectious Diseases | de Jong, B. C. and Hill, P. C. and Brookes, R. H. | no | no | no | yes | Tuberculosis | yes |
| 17030018 | B-cell immune responses in HIV positive and HIV negative patients with tuberculosis evaluated with an ELISA using a glycolipid antigen | Tuberculosis (Edinb) | Simonney, N.; Chavanet, P.; Perronne, C.; Leportier, M.; Revol, F.; Herrmann, J. L.; Lagrange, P. H. | no | no | no | yes | Tuberculosis | yes |
| 19723395 | Disparities in tuberculosis between Asian/Pacific Islanders and non-Hispanic Whites, United States, 1993-2006 | Int J Tuberc Lung Dis | Manangan, L.; Elmore, K.; Lewis, B.; Pratt, R.; Armstrong, L.; Davison, J.; Santibanez, S.; Heetderks, A.; Robison, V.; Lee, V.; Navin, T. | no | no | no | yes | Tuberculosis | yes |
| 19909501 | Factors associated with mortality in HIV-infected and uninfected patients with pulmonary tuberculosis | BMC Public Health | Mugusi, F. M.; Mehta, S.; Villamor, E.; Urassa, W.; Saathoff, E.; Bosch, R. J.; Fawzi, W. W. | no | no | no | yes | Tuberculosis | yes |
| 21813622 | Utility of endobronchial ultrasound-guided transbronchial needle aspiration in patients with tuberculous intrathoracic lymphadenopathy: a multicentre study | Thorax | Navani, N.; Molyneaux, P. L.; Breen, R. A.; Connell, D. W.; Jepson, A.; Nankivell, M.; Brown, J. M.; Morris-Jones, S.; Ng, B.; Wickremasinghe, M.; Lalvani, A.; Rintoul, R. C.; Santis, G.; Kon, O. M.; Janes, S. M. | no | no | no | yes | Tuberculosis | yes |
| 25886172 | Contribution of a heparin-binding haemagglutinin interferon-gamma release assay to the detection of Mycobacterium tuberculosis infection in HIV-infected patients: comparison with the tuberculin skin test and the QuantiFERON-TB Gold In-tube | BMC Infect Dis | Wyndham-Thomas, C. | no | no | no | yes | Tuberculosis | yes |
| 26132516 | Formative Evaluation of a Text Messaging Intervention to Promote Varenicline Adherence Among Tobacco-Dependent Persons with HIV | J Health Commun | Krebs, P.; Tseng, T. Y.; Pham, H.; Wong, S.; Sherman, S. E.; Shelley, D.; Furberg, R. D.; Wolfe, H. | yes | no | no | yes | Tuberculosis | yes |
| 104287813. Language: | Systemic Immune Activation and Microbial Translocation in Dual HIV/Tuberculosis-Infected Subjects | Journal of Infectious Diseases | Toossi, Zahra; Funderburg, Nicholas T.; Sirdeshmuk, Sohani; Whalen, Christopher C.; Nanteza, Maria W.; Johnson, Denise F.; Mayanja-Kizza, Harriet; Hirsch, Christina S. | no | no | no | yes | Tuberculosis | no |
| 25000260 | Opportunities for tuberculosis diagnosis and prevention among persons living with HIV: a cross-sectional study of policies and practices at four large Ryan White Program-Funded HIV clinics | PLoS One | Pascopella, L.; Franks, J.; Marks, S. M.; Salcedo, K.; Schmitz, K.; Colson, P. W.; Hirsch-Moverman, Y.; Flood, J.; Sayles, J. | no | no | no | yes | Tuberculosis | no |
| 25114132 | Pharmacokinetics of para-aminosalicylic acid in HIV-uninfected and HIV-coinfected tuberculosis patients receiving antiretroviral therapy, managed for multidrug-resistant and extensively drug-resistant tuberculosis | Antimicrob Agents Chemother | de Kock, L.; Sy, S. K.; Rosenkranz, B.; Diacon, A. H.; Prescott, K.; Hernandez, K. R.; Yu, M.; Derendorf, H.; Donald, P. R. | no | no | no | yes | Tuberculosis | no |
| 24902559 | Tuberculosis in the intensive care unit: a prospective observational study | Int J Tuberc Lung Dis | Balkema, C. A.; Irusen, E. M.; Taljaard, J. J.; Koegelenberg, C. F. | no | no | no | no | Tuberculosis | Yes |
| 21631429 | Response to HAART in treatment-naive HIV-infected patients with a prior diagnosis of tuberculosis or other opportunistic infections | Curr HIV Res | Dronda, F.; Sobrino, P.; Hernandez-Novoa, B.; Caro-Murillo, A. M.; Montero, M.; Iribarren, J. A.; Sanz, J.; Del Mar Alonso, M.; Labarga, P.; Bernal, E.; Moreno, S. | no | no | no | no | Tuberculosis | yes |
| 21102404 | Mortality among patients with tuberculosis and associations with HIV status --- United States, 1993-2008 | MMWR Morb Mortal Wkly Rep | . | no | no | no | no | Tuberculosis | yes |
| 107786095. Language: | RESEARCH AND PRACTICE. Mortality Hazard and Survival After Tuberculosis Treatment | American Journal of Public Health | Miller, Thaddeus L.; Wilson, Fernando A.; Pang, Jenny W.; Beavers, Suzanne; Hoger, Sally; Sharnprapai, Sharon; Pagaoa, Melissa; Katz, Dolly J.; Weis, Stephen E. | no | no | no | no | Tuberculosis | yes |
| 21396204 | Patients diagnosed with tuberculosis at death or who died during therapy: association with the human immunodeficiency virus | Int J Tuberc Lung Dis | Marks, S. M.; Magee, E.; Robison, V. | no | no | no | no | Tuberculosis | yes |
| 19803793 | HIV-specific health care utilization and mortality among tuberculosis/HIV coinfected persons | AIDS Patient Care STDS | Gadkowski, L. B.; Hamilton, C. D.; Allen, M.; Fortenberry, E. R.; Luffman, J.; Zeringue, E.; Stout, J. E. | no | no | no | no | Tuberculosis | yes |
| 25387450 | Characteristics and TB treatment outcomes in TB patients with viral hepatitis, New York City, 2000-2010 | Epidemiol Infect | Bushnell, G.; Stennis, N. L.; Drobnik, A. M.; Proops, D. C.; Ahuja, S. D.; Bornschlegel, K.; Fuld, J. | no | no | no | yes | Tuberculosis, hepatitis B, C | yes |
| 20799975 | Factors associated with mortality in patients with tuberculosis | BMC Infect Dis | Horne, D. J.; Hubbard, R.; Narita, M.; Exarchos, A.; Park, D. R.; Goss, C. H. | no | no | no | no | Tuberculosis, substance abuse | yes |
| 22424146 | Short-Term Clinical Outcomes Among Treatment-Experienced HIV-Positive Patients with Early Low Level Viremia | AIDS Patient Care & STDs | Chao, Chun; Tang, Beth; Towner, William; Silverberg, Michael J.; Hurley, Leo; Horberg, Michael | no | no | no | yes | Viremia | no |
| 16117747 | Perceived discrimination in clinical care in a nationally representative sample of HIV-infected adults receiving health care | J Gen Intern Med | Schuster, M. A.; Collins, R.; Cunningham, W. E.; Morton, S. C.; Zierler, S.; Wong, M.; Tu, W.; Kanouse, D. E. | no | yes | yes | no |  | no |
| 16053402 | HIV health care access issues for women living with HIV, mental illness, and substance abuse | AIDS Patient Care STDS | Andersen, M.; Tinsley, J.; Milfort, D.; Wilcox, R.; Smereck, G.; Pfoutz, S.; Creech, S.; Mood, D.; Smith, T.; Adams, L.; Thomas, R.; Connelly, C. | yes | yes | no | no |  | no |
| 104698940. Language: | Antidepressant Treatment and Adherence to Antiretroviral Medications Among Privately Insured Persons with HIV/AIDS | AIDS & Behavior | Akincigil, Ayse; Wilson, Ira; Walkup, James; Siegel, Michele; Huang, Cecilia; Crystal, Stephen | no | yes | no | no |  | no |
| 104471758. Language: | Approaches to Identifying Appropriate Medication Adherence Assessments for HIV Infected Individuals with Comorbid Bipolar Disorder | AIDS Patient Care & STDs | Badiee, Jayraan; Riggs, Patricia K.; Rooney, Alexandra S.; Vaida, Florin; Grant, Igor; Atkinson, J. Hampton; Moore,; the Hiv Neurobehavioral Research Program Group, David J. | no | yes | no | no |  | no |
| 103913494. Language: | Food Insecurity and Other Poverty Indicators Among People Living with HIV/AIDS: Effects on Treatment and Health Outcomes | Journal of Community Health | Kalichman, Seth; Hernandez, Dominica; Cherry, Chauncey; Kalichman, Moira; Washington, Christopher; Grebler, Tamar | no | yes | no | no |  | no |
| 104590179. Language: | Predictive Utility of Brief Alcohol Use Disorders Identification Test (AUDIT) for Human Immunodeficiency Virus Antiretroviral Medication Nonadherence | Substance Abuse | Broyles, Lauren Matukaitis; Gordon, Adam J.; Sereika, Susan M.; Ryan, Christopher M.; Erlen, Judith A. | no | yes | no | no |  | no |
| 104177133. Language: | Requesting Help to Understand Medical Information Among People Living with HIV and Poor Health Literacy | AIDS Patient Care & STDs | Kalichman, Seth; Pellowski, Jennifer; Chen, Yiyun | no | yes | no | no |  | no |
| 103795303. Language: | Synergistic effects of food insecurity and drug use on medication adherence among people living with HIV infection | Journal of Behavioral Medicine | Chen, Yiyun; Kalichman, Seth | no | yes | no | no |  | no |
| 110568092. Language: | The HIV Care Cascade Measured Over Time and by Age, Sex, and Race in a Large National Integrated Care System | AIDS Patient Care & STDs | | no | yes | no | no |  | no |
| 104171970. Language: | Development of a Multilevel Intervention to Increase HIV Clinical Trial Participation Among Rural Minorities | Health Education & Behavior | Corbie-Smith, Giselle; Odeneye, Ebun; Banks, Bahby; Shandor Miles, Margaret; Roman Isler, Malika | yes | no | no | no |  | no |
| 104049862. Language: | Identification of Evidence-Based Interventions for Promoting HIV Medication Adherence: Findings from a Systematic Review of U.S.-Based Studies, 1996-2011 | AIDS & Behavior | Charania, Mahnaz; Marshall, Khiya; Lyles, Cynthia; Crepaz, Nicole; Kay, Linda; Koenig, Linda; Weidle, Paul; Purcell, David | yes | no | no | no |  | no |
| 104527211. Language: | Implementation of targeted interventions to decrease antiretroviral-related errors in hospitalized patients | American Journal of Health-System Pharmacy | Daniels, Lindsay M.; Raasch, Ralph H.; Corbett, Amanda H. | yes | no | no | no |  | no |
| 104364186. Language: | Improved Quality of HIV Care over Time among Participants in a National Quality Improvement Initiative | Journal of Health Care for the Poor & Underserved | Chow, Wendy; Hirschhorn, Lisa R.; Ng, Darryl W.; Wells, Christopher G.; Schneider, Karen L.; Agins, Bruce D. | yes | no | no | no |  | no |
| 104568620. Language: | Longitudinal Effects of SafeTalk, a Motivational Interviewing-Based Program to Improve Safer Sex Practices Among People Living with HIV/AIDS | AIDS & Behavior | Golin, Carol; Earp, Jo; Grodensky, Catherine; Patel, Shilpa; Suchindran, Chirayath; Parikh, Megha; Kalichman, Seth; Patterson, Kristine; Swygard, Heidi; Quinlivan, E.; Amola, Kemi; Chariyeva, Zulfiya; Groves, Jennifer | yes | no | no | no |  | no |
| 103992254. Language: | A Randomized Controlled Trial of the Efficacy of a Stigma Reduction Intervention for HIV-Infected Women in the Deep South | AIDS Patient Care & STDs | Barroso, Julie; Relf, Michael V.; Williams, Megan Scull; Arscott, Joyell; Moore, Elizabeth D.; Caiola, Courtney; Silva, Susan G. | yes | no | no | no |  | no |
| 104025082. Language: | Patient Perspectives of an Integrated Program of Medical Care and Substance Use Treatment | AIDS Patient Care & STDs | Drainoni, Mari-Lynn; Farrell, Caitlin; Sorensen-Alawad, Amy; Palmisano, Joseph N.; Chaisson, Christine; Walley, Alexander Y. | yes | no | no | no |  | no |
| 20048680 | Provider-focused intervention increases adherence-related dialogue but does not improve antiretroviral therapy adherence in persons with HIV | J Acquir Immune Defic Syndr | Wilson, I. B.; Laws, M. B.; Safren, S. A.; Lee, Y.; Lu, M.; Coady, W.; Skolnik, P. R.; Rogers, W. H. | yes | no | no | no |  | no |
| 23020136 | Serostatus disclosure to sexual partners among people living with HIV: examining the roles of partner characteristics and stigma | AIDS Care | Przybyla, S. M.; Golin, C. E.; Widman, L.; Grodensky, C. A.; Earp, J. A.; Suchindran, C. | no | yes | no | no |  | no |
| 25153084 | Psychosocial predictors of non-adherence and treatment failure in a large scale multi-national trial of antiretroviral therapy for HIV: data from the ACTG A5175/PEARLS trial | PLoS One | Safren, S. A.; Biello, K. B.; Smeaton, L.; Mimiaga, M. J.; Walawander, A.; Lama, J. R.; Rana, A.; Nyirenda, M.; Kayoyo, V. M.; Samaneka, W.; Joglekar, A.; Celentano, D.; Martinez, A.; Remmert, J. E.; Nair, A.; Lalloo, U. G.; Kumarasamy, N.; Hakim, J.; Campbell, T. B. | no | yes | no | no |  | no |
| 20146111 | Testing a peer-based symptom management intervention for women living with HIV/AIDS | AIDS Care | Webel, A. R. | yes | no | no | no |  | no |
| 2011-99080-232 | Correlates of receipt of gynecologic care in HIV-infected women receiving care in the United States | NA | Odunze, Adaora N. | no | yes | no | no |  | no |
| 16045374 | A stress and coping model of medication adherence and viral load in HIV-positive men and women on highly active antiretroviral therapy (HAART) | Health Psychol | Weaver, K. E.; Llabre, M. M.; Duran, R. E.; Antoni, M. H.; Ironson, G.; Penedo, F. J.; Schneiderman, N. | no | yes | no | no |  | no |
| 16777632 | Correlates of health care utilization among HIV-seropositive injection drug users | AIDS Care | Mizuno, Y.; Wilkinson, J. D.; Santibanez, S.; Dawson Rose, C.; Knowlton, A.; Handley, K.; Gourevitch, M. N. | no | yes | yes | no |  | no |
| 16706706 | Provision of general and HIV-specific health maintenance in middle aged and older patients in an urban HIV clinic | AIDS Patient Care STDS | Sheth, A. N.; Moore, R. D.; Gebo, K. A. | no | yes | no | no |  | no |
| 16687381 | Reframing HIV adherence as part of the experience of illness | Soc Work Health Care | Golub, S. A.; Indyk, D.; Wainberg, M. L. | no | yes | no | no |  | no |
| 25867780 | Association of individual and systemic barriers to optimal medical care in people living with HIV/AIDS in Miami-Dade County | J Acquir Immune Defic Syndr | Wawrzyniak, A. J.; Rodriguez, A. E.; Falcon, A. E.; Chakrabarti, A.; Parra, A.; Park, J.; Mercogliano, K.; Villamizar, K.; Kolber, M. A.; Feaster, D. J.; Metsch, L. R. | no | yes | no | no |  | no |
| 25867774 | Barriers and facilitators to engagement of vulnerable populations in HIV primary care in New York City | J Acquir Immune Defic Syndr | Remien, R. H.; Bauman, L. J.; Mantell, J. E.; Tsoi, B.; Lopez-Rios, J.; Chhabra, R.; DiCarlo, A.; Watnick, D.; Rivera, A.; Teitelman, N.; Cutler, B.; Warne, P. | no | yes | no | no |  | no |
| 25867777 | Individual and community factors associated with geographic clusters of poor HIV care retention and poor viral suppression | J Acquir Immune Defic Syndr | Eberhart, M. G.; Yehia, B. R.; Hillier, A.; Voytek, C. D.; Fiore, D. J.; Blank, M.; Frank, I.; Metzger, D. S.; Brady, K. A. | no | yes | yes | no |  | no |
| 24941443 | Behavioral and clinical characteristics of persons receiving medical care for HIV infection - Medical Monitoring Project, United States, 2009 | MMWR Surveill Summ | Blair, J. M.; Fagan, J. L.; Frazier, E. L.; Do, A.; Bradley, H.; Valverde, E. E.; McNaghten, A.; Beer, L.; Zhang, S.; Huang, P.; Mattson, C. L.; Freedman, M. S.; Johnson, C. H.; Sanders, C. C.; Spruit-McGoff, K. E.; Heffelfinger, J. D.; Skarbinski, J. | no | yes | no | no |  | no |
| 17063136 | Health services utilization for people with HIV infection: comparison of a population targeted for outreach with the U.S. population in care | Med Care | Cunningham, W. E.; Sohler, N. L.; Tobias, C.; Drainoni, M. L.; Bradford, J.; Davis, C.; Cabral, H. J.; Cunningham, C. O.; Eldred, L.; Wong, M. D. | no | yes | yes | no |  | no |
| 23695522 | Impact of Non-HIV Related Comorbidities on Retention in HIV Medical Care: Does Retention Improve Over Time? | AIDS & Behavior | Crawford, Timothy and Sanderson, Wayne and Breheny, Patrick and Fleming, Steven and Thornton, Alice | no | yes | no | no |  | no |
| 24245846 | Positive affect promotes engagement in care after HIV diagnosis | Health Psychol | Carrico, A. W.; Moskowitz, J. T. | no | yes | no | no |  | no |
| 20024734 | Costs of care for people living with combined HIV/AIDS, chronic mental illness, and substance abuse disorders | AIDS Care | Conover, C. J.; Weaver, M.; Ang, A.; Arno, P.; Flynn, P. M.; Ettner, S. L. | no | yes | yes | no |  | no |
| 25299604 | Hispanics or Latinos living with diagnosed HIV: progress along the continuum of HIV care - United States, 2010 | MMWR Morb Mortal Wkly Rep | Gant, Z.; Bradley, H.; Hu, X.; Skarbinski, J.; Hall, H. I.; Lansky, A. | no | yes | no | no |  | no |
| 25704910 | An in-depth mixed-methods approach to Ryan White HIV/AIDS care program comprehensive needs assessment from the Northeast Georgia Public Health District: the significance of patient privacy, psychological health, and social stigma to care | Eval Program Plann | Huff, A. and Chumbler, N. and Cherry, C. O. | no | yes | no | no |  | no |
| 24797410 | Site migration in seeking care services from multiple providers is associated with worse clinical outcomes among HIV-infected individuals in Washington, DC | AIDS Care | Jia, Y.; Sengupta, D.; Opoku, J.; Wu, C.; Griffin, A.; West, T.; Samala, R.; Shaikh, I.; Pappas, G. | no | yes | no | no |  | no |
| 26179172 | Health Beliefs and Co-morbidities Associated with Appointment-Keeping Behavior Among HCV and HIV/HCV Patients | Journal of Community Health | | no | yes | no | no |  | no |
| 16938672 | Does distance affect utilization of substance abuse and mental health services in the presence of transportation services? | AIDS Care | Whetten, R.; Whetten, K.; Pence, B. W.; Reif, S.; Conover, C.; Bouis, S. | no | yes | no | no |  | no |
| 17012079 | Social relationships, stigma and adherence to antiretroviral therapy for HIV/AIDS | AIDS Care | Ware, N. C.; Wyatt, M. A.; Tugenberg, T. | no | yes | no | no |  | no |
| 25835603 | HIV Care Continuum Applied to the US Department of Veterans Affairs: HIV Virologic Outcomes in an Integrated Health Care System | J Acquir Immune Defic Syndr | Backus, L. | no | yes | no | no |  | no |
| 16255464 | Oral health findings for HIV-infected adult medical patients from the HIV Cost and Services Utilization Study | J Am Dent Assoc | Freed, J. R.; Marcus, M.; Freed, B. A.; Der-Martirosian, C.; Maida, C. A.; Younai, F. S.; Yamamoto, J. M.; Coulter, I. D.; Shapiro, M. F. | no | no | yes | no |  | no |
| 21955175 | A community-based study of barriers to HIV care initiation | AIDS Patient Care STDS | Pollini, R. A.; Blanco, E.; Crump, C.; Zuniga, M. L. | no | yes | yes | no |  | no |
| 16706709 | A comparison of HIV stigma and disclosure patterns between older and younger adults living with HIV/AIDS | AIDS Patient Care STDS | Emlet, C. A. | no | yes | no | no |  | no |
| 16706709 | A comparison of HIV stigma and disclosure patterns between older and younger adults living with HIV/AIDS | AIDS Patient Care STDS | Emlet, C. A. | no | yes | no | no |  | no |
| 16652320 | A randomized trial of directly administered antiretroviral therapy and adherence case management intervention | Clin Infect Dis | Wohl, A. R.; Garland, W. H.; Valencia, R.; Squires, K.; Witt, M. D.; Kovacs, A.; Larsen, R.; Hader, S.; Anthony, M. N.; Weidle, P. J. | no | yes | no | no |  | no |
| 18089984 | Acceptability of A-CASI by HIV-positive IDUs in a multisite, randomized, controlled trial of behavioral intervention (INSPIRE) | J Acquir Immune Defic Syndr | Mizuno, Y.; Purcell, D. W.; Mackenzie, S.; Tobin, K. E.; Wunch, T.; Arnsten, J. H.; Metsch, L. R. | no | yes | no | no |  | no |
| 22547877 | Access to oral health care and self-reported health status among low-income adults living with HIV/AIDS | Public Health Rep | Bachman, S. S.; Walter, A. W.; Umez-Eronini, A. | no | yes | yes | no |  | no |
| 19847637 | Acculturation, coping styles, and health risk behaviors among HIV positive Latinas | AIDS Behav | Sanchez, M.; Rice, E.; Stein, J.; Milburn, N. G.; Rotheram-Borus, M. J. | no | yes | no | no |  | no |
| 17978868 | Adherence to antiretroviral medication regimens: a test of a psychosocial model | AIDS Behav | Diiorio, C.; McCarty, F.; Depadilla, L.; Resnicow, K.; Holstad, M. M.; Yeager, K.; Sharma, S. M.; Morisky, D. E.; Lundberg, B. | no | yes | no | no |  | no |
| 19229685 | Adherence to antiretroviral medications and medical care in HIV-infected adults diagnosed with mental and substance abuse disorders | AIDS Care | Mellins, C. A.; Havens, J. F.; McDonnell, C.; Lichtenstein, C.; Uldall, K.; Chesney, M.; Santamaria, E. K.; Bell, J. | no | yes | no | no |  | no |
| 21346587 | Adverse health effects for individuals who move between HIV care centers | J Acquir Immune Defic Syndr | Krentz, H. B.; Worthington, H.; Gill, M. J. | no | yes | no | no |  | no |
| 17209696 | Age-associated predictors of medication adherence in HIV-positive adults: health beliefs, self-efficacy, and neurocognitive status | Health Psychol | Barclay, T. R.; Hinkin, C. H.; Castellon, S. A.; Mason, K. I.; Reinhard, M. J.; Marion, S. D.; Levine, A. J.; Durvasula, R. S. | no | yes | no | no |  | no |
| 23890194 | Are neighborhood conditions associated with HIV management? | HIV Med | Shacham, E.; Lian, M.; Onen, N. F.; Donovan, M.; Overton, E. T. | no | yes | no | no |  | no |
| 19001104 | Assessing quality of primary care provided to the HIV-infected Ryan White population in the Baltimore Eligible Metropolitan Area | Am J Med Qual | Kazi, S.; Boroumand, S. | no | no | yes | no |  | no |
| 19056866 | Association between health literacy and HIV treatment adherence: further evidence from objectively measured medication adherence | J Int Assoc Physicians AIDS Care (Chic) | Kalichman, S. C.; Pope, H.; White, D.; Cherry, C.; Amaral, C. M.; Swetzes, C.; Flanagan, J.; Kalichman, M. O. | no | yes | no | no |  | no |
| 22528619 | Association between race, depression, and antiretroviral therapy adherence in a low-income population with HIV infection | J Gen Intern Med | Kong, M. C.; Nahata, M. C.; Lacombe, V. A.; Seiber, E. E.; Balkrishnan, R. | no | yes | no | no |  | no |
| 22246513 | Association between use of specific drugs and antiretroviral adherence: findings from MACH 14 | AIDS Behav | Rosen, M. I.; Black, A. C.; Arnsten, J. H.; Goggin, K.; Remien, R. H.; Simoni, J. M.; Golin, C. E.; Bangsberg, D. R.; Liu, H. | no | yes | no | no |  | no |
| 18293137 | Associations among correlates of schedule adherence to antiretroviral therapy (ART): a path analysis of a sample of crack cocaine using sexually active African-Americans with HIV infection | AIDS Care | Atkinson, J. S.; Schonnesson, L. N.; Williams, M. L.; Timpson, S. C. | no | yes | yes | no |  | no |
| 24630628 | Barriers and facilitators to engagement in lifestyle interventions among individuals with HIV | J Assoc Nurses AIDS Care | Capili, B.; Anastasi, J. K.; Chang, M.; Ogedegbe, O. | no | yes | no | no |  | no |
| 23829330 | Barriers and facilitators to testing, treatment entry, and engagement in care by HIV-positive women of color | AIDS Patient Care STDS | Messer, L. C.; Quinlivan, E. B.; Parnell, H.; Roytburd, K.; Adimora, A. A.; Bowditch, N.; DeSousa, N. | no | yes | no | no |  | no |
| 16789855 | Barriers to antiretroviral adherence: the importance of depression, abuse, and other traumatic events | AIDS Patient Care STDS | Mugavero, M.; Ostermann, J.; Whetten, K.; Leserman, J.; Swartz, M.; Stangl, D.; Thielman, N. | no | yes | yes | no |  | no |
| 18677078 | Barriers to cervical cancer screening among low-income HIV-positive African American women | J Health Care Poor Underserved | Andrasik, M. P.; Rose, R.; Pereira, D.; Antoni, M. | no | yes | yes | no |  | no |
| 25826007 | Barriers to HIV Care and Treatment Among Participants in a Public Health HIV Care Relinkage Program | AIDS Patient Care STDS | Dombrowski, J. C.; Simoni, J. M.; Katz, D. A.; Golden, M. R. | no | yes | no | no |  | no |
| 25469916 | Baseline social characteristics and barriers to care from a special projects of national significance women of color with HIV study: a comparison of urban and rural women and barriers to HIV care | AIDS Patient Care STDS | Eastwood, E. A.; Fletcher, J.; Quinlivan, E. B.; Verdecias, N.; Birnbaum, J. M.; Blank, A. E. | no | yes | yes | no |  | no |
| 24126447 | Behind the cascade: analyzing spatial patterns along the HIV care continuum | J Acquir Immune Defic Syndr | Eberhart, M. G.; Yehia, B. R.; Hillier, A.; Voytek, C. D.; Blank, M. B.; Frank, I.; Metzger, D. S.; Brady, K. A. | no | yes | no | no |  | no |
| 25091306 | Beyond core indicators of retention in HIV care: missed clinic visits are independently associated with all-cause mortality | Clin Infect Dis | Mugavero, M. J.; Westfall, A. O.; Cole, S. R.; Geng, E. H.; Crane, H. M.; Kitahata, M. M.; Mathews, W. C.; Napravnik, S.; Eron, J. J.; Moore, R. D.; Keruly, J. C.; Mayer, K. H.; Giordano, T. P.; Raper, J. L. | no | yes | no | no |  | no |
| 18331632 | Beyond satisfaction: using the Dynamics of Care assessment to better understand patients' experiences in care | Health Qual Life Outcomes | Rapkin, B.; Weiss, E.; Chhabra, R.; Ryniker, L.; Patel, S.; Carness, J.; Adsuar, R.; Kahalas, W.; Delemarter, C.; Feldman, I.; Delorenzo, J.; Tanner, E. | no | yes | yes | no |  | no |
| 104835579. Language: | Bipolar Medication Use and Adherence to Antiretroviral Therapy Among Patients With HIV-AIDS and Bipolar Disorder | Psychiatric Services | Walkup, J. T.; Akincigil, A.; Chakravarty, S.; Olfson, M.; Bilder, S.; Amin, S.; Siegel, M. J.; Crystal, S. | no | yes | no | no |  | no |
| 22149765 | Causes of hospitalization and perceived access to care among persons newly diagnosed with HIV infection: implications for HIV testing programs | AIDS Patient Care STDS | Shahani, L.; Hartman, C.; Troisi, C.; Kapadia, A.; Giordano, T. P. | no | yes | yes | no |  | no |
| 20640593 | Changes in stress, substance use and medication beliefs are associated with changes in adherence to HIV antiretroviral therapy | AIDS Behav | French, T.; Tesoriero, J.; Agins, B. | no | yes | no | no |  | no |
| 21503833 | Clinician-assessed depression and HAART adherence in HIV-infected individuals in methadone maintenance treatment | Ann Behav Med | Gonzalez, J. S.; Psaros, C.; Batchelder, A.; Applebaum, A.; Newville, H.; Safren, S. A. | no | yes | no | no |  | no |
| 24332577 | Commitment strength, alcohol dependence and HealthCall participation: effects on drinking reduction in HIV patients | Drug Alcohol Depend | Aharonovich, E.; Stohl, M.; Ellis, J.; Amrhein, P.; Hasin, D. | no | yes | no | no |  | no |
| 2013-00413-003 | Comparing different measures of retention in outpatient HIV care | AIDS | Yehia, Baligh R.; Fleishman, John A.; Metlay, Joshua P.; Korthuis, P. Todd; Agwu, Allison L.; Berry, Stephen A.; Moore, Richard D.; Gebo, Kelly A. | no | yes | no | no |  | no |
| 23698680 | Comparing homeless and domiciled patients' utilization of the Harris County, Texas public hospital system | J Health Care Poor Underserved | Buck, D. S.; Brown, C. A.; Mortensen, K.; Riggs, J. W.; Franzini, L. | no | yes | yes | no |  | no |
| 19166089 | Comparing the costs of HIV screening strategies and technologies in health-care settings | Public Health Rep | Farnham, P. G.; Hutchinson, A. B.; Sansom, S. L.; Branson, B. M. | no | no | yes | no |  | no |
| 25665013 | Comparison of HIV outcomes for patients linked at hospital versus community-based clinics | AIDS Patient Care STDS | Schranz, A. J.; Brady, K. A.; Momplaisir, F.; Metlay, J. P.; Stephens, A.; Yehia, B. R. | no | yes | yes | no |  | no |
| 23023657 | Continuity of care in a cohort of HIV-infected former jail detainees | J Correct Health Care | Khawcharoenporn, T.; Zawitz, C.; Young, J. D.; Kessler, H. A. | no | yes | no | no |  | no |
| 21284498 | Continuity of HIV-related medical care, New York City, 2005-2009: Do patients who initiate care stay in care? | AIDS Patient Care STDS | Torian, L. V.; Wiewel, E. W. | no | yes | no | no |  | no |
| 23673792 | Contribution of substance use disorders on HIV treatment outcomes and antiretroviral medication adherence among HIV-infected persons entering jail | AIDS Behav | Chitsaz, E.; Meyer, J. P.; Krishnan, A.; Springer, S. A.; Marcus, R.; Zaller, N.; Jordan, A. O.; Lincoln, T.; Flanigan, T. P.; Porterfield, J.; Altice, F. L. | no | yes | no | no |  | no |
| 17436074 | Coping with HIV treatment side effects: conceptualization, measurement, and linkages | AIDS Behav | Johnson, M. O.; Neilands, T. B. | no | yes | no | no |  | no |
| 23161210 | Correlates of retention in HIV care after release from jail: results from a multi-site study | AIDS Behav | Althoff, A. L.; Zelenev, A.; Meyer, J. P.; Fu, J.; Brown, S. E.; Vagenas, P.; Avery, A. K.; Cruzado-Quinones, J.; Spaulding, A. C.; Altice, F. L. | no | yes | no | no |  | no |
| 26120890 | Correlation of Internet Use for Health Care Engagement Purposes and HIV Clinical Outcomes Among HIV-Positive Individuals Using Online Social Media | J Health Commun | Saberi, P.; Johnson, M. O. | no | yes | no | no |  | no |
| 21777141 | Cultural rationales guiding medication adherence among African American with HIV/AIDS | AIDS Patient Care STDS | Sankar, A.; Neufeld, S.; Berry, R.; Luborsky, M. | no | yes | yes | no |  | no |
| 22316090 | Delayed entry into HIV medical care after HIV diagnosis: risk factors and research methods | AIDS Care | Jenness, S. M.; Myers, J. E.; Neaigus, A.; Lulek, J.; Navejas, M.; Raj-Singh, S. | no | yes | no | no |  | no |
| 23180286 | Depression longitudinally mediates the association of appearance concerns to ART non-adherence in HIV-infected individuals with a history of injection drug use | J Behav Med | Blashill, A. J.; Gordon, J. R.; Safren, S. A. | no | yes | no | no |  | no |
| 16494629 | Determinants of discontinuation of initial highly active antiretroviral therapy regimens in a US HIV-infected patient cohort | HIV Med | Yuan, Y.; L'Italien, G.; Mukherjee, J.; Iloeje, U. H. | no | yes | no | no |  | no |
| 18389363 | Development and psychometric assessment of a multidimensional measure of internalized HIV stigma in a sample of HIV-positive adults | AIDS Behav | Sayles, J. N.; Hays, R. D.; Sarkisian, C. A.; Mahajan, A. P.; Spritzer, K. L.; Cunningham, W. E. | no | yes | no | no |  | no |
| 24755037 | Differences in outpatient care and treatment utilization for patients with HIV/HCV coinfection, HIV, and HCV monoinfection, a cross-sectional study | BMC Infect Dis | Johnson, T. L.; Toliver, J. C.; Mao, L.; Oramasionwu, C. U. | no | no | yes | no |  | no |
| 17943043 | Differences in the pre- and post-Katrina New Orleans HIV outpatient clinic population: who has returned? | South Med J | Clark, R. A.; Broyles, S.; Besch, L. | no | yes | no | no |  | no |
| 17584813 | Discovery of meaning and adherence to medications in HIV-infected women | J Health Psychol | Westling, E.; Garcia, K.; Mann, T. | no | yes | no | no |  | no |
| 23392459 | Disparities in engagement in care and viral suppression among persons with HIV | J Acquir Immune Defic Syndr | Muthulingam, D.; Chin, J.; Hsu, L.; Scheer, S.; Schwarcz, S. | no | yes | yes | no |  | no |
| 24463281 | Disparities in the quality of HIV care when using US Department of Health and Human Services indicators | Clin Infect Dis | Althoff, K. N.; Rebeiro, P.; Brooks, J. T.; Buchacz, K.; Gebo, K.; Martin, J.; Hogg, R.; Thorne, J. E.; Klein, M.; Gill, M. J.; Sterling, T. R.; Yehia, B.; Silverberg, M. J.; Crane, H.; Justice, A. C.; Gange, S. J.; Moore, R.; Kitahata, M. M.; Horberg, M. A. | no | yes | no | no |  | no |
| 20963630 | Do social support, stress, disclosure and stigma influence retention in HIV care for Latino and African American men who have sex with men and women? | AIDS Behav | Wohl, A. R.; Galvan, F. H.; Myers, H. F.; Garland, W.; George, S.; Witt, M.; Cadden, J.; Operskalski, E.; Jordan, W.; Carpio, F.; Lee, M. L. | no | yes | no | no |  | no |
| 16475893 | Domestic violence in barriers to health care for HIV-positive women | AIDS Patient Care STDS | Lichtenstein, B. | no | yes | yes | no |  | no |
| 22879972 | Emergency department use by released prisoners with HIV: an observational longitudinal study | PLoS One | Meyer, J. P.; Qiu, J.; Chen, N. E.; Larkin, G. L.; Altice, F. L. | no | yes | no | no |  | no |
| 19682102 | Emergency department utilization among HIV-infected patients in a multisite multistate study | HIV Med | Josephs, J. S.; Fleishman, J. A.; Korthuis, P. T.; Moore, R. D.; Gebo, K. A. | no | yes | no | no |  | no |
| 20408389 | Enrollment in outpatient care among newly released prison inmates with HIV infection | Public Health Rep | Baillargeon, J. G.; Giordano, T. P.; Harzke, A. J.; Baillargeon, G.; Rich, J. D.; Paar, D. P. | no | yes | no | no |  | no |
| 25469520 | Estimating the cost of increasing retention in care for HIV-infected patients: results of the CDC/HRSA retention in care trial | J Acquir Immune Defic Syndr | Shrestha, R. K.; Gardner, L.; Marks, G.; Craw, J.; Malitz, F.; Giordano, T. P.; Sullivan, M.; Keruly, J.; Rodriguez, A.; Wilson, T. E.; Mugavero, M. | no | yes | yes | no |  | no |
| 15916493 | Evaluation of an antiretroviral medication attitude scale and relationships between medication attitudes and medication nonadherence | AIDS Patient Care STDS | Viswanathan, H.; Anderson, R.; Thomas, J., 3rd | no | yes | yes | no |  | no |
| 104287473. Language: | Evaluation of Longitudinal Clinical Outcomes and Adherence to Care among HIV-Infected Refugees | Journal of the International Association of Providers of AIDS Care | Winston, Susanna E.; Montague, Brian T.; Lopez, Michael J.; Delong, Allison; Lemarchand, Chloe; Bedoya, Armando; Gillani, Fizza S.; Beckwith, Curt G. | no | yes | no | no |  | no |
| 23108721 | Evaluation of the single-item self-rating adherence scale for use in routine clinical care of people living with HIV | AIDS Behav | Feldman, B. J.; Fredericksen, R. J.; Crane, P. K.; Safren, S. A.; Mugavero, M. J.; Willig, J. H.; Simoni, J. M.; Wilson, I. B.; Saag, M. S.; Kitahata, M. M.; Crane, H. M. | no | yes | no | no |  | no |
| 24643444 | Examining associations between cognitive-affective vulnerability and HIV symptom severity, perceived barriers to treatment adherence, and viral load among HIV-positive adults | Int J Behav Med | Leyro, T. M.; Vujanovic, A. A.; Bonn-Miller, M. O. | no | yes | no | no |  | no |
| 23204161 | Factors associated with adherence amongst 5295 people receiving antiretroviral therapy as part of an international trial | J Infect Dis | O'Connor, J. L.; Gardner, E. M.; Mannheimer, S. B.; Lifson, A. R.; Esser, S.; Telzak, E. E.; Phillips, A. N. | no | yes | no | no |  | no |
| 19401865 | Factors associated with adherence to highly active antiretroviral therapy in homeless or unstably housed adults living with HIV | AIDS Care | Royal, S. W.; Kidder, D. P.; Patrabansh, S.; Wolitski, R. J.; Holtgrave, D. R.; Aidala, A.; Pals, S.; Stall, R. | no | yes | no | no |  | no |
| 16711309 | Factors associated with delayed initiation of HIV medical care among infected persons attending a southern HIV/AIDS clinic | South Med J | Krawczyk, C. S.; Funkhouser, E.; Kilby, J. M.; Kaslow, R. A.; Bey, A. K.; Vermund, S. H. | no | yes | yes | no |  | no |
| 22594352 | Factors associated with no or delayed linkage to care in newly diagnosed human immunodeficiency virus (HIV)-1-infected patients identified by emergency department-based rapid HIV screening programs in two urban EDs | Acad Emerg Med | Rothman, R. E.; Kelen, G. D.; Harvey, L.; Shahan, J. B.; Hairston, H.; Burah, A.; Moring-Parris, D.; Hsieh, Y. H. | no | yes | yes | no |  | no |
| 25458205 | Factors associated with retention and viral suppression among a cohort of HIV+ women of color | AIDS Patient Care STDS | Blank, A. E.; Fletcher, J.; Verdecias, N.; Garcia, I.; Blackstock, O.; Cunningham, C. | no | yes | yes | no |  | no |
| 23584606 | Factors associated with treatment initiation for psychiatric and substance use disorders among persons with HIV | Psychiatr Serv | Satre, D. D.; DeLorenze, G. N.; Quesenberry, C. P.; Tsai, A.; Weisner, C. | no | yes | no | no |  | no |
| 24022091 | Food insecurity and antiretroviral adherence among HIV positive adults who drink alcohol | J Behav Med | Kalichman, S. C.; Grebler, T.; Amaral, C. M.; McKerney, M.; White, D.; Kalichman, M. O.; Cherry, C.; Eaton, L. | no | yes | no | no |  | no |
| 23570481 | Frequent emergency department use among released prisoners with human immunodeficiency virus: characterization including a novel multimorbidity index | Acad Emerg Med | Meyer, J. P.; Qiu, J.; Chen, N. E.; Larkin, G. L.; Altice, F. L. | no | yes | yes | no |  | no |
| 22797951 | Geopolitical and cultural factors affecting ARV adherence on the US-Mexico border | J Immigr Minor Health | Shedlin, M. G.; Decena, C. U.; Beltran, O. | no | yes | yes | no |  | no |
| 104218091. Language: | Geopolitical and Cultural Factors Affecting ARV Adherence on the US-Mexico Border | Journal of Immigrant & Minority Health | Shedlin, Michele; Decena, Carlos; Beltran, Oscar | no | no | yes | no |  | no |
| 17099312 | Hazardous alcohol use: a risk factor for non-adherence and lack of suppression in HIV infection | J Acquir Immune Defic Syndr | Chander, G.; Lau, B.; Moore, R. D. | no | yes | no | no |  | no |
| 16232053 | Health care access and utilization patterns in unstably housed HIV-infected individuals in New York City | AIDS Patient Care STDS | Cunningham, C. O.; Sohler, N. L.; McCoy, K.; Heller, D.; Selwyn, P. A. | no | yes | yes | no |  | no |
| 17971562 | Health status, health care use, medication use, and medication adherence among homeless and housed people living with HIV/AIDS | Am J Public Health | Kidder, D. P.; Wolitski, R. J.; Campsmith, M. L.; Nakamura, G. V. | no | yes | yes | no |  | no |
| 23335641 | HIV diagnosis and utilisation of HIV-related medical care among foreign-born persons in New York City, 2001-2009 | Sex Transm Infect | Wiewel, E. W.; Torian, L. V.; Nasrallah, H. N.; Hanna, D. B.; Shepard, C. W. | no | yes | no | no |  | no |
| 106508703. Language: | HIV postexposure prophylaxis in sexual assault: current practice and patient adherence to treatment recommendations in a large urban teaching hospital | Academic Emergency Medicine | Linden, J. A.; Oldeg, P.; Mehta, S. D.; McCabe, K. K.; LaBelle, C. | no | no | yes | no |  | no |
| 24983302 | HIV stigma among substance abusing people living with HIV/AIDS: implications for HIV treatment | AIDS Patient Care STDS | Levi-Minzi, M. A.; Surratt, H. L. | no | yes | no | no |  | no |
| 103981922. Language: | HIV Stigma Among Substance Abusing People Living with HIV/AIDS: Implications for HIV Treatment | AIDS Patient Care & STDs | Levi-Minzi, Maria A.; Surratt, Hilary L. | no | yes | no | no |  | no |
| 23456594 | HIV stigma mechanisms and well-being among PLWH: a test of the HIV stigma framework | AIDS Behav | Earnshaw, V. A.; Smith, L. R.; Chaudoir, S. R.; Amico, K. R.; Copenhaver, M. M. | no | yes | no | no |  | no |
| 19694550 | HIV testing factors associated with delayed entry into HIV medical care among HIV-infected persons from eighteen states, United States, 2000-2004 | AIDS Patient Care STDS | Reed, J. B.; Hanson, D.; McNaghten, A. D.; Bertolli, J.; Teshale, E.; Gardner, L.; Sullivan, P. | no | yes | yes | no |  | no |
| 24714446 | HIV treatment cascade among transgender women in a San Francisco respondent driven sampling study | Sex Transm Infect | Santos, G. M.; Wilson, E. C.; Rapues, J.; Macias, O.; Packer, T.; Raymond, H. F. | no | yes | no | no |  | no |
| 20661839 | HIV-associated prospective memory impairment in the laboratory predicts failures on a semi-naturalistic measure of health care compliance | Clin Neuropsychol | Zogg, J. B.; Woods, S. P.; Weber, E.; Iudicello, J. E.; Dawson, M. S.; Grant, I. | no | yes | no | no |  | no |
| 21400307 | HIV-related medical service use by rural/urban residents: a multistate perspective | AIDS Care | Wilson, L. E.; Korthuis, T.; Fleishman, J. A.; Conviser, R.; Lawrence, P. B.; Moore, R. D.; Gebo, K. A. | no | yes | yes | no |  | no |
| 17577657 | Homelessness prevention: the effect of a shallow rent subsidy program on housing outcomes among people with HIV or AIDS | AIDS Behav | Dasinger, L. K.; Speiglman, R. | no | yes | no | no |  | no |
| 17768674 | Housing need, housing assistance, and connection to HIV medical care | AIDS Behav | Aidala, A. A.; Lee, G.; Abramson, D. M.; Messeri, P.; Siegler, A. | no | yes | no | no |  | no |
| 17133204 | Impact of enhanced services on virologic outcomes in a directly administered antiretroviral therapy trial for HIV-infected drug users | J Acquir Immune Defic Syndr | Smith-Rohrberg, D.; Mezger, J.; Walton, M.; Bruce, R. D.; Altice, F. L. | no | yes | no | no |  | no |
| 23517139 | Impact of health care payer type on HIV stage of illness at time of initiation of antiretroviral therapy in the USA | AIDS Care | Schneider, G.; Juday, T.; Wentworth, C., 3rd; Lanes, S.; Hebden, T.; Seekins, D. | no | no | yes | no |  | no |
| 24070644 | Impact of hepatitis C treatment initiation on adherence to concomitant medications | J Assoc Nurses AIDS Care | Pizzirusso, M.; Lin, J.; Head, C.; Marcus, S. M.; Ahmed, S.; Brau, N.; Weiss, J. J. | no | yes | no | no |  | no |
| 16604295 | Impact of HIV-related stigma on health behaviors and psychological adjustment among HIV-positive men and women | AIDS Behav | Vanable, P. A.; Carey, M. P.; Blair, D. C.; Littlewood, R. A. | no | yes | no | no |  | no |
| 21811144 | Increased mortality among publicly insured participants in the HIV Outpatient Study despite HAART treatment | Aids | Palella, F. J., Jr.; Baker, R. K.; Buchacz, K.; Chmiel, J. S.; Tedaldi, E. M.; Novak, R. M.; Durham, M. D.; Brooks, J. T. | no | no | yes | no |  | no |
| 20552469 | Information-motivation-behavioral skills barriers associated with intentional versus unintentional ARV non-adherence behavior among HIV+ patients in clinical care | AIDS Care | Norton, W. E.; Amico, K. R.; Fisher, W. A.; Shuper, P. A.; Ferrer, R. A.; Cornman, D. H.; Trayling, C. A.; Redding, C.; Fisher, J. D. | no | yes | no | no |  | no |
| 23551395 | Intention to adhere to HIV treatment: a patient-centred predictor of antiretroviral adherence | HIV Med | Nelsen, A.; Gupta, S.; Trautner, B. W.; Petersen, N. J.; Garza, A.; Giordano, T. P.; Naik, A. D.; Rodriguez-Barradas, M. C. | no | yes | no | no |  | no |
| 23065532 | Intentional non-adherence to medications among HIV positive alcohol drinkers: prospective study of interactive toxicity beliefs | J Gen Intern Med | Kalichman, S. C.; Grebler, T.; Amaral, C. M.; McNerey, M.; White, D.; Kalichman, M. O.; Cherry, C.; Eaton, L. | no | yes | no | no |  | no |
| 17514382 | Is patients' preferred involvement in health decisions related to outcomes for patients with HIV? | J Gen Intern Med | Beach, M. C.; Duggan, P. S.; Moore, R. D. | no | yes | no | no |  | no |
| 23054036 | Jails as an opportunity to increase engagement in HIV care: findings from an observational cross-sectional study | AIDS Behav | Avery, A. K.; Ciomcia, R. W.; Lincoln, T.; Desbrais, M.; Jordan, A. O.; Rana, A. I.; Machekano, R. | no | yes | yes | no |  | no |
| 103936198. Language: | KNOWING PARTICIPATION IN CHANGE AND PATIENT ADHERENCE TO ANTIRETROVIRAL THERAPY IN HIV INFECTED ADULTS | Visions: The Journal of Rogerian Nursing Science | Kirton, Carl A.; Morris, Diana Lynn | no | yes | no | no |  | no |
| 21877252 | Liver transplantation trends in the HIV population | Dig Dis Sci | Kemmer, N. M.; Sherman, K. E. | no | no | yes | no |  | no |
| 17594252 | Living with HIV but without medical care: barriers to engagement | AIDS Patient Care STDS | Tobias, C. R.; Cunningham, W.; Cabral, H. D.; Cunningham, C. O.; Eldred, L.; Naar-King, S.; Bradford, J.; Sohler, N. L.; Wong, M. D.; Drainoni, M. L. | no | yes | no | no |  | no |
| 23669157 | Lost or just not following up: public health effort to re-engage HIV-infected persons lost to follow-up into HIV medical care | Aids | Udeagu, C. C.; Webster, T. R.; Bocour, A.; Michel, P.; Shepard, C. W. | no | yes | no | no |  | no |
| 24274688 | Medication adherence in HIV-positive patients with diabetes or hypertension: a focus group study | BMC Health Serv Res | Monroe, A. K.; Rowe, T. L.; Moore, R. D.; Chander, G. | no | yes | no | no |  | no |
| 17503172 | Medication adherence mediates the relationship between adherence self-efficacy and biological assessments of HIV health among those with alcohol use disorders | AIDS Behav | Parsons, J. T.; Rosof, E.; Mustanski, B. | no | yes | no | no |  | no |
| 22968399 | Memory-based strategies for antiretroviral medication management: an evaluation of clinical predictors, adherence behavior awareness, and effectiveness | AIDS Behav | Blackstone, K.; Woods, S. P.; Weber, E.; Grant, I.; Moore, D. J. | no | yes | no | no |  | no |
| 22530794 | Methamphetamine use and neuropsychiatric factors are associated with antiretroviral non-adherence | AIDS Care | Moore, D. J.; Blackstone, K.; Woods, S. P.; Ellis, R. J.; Atkinson, J. H.; Heaton, R. K.; Grant, I. | no | yes | no | no |  | no |
| 17984744 | Minorities, the poor, and survivors of abuse: HIV-infected patients in the US deep South | South Med J | Pence, B. W.; Reif, S.; Whetten, K.; Leserman, J.; Stangl, D.; Swartz, M.; Thielman, N.; Mugavero, M. J. | no | no | yes | no |  | no |
| 23450876 | Missed connections: HIV-infected people never in care | Public Health Rep | Bertolli, J.; Garland, P. M.; Valverde, E. E.; Beer, L.; Fagan, J. L.; Hart, C. | no | yes | yes | no |  | no |
| 25491946 | Multilevel challenges to engagement in HIV care after prison release: a theory-informed qualitative study comparing prisoners' perspectives before and after community reentry | BMC Public Health | Haley, D. F.; Golin, C. E.; Farel, C. E.; Wohl, D. A.; Scheyett, A. M.; Garrett, J. J.; Rosen, D. L.; Parker, S. D. | no | yes | no | no |  | no |
| 20173648 | Never in care: characteristics of HIV-infected crack cocaine users in 2 US cities who have never been to outpatient HIV care | J Acquir Immune Defic Syndr | Bell, C.; Metsch, L. R.; Vogenthaler, N.; Cardenas, G.; Rodriguez, A.; Locascio, V.; Kuper, T.; Scharf, E.; Marquez, A.; Yohannan, M.; del Rio, C. | no | yes | no | no |  | no |
| 22766967 | Pain, mood, and substance abuse in HIV: implications for clinic visit utilization, antiretroviral therapy adherence, and virologic failure | J Acquir Immune Defic Syndr | Merlin, J. S.; Westfall, A. O.; Raper, J. L.; Zinski, A.; Norton, W. E.; Willig, J. H.; Gross, R.; Ritchie, C. S.; Saag, M. S.; Mugavero, M. J. | no | yes | yes | no |  | no |
| 22870846 | Pastor and lay leader perceptions of barriers and supports to HIV ministry maintenance in an African American church | J Relig Health | Stewart, J. M. | no | yes | no | no |  | no |
| 23288378 | Patient activation and improved outcomes in HIV-infected patients | J Gen Intern Med | Marshall, R.; Beach, M. C.; Saha, S.; Mori, T.; Loveless, M. O.; Hibbard, J. H.; Cohn, J. A.; Sharp, V. L.; Korthuis, P. T. | no | yes | no | no |  | no |
| 17284499 | Patient-related factors predicting HIV medication adherence among men and women with alcohol problems | J Health Psychol | Parsons, J. T.; Rosof, E.; Mustanski, B. | no | yes | no | no |  | no |
| 23250298 | Patterns and correlates of linkage to appropriate HIV care after HIV diagnosis in the US Medicaid population | Sex Transm Dis | Johnston, S. S.; Juday, T.; Seekins, D.; Hebden, T.; Fulcher, N.; Farr, A. M.; Chu, B. C.; Mullins, C. D. | no | yes | no | no |  | no |
| 16548714 | Patterns of HIV care for patients with serious mental illness | AIDS Patient Care STDS | Bogart, L. M.; Fremont, A. M.; Young, A. S.; Pantoja, P.; Chinman, M.; Morton, S.; Koegel, P.; Sullivan, G.; Kanouse, D. E. | no | yes | no | no |  | no |
| 23334359 | Personal health record use and its association with antiretroviral adherence: survey and medical record data from 1871 US veterans infected with HIV | AIDS Behav | Keith McInnes, D.; Shimada, S. L.; Rao, S. R.; Quill, A.; Duggal, M.; Gifford, A. L.; Brandt, C. A.; Houston, T. K.; Ohl, M. E.; Gordon, K. S.; Mattocks, K. M.; Kazis, L. E.; Justice, A. C. | no | yes | no | no |  | no |
| 23143751 | Personal HIV knowledge, appointment adherence and HIV outcomes | AIDS Behav | Jones, D.; Cook, R.; Rodriguez, A.; Waldrop-Valverde, D. | no | yes | yes | no |  | no |
| 24801492 | Personal meaning, social support, and perceived stigma in individuals receiving HIV mental health services | J Clin Psychol Med Settings | Farber, E. W.; Lamis, D. A.; Shahane, A. A.; Campos, P. E. | no | yes | no | no |  | no |
| 20711651 | Persons newly diagnosed with HIV infection are at high risk for depression and poor linkage to care: results from the Steps Study | AIDS Behav | Bhatia, R.; Hartman, C.; Kallen, M. A.; Graham, J.; Giordano, T. P. | no | yes | yes | no |  | no |
| 23605401 | Polypharmacy and risk of antiretroviral drug interactions among the aging HIV-infected population | J Gen Intern Med | Holtzman, C.; Armon, C.; Tedaldi, E.; Chmiel, J. S.; Buchacz, K.; Wood, K.; Brooks, J. T. | no | no | yes | no |  | no |
| 24377076 | Poorer neuropsychological performance increases risk for social services among HIV-infected individuals | Hawaii J Med Public Health | Umaki, T. M.; Gangcuangco, L. M.; Chow, D. C.; Nakamoto, B. K.; Marotz, L.; Kallianpur, K. J.; Shikuma, C. M. | no | yes | yes | no |  | no |
| 15664714 | Predictors of substance use frequency and reductions in seriousness of use among persons living with HIV | Drug Alcohol Depend | Lightfoot, M.; Rogers, T.; Goldstein, R.; Rotheram-Borus, M. J.; May, S.; Kirshenbaum, S.; Weinhardt, L.; Zadoretzky, C.; Kittel, L.; Johnson, M.; Gore-Felton, C.; Morin, S. F. | no | yes | yes | no |  | no |
| 16813512 | Prevalence of complementary and alternative medicine use among HIV patients for perceived lipodystrophy | J Altern Complement Med | Cho, M.; Ye, X.; Dobs, A.; Cofrancesco, J., Jr. | no | yes | yes | no |  | no |
| 24592813 | Preventive health care among HIV positive women in a Utah HIV/AIDS clinic: a retrospective cohort study | BMC Womens Health | Simonsen, S. E.; Kepka, D.; Thompson, J.; Warner, E. L.; Snyder, M.; Ries, K. M. | no | yes | no | no |  | no |
| 24464408 | Provider-patient communication about adherence to anti-retroviral regimens differs by patient race and ethnicity | AIDS Behav | Laws, M. B.; Lee, Y.; Rogers, W. H.; Beach, M. C.; Saha, S.; Korthuis, P. T.; Sharp, V.; Cohn, J.; Moore, R.; Wilson, I. B. | no | yes | no | no |  | no |
| 21145198 | Psychometric properties of a Symptom Management Self-Efficacy Scale for women living with HIV/AIDS | J Pain Symptom Manage | Webel, A. R.; Okonsky, J. | no | yes | no | no |  | no |
| 19921420 | Psychosocial factors associated with successful transition into HIV case management for those without primary care in an urban area | AIDS Behav | Johnson, D.; Polansky, M.; Matosky, M.; Teti, M. | no | yes | no | no |  | no |
| 18701730 | Racial, gender and geographic disparities of antiretroviral treatment among US Medicaid enrolees in 1998 | J Epidemiol Community Health | King, W. D.; Minor, P.; Ramirez Kitchen, C.; Ore, L. E.; Shoptaw, S.; Victorianne, G. D.; Rust, G. | no | yes | no | no |  | no |
| 18580614 | Rates of hospitalizations and associated diagnoses in a large multisite cohort of HIV patients in the United States, 1994-2005 | Aids | Buchacz, K.; Baker, R. K.; Moorman, A. C.; Richardson, J. T.; Wood, K. C.; Holmberg, S. D.; Brooks, J. T. | no | no | yes | no |  | no |
| 104438903. Language: | Reaching Haitian Americans Living With HIV/AIDS: Met and Unmet Health Care Needs | Journal of HIV/AIDS & Social Services | DeRigne, LeaAnne; Choi, Jung Jin; Barsky, Allan E.; Albertini, Velmarie | no | yes | no | no |  | no |
| 24056066 | Receipt of HIV/STD prevention counseling by HIV-infected adults receiving medical care in the United States | Aids | Mizuno, Y.; Zhu, J.; Crepaz, N.; Beer, L.; Purcell, D. W.; Johnson, C. H.; Valverde, E. E.; Skarbinski, J. | no | yes | no | no |  | no |
| 17675724 | Recently released with HIV/AIDS: primary care treatment needs and experiences | J Health Care Poor Underserved | Fontana, L.; Beckerman, A. | no | yes | yes | no |  | no |
| 16475891 | Religious beliefs, practices and treatment adherence among individuals with HIV in the southern United States | AIDS Patient Care STDS | Parsons, S. K.; Cruise, P. L.; Davenport, W. M.; Jones, V. | no | yes | no | no |  | no |
| 23242158 | Retention among North American HIV-infected persons in clinical care, 2000-2008 | J Acquir Immune Defic Syndr | Rebeiro, P.; Althoff, K. N.; Buchacz, K.; Gill, J.; Horberg, M.; Krentz, H.; Moore, R.; Sterling, T. R.; Brooks, J. T.; Gebo, K. A.; Hogg, R.; Klein, M.; Martin, J.; Mugavero, M.; Rourke, S.; Silverberg, M. J.; Thorne, J.; Gange, S. J. | no | yes | no | no |  | no |
| 2013-05118-009 | Retention among North American HIV-infected persons in clinical care, 2000â€“2008 | JAIDS Journal of Acquired Immune Deficiency Syndromes | Rebeiro, Peter; Althoff, Keri N.; Buchacz, Kate; Gill, John; Horberg, Michael; Krentz, Hartmut; Moore, Richard; Sterling, Timothy R.; Brooks, John T.; Gebo, Kelly A.; Hogg, Robert; Klein, Marina; Martin, Jeffrey; Mugavero, Michael; Rourke, Sean; Silverberg, Michael J.; Thorne, Jennifer; Gange, Stephen J. | no | yes | no | no |  | no |
| 24493009 | Retention in care within 1 year of initial HIV care visit in a multisite US cohort: who's in and who's out? | J Int Assoc Provid AIDS Care | Tedaldi, E. M.; Richardson, J. T.; Debes, R.; Young, B.; Chmiel, J. S.; Durham, M. D.; Brooks, J. T.; Buchacz, K. | no | yes | yes | no |  | no |
| 22547876 | Retention of people living with HIV/AIDS in oral health care | Public Health Rep | Tobias, C. R.; Fox, J. E.; Walter, A. W.; Lemay, C. A.; Abel, S. N. | no | yes | yes | no |  | no |
| 19925308 | Routine screening for depression: identifying a challenge for successful HIV care | AIDS Patient Care STDS | Shacham, E.; Nurutdinova, D.; Satyanarayana, V.; Stamm, K.; Overton, E. T. | no | yes | no | no |  | no |
| 20210646 | Routine, self-administered, touch-screen, computer-based suicidal ideation assessment linked to automated response team notification in an HIV primary care setting | Clin Infect Dis | Lawrence, S. T.; Willig, J. H.; Crane, H. M.; Ye, J.; Aban, I.; Lober, W.; Nevin, C. R.; Batey, D. S.; Mugavero, M. J.; McCullumsmith, C.; Wright, C.; Kitahata, M.; Raper, J. L.; Saag, M. S.; Schumacher, J. E. | no | yes | yes | no |  | no |
| 19229695 | Self-efficacy and depression as mediators of the relationship between pain and antiretroviral adherence | AIDS Care | Berg, K. M.; Cooperman, N. A.; Newville, H.; Arnsten, J. H. | no | yes | no | no |  | no |
| 104438902. Language: | Social and Clinical Determinants Influencing HIV Treatment Among Hoosiers | Journal of HIV/AIDS & Social Services | Hillman, Daniel; Carelock, Brittney; Tobias, Jodi; Connor, Michael; Roseberry, Jeremy; Lofton, Stephanie; Carnicom, Lois | no | yes | no | no |  | no |
| 22871482 | Social support networks and primary care use by HIV-infected drug users | J Assoc Nurses AIDS Care | Ramaswamy, M.; Kelly, P. J.; Li, X.; Berg, K. M.; Litwin, A. H.; Arnsten, J. H. | no | yes | yes | no |  | no |
| 23311323 | Social-cognitive correlates of antiretroviral therapy adherence among HIV-infected individuals receiving infectious disease care in a medium-sized northeastern US city | AIDS Care | Brown, J. L.; Littlewood, R. A.; Vanable, P. A. | no | yes | no | no |  | no |
| 19110909 | Sociodemographic factors predict early discontinuation of HIV non-nucleoside reverse transcriptase inhibitors and protease inhibitors | J Natl Med Assoc | Asad, S.; Hulgan, T.; Raffanti, S. P.; Daugherty, J.; Ray, W.; Sterling, T. R. | no | yes | yes | no |  | no |
| 20716711 | Stress and poverty predictors of treatment adherence among people with low-literacy living with HIV/AIDS | Psychosom Med | Kalichman, S. C.; Grebler, T. | no | yes | no | no |  | no |
| 20703792 | Substance use and the quality of patient-provider communication in HIV clinics | AIDS Behav | Korthuis, P. T.; Saha, S.; Chander, G.; McCarty, D.; Moore, R. D.; Cohn, J. A.; Sharp, V. L.; Beach, M. C. | no | yes | no | no |  | no |
| 22350832 | Technology use and reasons to participate in social networking health websites among people living with HIV in the US | AIDS Behav | Horvath, K. J.; Danilenko, G. P.; Williams, M. L.; Simoni, J.; Amico, K. R.; Oakes, J. M.; Simon Rosser, B. R. | no | yes | yes | no |  | no |
| 23075914 | The association between diet and physical activity on insulin resistance in the Women's Interagency HIV Study | J Acquir Immune Defic Syndr | Hessol, N. A.; Ameli, N.; Cohen, M. H.; Urwin, S.; Weber, K. M.; Tien, P. C. | no | no | yes | no |  | no |
| 19653047 | The association of stigma with self-reported access to medical care and antiretroviral therapy adherence in persons living with HIV/AIDS | J Gen Intern Med | Sayles, J. N.; Wong, M. D.; Kinsler, J. J.; Martins, D.; Cunningham, W. E. | no | no | yes | no |  | no |
| 106180571. Language: | The effect of perceived stigma from a health care provider on access to care among a low-income HIV-positive population | AIDS Patient Care & STDs | Kinsler, J. J.; Wong, M. D.; Sayles, J. N.; Davis, C.; Cunningham, W. E. | no | yes | no | no |  | no |
| 19742170 | The effects of environmental factors on persons living with HIV/AIDS | Int J Environ Res Public Health | Nichols, L.; Tchounwou, P. B.; Mena, L.; Sarpong, D. | no | yes | no | no |  | no |
| 18770023 | The effects of HIV stigma on health, disclosure of HIV status, and risk behavior of homeless and unstably housed persons living with HIV | AIDS Behav | Wolitski, R. J.; Pals, S. L.; Kidder, D. P.; Courtenay-Quirk, C.; Holtgrave, D. R. | no | yes | no | no |  | no |
| 20397898 | The interplay of sociodemographic factors on virologic suppression among a U.S. outpatient HIV clinic population | AIDS Patient Care STDS | Shacham, E.; Nurutdinova, D.; Onen, N.; Stamm, K.; Overton, E. T. | no | yes | no | no |  | no |
| 17563293 | The provider role in client engagement in HIV care | AIDS Patient Care STDS | Mallinson, R. K.; Rajabiun, S.; Coleman, S. | no | yes | no | no |  | no |
| 15607387 | The relationship between HAART use and employment for HIV-positive individuals: an empirical analysis and policy outlook | Health Policy | Bernell, S. L.; Shinogle, J. A. | no | yes | yes | no |  | no |
| 19055408 | The therapeutic implications of timely linkage and early retention in HIV care | AIDS Patient Care STDS | Ulett, K. B.; Willig, J. H.; Lin, H. Y.; Routman, J. S.; Abroms, S.; Allison, J.; Chatham, A.; Raper, J. L.; Saag, M. S.; Mugavero, M. J. | no | yes | yes | no |  | no |
| 25329710 | Timing of antiretroviral therapy initiation in a nationally representative sample of HIV-infected adults receiving medical care in the United States | AIDS Patient Care STDS | Adedinsewo, D. A.; Wei, S. C.; Robertson, M.; Rose, C.; Johnson, C. H.; Dombrowski, J.; Skarbinski, J. | no | no | yes | no |  | no |
| 23876086 | Transportation vulnerability as a barrier to service utilization for HIV-positive individuals | AIDS Care | Sagrestano, L. M.; Clay, J.; Finerman, R.; Gooch, J.; Rapino, M. | no | yes | no | no |  | no |
| 24141487 | Travel distance to HIV medical care: a geographic analysis of weighted survey data from the Medical Monitoring Project in Philadelphia, PA | AIDS Behav | Eberhart, M. G.; Voytek, C. D.; Hillier, A.; Metzger, D. S.; Blank, M. B.; Brady, K. A. | no | yes | no | no |  | no |
| 23555868 | Treatment outcomes in undocumented Hispanic immigrants with HIV infection | PLoS One | Poon, K. K.; Dang, B. N.; Davila, J. A.; Hartman, C.; Giordano, T. P. | no | yes | yes | no |  | no |
| 22643464 | Trust in primary care providers and antiretroviral adherence in an urban HIV clinic | J Health Care Poor Underserved | Blackstock, O. J.; Addison, D. N.; Brennan, J. S.; Alao, O. A. | no | yes | no | no |  | no |
| 23797695 | Understanding the disparity: predictors of virologic failure in women using highly active antiretroviral therapy vary by race and/or ethnicity | J Acquir Immune Defic Syndr | McFall, A. M.; Dowdy, D. W.; Zelaya, C. E.; Murphy, K.; Wilson, T. E.; Young, M. A.; Gandhi, M.; Cohen, M. H.; Golub, E. T.; Althoff, K. N. | no | no | yes | no |  | no |
| 22731500 | Using laboratory surveillance data to estimate engagement in care among persons living with HIV in Los Angeles County, 2009 | AIDS Patient Care STDS | Hu, Y. W.; Kinsler, J. J.; Sheng, Z.; Kang, T.; Bingham, T.; Frye, D. M. | no | yes | no | no |  | no |
| 17428185 | Utilization of health care services in hard-to-reach marginalized HIV-infected individuals | AIDS Patient Care STDS | Cunningham, C. O.; Sohler, N. L.; Wong, M. D.; Relf, M.; Cunningham, W. E.; Drainoni, M. L.; Bradford, J.; Pounds, M. B.; Cabral, H. D. | no | yes | yes | no |  | no |
| 18197121 | Utilization of mental health and substance abuse care for people living with HIV/AIDS, chronic mental illness, and substance abuse disorders | J Acquir Immune Defic Syndr | Weaver, M. R.; Conover, C. J.; Proescholdbell, R. J.; Arno, P. S.; Ang, A.; Ettner, S. L. | no | yes | yes | no |  | no |
| 16088365 | Variations in patterns of highly active antiretroviral therapy (HAART) adherence | AIDS Behav | Levine, A. J.; Hinkin, C. H.; Castellon, S. A.; Mason, K. I.; Lam, M. N.; Perkins, A.; Robinet, M.; Longshore, D.; Newton, T.; Myers, H.; Durvasula, R. S.; Hardy, D. J. | no | yes | no | no |  | no |
| 20166788 | Violence, coping, and consistent medication adherence in HIV-positive couples | AIDS Educ Prev | Lopez, E. J.; Jones, D. L.; Villar-Loubet, O. M.; Arheart, K. L.; Weiss, S. M. | no | yes | no | no |  | no |
| 24085706 | Viral suppression and antiretroviral medication adherence among alcohol using HIV-positive adults | Int J Behav Med | Kalichman, S. C.; Grebler, T.; Amaral, C. M.; McNerney, M.; White, D.; Kalichman, M. O.; Cherry, C.; Eaton, L. | no | yes | no | no |  | no |
| 16938670 | Characteristics of HIV-infected adults in the Deep South and their utilization of mental health services: A rural vs. urban comparison | AIDS Care | Reif, S.; Whetten, K.; Ostermann, J.; Raper, J. L. | no | yes | yes | no |  | no |
| 109837664. Language: | Evaluation of a Community Health Worker Intervention to Reduce HIV/AIDS Stigma and Increase HIV Testing Among Underserved Latinos in the Southwestern U.S | Public Health Reports | Rios-Ellis, Britt; Becker, Davida; Espinoza, Lilia; Nguyen-Rodriguez, Selena; Diaz, Gaby; Carricchi, Ana; Galvez, Gino; Garcia, Melawhy | no | yes | no | no |  | no |
| 16938674 | Factors associated with fewer visits for HIV primary care at a tertiary care center in the Southeastern U.S | AIDS Care | Napravnik, S.; Eron, J. J., Jr.; McKaig, R. G.; Heine, A. D.; Menezes, P.; Quinlivan, E. | no | yes | yes | no |  | no |
| 24500286 | Progress along the continuum of HIV care among blacks with diagnosed HIV- United States, 2010 | MMWR Morb Mortal Wkly Rep | Whiteside, Y. O.; Cohen, S. M.; Bradley, H.; Skarbinski, J.; Hall, H. I.; Lansky, A. | no | yes | no | no |  | no |
| 25033144 | Retention in care and viral suppression among persons living with HIV/AIDS in New York City, 2006-2010 | Am J Public Health | Torian, L. V.; Xia, Q.; Wiewel, E. W. | no | yes | no | no |  | no |
| 25615029 | Barriers to antiretroviral therapy adherence and plasma HIV RNA suppression among AIDS clinical trials group study participants | AIDS Patient Care STDS | Saberi, P.; Neilands, T. B.; Vittinghoff, E.; Johnson, M. O.; Chesney, M.; Cohn, S. E. | no | yes | no | no |  | no |
| 25634492 | Multiple gaps in care common among newly diagnosed HIV patients | AIDS Care | Rana, A. I. | no | yes | no | no |  | no |
| 25240628 | Patterns of HIV service use and HIV viral suppression among patients treated in an academic infectious diseases clinic in North Carolina | AIDS Behav | Palma, A. and Lounsbury, D. W. and Messer, L. and Quinlivan, E. B. | no | yes | yes | no |  | no |
| 21711165 | Problems taking pills: understanding HIV medication adherence from a new perspective | AIDS Care | Okonsky, J. G. | no | yes | no | no |  | no |
| 25572828 | The relationship between ART adherence and smoking status among HIV+ individuals | AIDS Behav | Moreno, J. L. | no | yes | no | no |  | no |
| 18483850 | Implementation of the Medicare Part D prescription drug benefit is associated with antiretroviral therapy interruptions | AIDS Behav | Das-Douglas, M.; Riley, E. D.; Ragland, K.; Guzman, D.; Clark, R.; Kushel, M. B.; Bangsberg, D. R. | no | yes | yes | no |  | no |
| 24745475 | Patterns of substance use among HIV-positive adults over 50: implications for treatment and medication adherence | Drug Alcohol Depend | Parsons, J. T.; Starks, T. J.; Millar, B. M.; Boonrai, K.; Marcotte, D. | no | yes | no | no |  | no |
| 23802143 | Diagnosing HIV infection in primary care settings: missed opportunities | AIDS Patient Care STDS | Chin, T.; Hicks, C.; Samsa, G.; McKellar, M. | no | no | yes | no |  | no |
| 18190326 | Low rates of antiretroviral therapy among HIV-infected patients with chronic kidney disease | Clin Infect Dis | Choi, A. I.; Rodriguez, R. A.; Bacchetti, P.; Volberding, P. A.; Havlir, D.; Bertenthal, D.; Bostrom, A.; O'Hare, A. M. | no | yes | no | no |  | no |
| 19444661 | Adherence to antiretroviral medication in older adults living with HIV/AIDS: a comparison of alternative models | AIDS Care | Johnson, C. J.; Heckman, T. G.; Hansen, N. B.; Kochman, A.; Sikkema, K. J. | no | yes | no | no |  | no |
| 24256631 | Impact of hepatitis coinfection on hospitalization rates and causes in a multicenter cohort of persons living with HIV | J Acquir Immune Defic Syndr | Crowell, T. A.; Gebo, K. A.; Balagopal, A.; Fleishman, J. A.; Agwu, A. L.; Berry, S. A. | no | no | yes | no |  | no |
| 24770984 | The unique challenges facing HIV-positive patients who smoke cigarettes: HIV viremia, ART adherence, engagement in HIV care, and concurrent substance use | AIDS Behav | O'Cleirigh, C.; Valentine, S. E.; Pinkston, M.; Herman, D.; Bedoya, C. A.; Gordon, J. R.; Safren, S. A. | no | yes | no | no |  | no |
| 24432878 | Gender disparities in HIV treatment outcomes following release from jail: results from a multicenter study | Am J Public Health | Meyer, J. P.; Zelenev, A.; Wickersham, J. A.; Williams, C. T.; Teixeira, P. A.; Altice, F. L. | no | yes | no | no |  | no |
| 18441253 | General health status and adherence to antiretroviral therapy | J Int Assoc Physicians AIDS Care (Chic) | Cardarelli, R.; Weis, S.; Adams, E.; Radaford, D.; Vecino, I.; Munguia, G.; Johnson, K. L.; Fulda, K. G. | no | yes | no | no |  | no |
| 20542858 | Sharing health data for better outcomes on PatientsLikeMe | J Med Internet Res | Wicks, P.; Massagli, M.; Frost, J.; Brownstein, C.; Okun, S.; Vaughan, T.; Bradley, R.; Heywood, J. | yes | no | no | no |  | no |
| 17131980 | The efficacy of distant healing for human immunodeficiency virus--results of a randomized trial | Altern Ther Health Med | Astin, J. A.; Stone, J.; Abrams, D. I.; Moore, D. H.; Couey, P.; Buscemi, R.; Targ, E. | yes | no | no | no |  | no |
| 20216478 | Provider and client acceptance of a health department enhanced approach to improve HIV partner notification in New York City | Sex Transm Dis | Udeagu, C. C.; Bocour, A.; Gale, I.; Begier, E. M. | yes | no | no | no |  | no |
| 18240894 | HIV peer counseling and the development of hope: perspectives from peer counselors and peer counseling recipients | AIDS Patient Care STDS | Harris, G. E.; Larsen, D. | yes | no | no | no |  | no |
| 22327809 | The Community Liaison Program: a health education pilot program to increase minority awareness of HIV and acceptance of HIV vaccine trials | Health Educ Res | Kelley, R. T.; Hannans, A.; Kreps, G. L.; Johnson, K. | yes | no | no | no |  | no |
| 15843117 | Group-level interventions for persons living with HIV: a catalyst for individual change | AIDS Educ Prev | Hyde, J.; Appleby, P. R.; Weiss, G.; Bailey, J.; Morgan, X. | yes | no | no | no |  | no |
| 15782514 | Health and economic impacts of an HIV intervention in out of treatment substance abusers: evidence from a dynamic model | Health Care Manag Sci | Richter, A.; Loomis, B. | yes | no | no | no |  | no |
| 16777637 | Locating and linking to medical care HIV-positive persons without a history of care: findings from the California Bridge Project | AIDS Care | Molitor, F.; Waltermeyer, J.; Mendoza, M.; Kuenneth, C.; Aguirre, A.; Brockmann, K.; Crump, C. | yes | yes | no | no |  | no |
| 16623624 | Positive provider interactions, adherence self-efficacy, and adherence to antiretroviral medications among HIV-infected adults: A mediation model | AIDS Patient Care STDS | Johnson, M. O.; Chesney, M. A.; Goldstein, R. B.; Remien, R. H.; Catz, S.; Gore-Felton, C.; Charlebois, E.; Morin, S. F. | yes | yes | no | no |  | no |
| 20178025 | A multicultural approach to HIV prevention within a residential chemical dependency treatment program: the Positive Steps Program | J Evid Based Soc Work | Harris, E.; Kiekel, P.; Brown, K.; Sarmiento, A.; Byock, G. | yes | yes | no | no |  | no |
| 20178027 | Effects of drug use on sexual risk behavior: results of an HIV outreach and education program | J Evid Based Soc Work | Lauby, J. L.; Batson, H.; Milnamow, M. | yes | no | no | no |  | no |
| 19346565 | Cost-effectiveness analysis of integrated care for people with HIV, chronic mental illness and substance abuse disorders | J Ment Health Policy Econ | Weaver, M. R.; Conover, C. J.; Proescholdbell, R. J.; Arno, P. S.; Ang, A.; Uldall, K. K.; Ettner, S. L. | yes | no | yes | no |  | no |
| 103765859. Language: | Case Management: Steadfast Resource for Addressing Linkage to Care and Prevention With Hospitalized HIV-Infected Crack Users | Journal of HIV/AIDS & Social Services | Kenya, Sonjia; Chida, Natasha; Cardenas, Gabriel; Pereyra, Margaret; Del Rio, Carlos; Rodriguez, Allan; Metsch, Lisa | yes | no | no | no |  | no |
| 25867782 | Benefits of a routine opt-out HIV testing and linkage to care program for previously diagnosed patients in publicly funded emergency departments in Houston, TX | J Acquir Immune Defic Syndr | Flash, C. A.; Pasalar, S.; Hemmige, V.; Davila, J. A.; Hallmark, C. J.; McNeese, M.; Miertschin, N.; Ruggerio, M. C.; Giordano, T. P. | yes | no | no | no |  | no |
| 106274417. Language: | Continuing to provide and fund a mix of Ryan White Title I HIV/AIDS services: support from 'The Voices of Experience' needs surveys in Massachusetts | Journal of HIV/AIDS & Social Services | Beinecke, R. H.; Matava, M. A.; Rivers, N.; Stevens, R.; Goldrosen, G.; Averbach, A. R.; Woliver, R.; Kelley, B. | yes | yes | yes | no |  | no |
| 18220096 | The New Orleans HIV outpatient program patient experience with Hurricane Katrina | J La State Med Soc | Clark, R. A.; Mirabelli, R.; Shafe, J.; Broyles, S.; Besch, L.; Kissinger, P. | yes | no | no | no |  | no |
| 18957732 | Balancing health, work, and daily life: design and evaluation of a pilot intervention for persons with HIV/AIDS | Work | Bedell, G. | yes | yes | no | no |  | no |
| 22527264 | Exploring ART Intake Scenes in a Human Rights-Based Intervention to Improve Adherence: A Randomized Controlled Trial | AIDS & Behavior | Basso, CÃ¡ritas; Helena, Ernani; Caraciolo, Joselita; Paiva, Vera; Nemes, Maria | yes | no | no | no |  | no |
| 26485232 | Healing Our Women for Transgender Women: Adaptation, Acceptability, and Pilot Testing | AIDS Education & Prevention | Collier | yes | no | no | no |  | no |
| 18230008 | Meta-analysis of cognitive-behavioral interventions on HIV-positive persons' mental health and immune functioning | Health Psychol | Crepaz, N.; Passin, W. F.; Herbst, J. H.; Rama, S. M.; Malow, R. M.; Purcell, D. W.; Wolitski, R. J. | yes | no | no | no |  | no |
| 17937817 | A novel emergency department based prevention intervention program for people living with HIV: evaluation of early experiences | BMC Health Serv Res | Lyons, M. S.; Raab, D. L.; Lindsell, C. J.; Trott, A. T.; Fichtenbaum, C. J. | yes | yes | yes | no |  | no |
| 106118440. Language: | A randomized trial of educational materials, pillboxes, and mailings to improve adherence with antiretroviral therapy in an inner city HIV clinic | Journal of Clinical Outcomes Management | Levin, T. R.; Klibanov, O. M.; Axelrod, P.; van den Berg-Wolf, M.; Finley, G. L.; Gray, A.; Holdsworth, C.; Moyer, D. V.; Tedaldi, E. M.; Samuel, R. | yes | no | no | no |  | no |
| 105505802. Language: | An adherence-focused case management intervention for HIV-positive patients in a public care setting | Journal of HIV/AIDS & Social Services | Wohl, A. R.; Garland, W. H.; Witt, M. D.; Valencia, R.; Boger, A.; Squires, K.; Kovacs, A.; Larsen, R.; Hader, S.; Anthony, M.; Frye, D.; Weidle, P. J. | yes | yes | no | no |  | no |
| 104530127. Language: | What HIV/AIDS Case Management Approaches Bring About Positive Client Outcomes? Results from ConnectHIV | Journal of HIV/AIDS & Social Services | Rogers, Susan J.; Corcoran, Caitlin L.; Hamdallah, Myriam; Little, Stacey | yes | no | no | no |  | no |
| 16785217 | Five year outcomes of a cohort of HIV-infected injection drug users in a primary care practice | J Addict Dis | Fingerhood, M.; Rastegar, D. A.; Jasinski, D. | yes | no | no | no |  | no |
| 20178030 | Integrated nested services: Delaware's experience treating minority substance abusers at risk for HIV or HIV positive | J Evid Based Soc Work | Dillard, D.; Bincsik, A. K.; Zebley, C.; Mongare, K.; Harrison, J.; Gerardi, K. E.; Parcher, D. W. | yes | yes | no | no |  | no |
| 20387984 | Project ROADMAP: Reeducating Older Adults in Maintaining AIDS Prevention: a secondary intervention for older HIV-positive adults | AIDS Educ Prev | Illa, L.; Echenique, M.; Jean, G. S.; Bustamante-Avellaneda, V.; Metsch, L.; Mendez-Mulet, L.; Eisdorfer, C.; Sanchez-Martinez, M. | yes | no | no | no |  | no |
| 17364394 | The acceptability of a directly-administered antiretroviral therapy (DAART) intervention among patients in public HIV clinics in Los Angeles, California | AIDS Care | Garland, W. H.; Wohl, A. R.; Valencia, R.; Witt, M. D.; Squires, K.; Kovacs, A.; Larsen, R.; Potterat, N.; Anthony, M. N.; Hader, S.; Weidle, P. J. | yes | no | no | no |  | no |
| 25090366 | The moderating role of sexual identity in group teletherapy for adults aging with HIV | Behav Med | Heckman, B. D.; Lovejoy, T. I.; Heckman, T. G.; Anderson, T.; Grimes, T.; Sutton, M.; Bianco, J. A. | yes | no | no | no |  | no |
| 25216879 | Impact of HIV-specialized pharmacies on adherence to medications for comorbid conditions | J Am Pharm Assoc (2003) | DuChane, J.; Clark, B.; Hou, J.; Fitzner, K.; Pietrandoni, G.; Duncan, I. | yes | no | yes | no |  | no |
| 26068720 | Outcomes of a Clinic-Based Surveillance-Informed Intervention to Relink Patients to HIV Care | J Acquir Immune Defic Syndr | Bove, J. M. | yes | no | yes | no |  | no |
| 2015-50012-024 | HIV-risk related attitudes and behaviors among older impoverished women living in Puerto Rico | Journal of Immigrant and Minority Health | Norman, Lisa R. | yes | no | no | no |  | no |
| 15750396 | Efficacy of a brief case management intervention to link recently diagnosed HIV-infected persons to care | Aids | Gardner, L. I.; Metsch, L. R.; Anderson-Mahoney, P.; Loughlin, A. M.; del Rio, C.; Strathdee, S.; Sansom, S. L.; Siegal, H. A.; Greenberg, A. E.; Holmberg, S. D. | yes | no | no | no |  | no |
| 17587172 | Supporting Positive Living and Sexual Health (SPLASH): a clinician and behavioral counselor risk-reduction intervention in a university-based HIV clinic | AIDS Behav | Zuniga, M. L.; Baldwin, H.; Uhler, D.; Brennan, J.; Olshefsky, A. M.; Oliver, E.; Mathews, W. C. | yes | no | no | no |  | no |
| 22107879 | Attitudes of methadone program staff toward provision of harm-reduction and other services | J Addict Med | Deren, S.; Kang, S. Y.; Mino, M.; Seewald, R. M. | yes | no | no | no |  | no |
| 23934269 | The LIVE Network: a music-based messaging program to promote ART adherence self-management | AIDS Behav | Holstad, M. M.; Ofotokun, I.; Higgins, M.; Logwood, S. | yes | no | no | no |  | no |
| 18922991 | Spirituality, social support, and survival in hemodialysis patients | Clin J Am Soc Nephrol | Spinale, J.; Cohen, S. D.; Khetpal, P.; Peterson, R. A.; Clougherty, B.; Puchalski, C. M.; Patel, S. S.; Kimmel, P. L. | yes | no | no | no |  | no |
| 17133193 | Cost-effectiveness of an intervention to improve adherence to antiretroviral therapy in HIV-infected patients | J Acquir Immune Defic Syndr | Freedberg, K. A.; Hirschhorn, L. R.; Schackman, B. R.; Wolf, L. L.; Martin, L. A.; Weinstein, M. C.; Goldin, S.; Paltiel, A. D.; Katz, C.; Goldie, S. J.; Losina, E. | yes | no | no | no |  | no |
| 17563286 | 'Getting me back on track': the role of outreach interventions in engaging and retaining people living with HIV/AIDS in medical care | AIDS Patient Care STDS | Rajabiun, S.; Mallinson, R. K.; McCoy, K.; Coleman, S.; Drainoni, M. L.; Rebholz, C.; Holbert, T. | yes | yes | yes | no |  | no |
| 19050396 | A controlled study of the effectiveness of public health HIV partner notification services | Aids | Golden, M. R.; Dombrowski, J. C.; Wood, R. W.; Fleming, M.; Harrington, R. D. | yes | no | no | no |  | no |
| 106428431. Language: | A longitudinal evaluation of a social support model of medication adherence among HIV-positive men and women on antiretroviral therapy | Health Psychology | Simoni, J. M.; Frick, P. A.; Huang, B. | yes | yes | no | no |  | no |
| 18058396 | A model of integrated primary care for HIV-positive patients with underlying substance use and mental illness | AIDS Care | Zaller, N.; Gillani, F. S.; Rich, J. D. | yes | yes | no | no |  | no |
| 25695849 | A pilot feasibility and acceptability study of yoga/meditation on the quality of life and markers of stress in persons living with HIV who also use crack cocaine | J Altern Complement Med | Agarwal, R. P.; Kumar, A.; Lewis, J. E. | yes | no | no | no |  | no |
| 23812892 | A preliminary RCT of CBT-AD for adherence and depression among HIV-positive Latinos on the U.S.-Mexico border: the Nuevo Dia study | AIDS Behav | Simoni, J. M.; Wiebe, J. S.; Sauceda, J. A.; Huh, D.; Sanchez, G.; Longoria, V.; Andres Bedoya, C.; Safren, S. A. | yes | no | no | no |  | no |
| 17828761 | A preliminary study of spiritual self-schema (3-S(+)) therapy for reducing impulsivity in HIV-positive drug users | J Clin Psychol | Margolin, A.; Schuman-Olivier, Z.; Beitel, M.; Arnold, R. M.; Fulwiler, C. E.; Avants, S. K. | yes | no | no | no |  | no |
| 20857188 | A randomized clinical trial of a coping improvement group intervention for HIV-infected older adults | J Behav Med | Heckman, T. G.; Sikkema, K. J.; Hansen, N.; Kochman, A.; Heh, V.; Neufeld, S. | yes | no | no | no |  | no |
| 18540736 | A randomized clinical trial of alternative stress management interventions in persons with HIV infection | J Consult Clin Psychol | McCain, N. L.; Gray, D. P.; Elswick, R. K.; Robins, J. W.; Tuck, I.; Walter, J. M.; Rausch, S. M.; Ketchum, J. M. | yes | no | no | no |  | no |
| 17589194 | A randomized clinical trial of community-based directly observed therapy as an adherence intervention for HAART among substance users | Aids | Macalino, G. E.; Hogan, J. W.; Mitty, J. A.; Bazerman, L. B.; Delong, A. K.; Loewenthal, H.; Caliendo, A. M.; Flanigan, T. P. | yes | no | no | no |  | no |
| 23430708 | A randomized clinical trial of smoking cessation treatments provided in HIV clinical care settings | Nicotine Tob Res | Humfleet, G. L.; Hall, S. M.; Delucchi, K. L.; Dilley, J. W. | yes | no | no | no |  | no |
| 22732470 | A randomized controlled trial of a tailored group smoking cessation intervention for HIV-infected smokers | J Acquir Immune Defic Syndr | Moadel, A. B.; Bernstein, S. L.; Mermelstein, R. J.; Arnsten, J. H.; Dolce, E. H.; Shuter, J. | yes | no | no | no |  | no |
| 16238507 | A randomized controlled trial of meditation and massage effects on quality of life in people with late-stage disease: a pilot study | J Palliat Med | Williams, A. L.; Selwyn, P. A.; Liberti, L.; Molde, S.; Njike, V. Y.; McCorkle, R.; Zelterman, D.; Katz, D. L. | yes | no | no | no |  | no |
| 16170757 | A randomized study of serial telephone call support to increase adherence and thereby improve virologic outcome in persons initiating antiretroviral therapy | J Infect Dis | Collier, A. C.; Ribaudo, H.; Mukherjee, A. L.; Feinberg, J.; Fischl, M. A.; Chesney, M. | yes | no | no | no |  | no |
| 16673835 | A randomized trial of the efficacy of group therapy in changing viral load and CD4 counts in individuals living with HIV infection | Int J Psychiatry Med | Belanoff, J. K.; Sund, B.; Koopman, C.; Blasey, C.; Flamm, J.; Schatzberg, A. F.; Spiegel, D. | yes | no | no | no |  | no |
| 16827627 | A telephone-delivered coping improvement group intervention for middle-aged and older adults living with HIV/AIDS | Ann Behav Med | Heckman, T. G.; Barcikowski, R.; Ogles, B.; Suhr, J.; Carlson, B.; Holroyd, K.; Garske, J. | yes | no | no | no |  | no |
| 18263847 | Acceptance of a nutrition curriculum for HIV-positive Latinos Living on the U.S.-Mexico border | J Transcult Nurs | Torres, K.; Zive, M. M.; Scolari, R.; Olshefsky, A.; Zuniga, M. L. | yes | no | no | no |  | no |
| 17546496 | Access to housing as a structural intervention for homeless and unstably housed people living with HIV: rationale, methods, and implementation of the housing and health study | AIDS Behav | Kidder, D. P.; Wolitski, R. J.; Royal, S.; Aidala, A.; Courtenay-Quirk, C.; Holtgrave, D. R.; Harre, D.; Sumartojo, E.; Stall, R. | yes | yes | no | no |  | no |
| 109837242. Language: | Achieving Core Indicators for HIV Clinical Care Among New Patients at an Urban HIV Clinic | AIDS Patient Care & STDs | Greer, Gillian A.; Tamhane, Ashutosh; Malhotra, Rakhi; Burkholder, Greer A.; Mugavero, Michael J.; Raper, James L.; Zinski, Anne | yes | yes | yes | no |  | no |
| 23356569 | African-Americans' perceptions of health care provider cultural competence that promote HIV medical self-care and antiretroviral medication adherence | AIDS Care | Gaston, G. B. | yes | yes | no | no |  | no |
| 21443312 | Agreement between prospective interactive voice response telephone reporting and structured recall reports of risk behaviors in rural substance users living with HIV/AIDS | Psychol Addict Behav | Simpson, C. A.; Xie, L.; Blum, E. R.; Tucker, J. A. | yes | no | no | no |  | no |
| 17333382 | Altering key characteristics of a disseminated effective behavioral intervention for HIV positive adults: the 'healthy relationships' experience | J Prim Prev | Kalichman, S. C.; Cherry, C.; White, D.; Pope, H.; Cain, D.; Kalichman, M. | yes | no | no | no |  | no |
| 26181705 | An Algorithm Approach to Determining Smoking Cessation Treatment for Persons Living With HIV/AIDS: Results of a Pilot Trial | J Acquir Immune Defic Syndr | Cropsey, K. L.; Jardin, B. F.; Burkholder, G. A.; Clark, C. B.; Raper, J. L.; Saag, M. S. | yes | no | no | no |  | no |
| 21244204 | An evidence-based group coping intervention for women living with HIV and history of childhood sexual abuse | Int J Group Psychother | Puffer, E. S.; Kochman, A.; Hansen, N. B.; Sikkema, K. J. | yes | no | no | no |  | no |
| 21191643 | An interrupted time series evaluation of a hepatitis C intervention for persons with HIV | AIDS Behav | Proeschold-Bell, R. J.; Hoeppner, B.; Taylor, B.; Cohen, S.; Blouin, R.; Stringfield, B.; Muir, A. J. | yes | no | no | no |  | no |
| 22792882 | Appearance concerns and psychological distress among HIV-infected individuals with injection drug use histories: prospective analyses | AIDS Patient Care STDS | Blashill, A. J.; Gordon, J. R.; Safren, S. A. | yes | no | no | no |  | no |
| 21208772 | Applying the theory of planned behavior to explore HAART adherence among HIV-positive immigrant Latinos: elicitation interview results | Patient Educ Couns | Vissman, A. T.; Hergenrather, K. C.; Rojas, G.; Langdon, S. E.; Wilkin, A. M.; Rhodes, S. D. | yes | yes | no | no |  | no |
| 103793987. Language: | Behavioral Interventions to Reduce Sexual Risk Behavior in Adults with HIV/AIDS Receiving HIV Care: A Systematic Review | AIDS Patient Care & STDs | Laisaar, Kaja-Triin; Raag, Mait; Rosenthal, Marika; UuskÃ¼la, Anneli | yes | no | no | no |  | no |
| 16979403 | Body composition and metabolic effects of a diet and exercise weight loss regimen on obese, HIV-infected women | Metabolism | Engelson, E. S.; Agin, D.; Kenya, S.; Werber-Zion, G.; Luty, B.; Albu, J. B.; Kotler, D. P. | yes | no | no | no |  | no |
| 22965693 | Bridging the digital divide in HIV care: a pilot study of an iPod personal health record | J Int Assoc Provid AIDS Care | Luque, A. E.; Corales, R.; Fowler, R. J.; DiMarco, J.; van Keken, A.; Winters, P.; Keefer, M. C.; Fiscella, K. | yes | no | no | no |  | no |
| 21457056 | Brief behavioral self-regulation counseling for HIV treatment adherence delivered by cell phone: an initial test of concept trial | AIDS Patient Care STDS | Kalichman, S. C.; Kalichman, M. O.; Cherry, C.; Swetzes, C.; Amaral, C. M.; White, D.; Jones, M.; Grebler, T.; Eaton, L. | yes | no | no | no |  | no |
| 18285714 | Brief strengths-based case management promotes entry into HIV medical care: results of the antiretroviral treatment access study-II | J Acquir Immune Defic Syndr | Craw, J. A.; Gardner, L. I.; Marks, G.; Rapp, R. C.; Bosshart, J.; Duffus, W. A.; Rossman, A.; Coughlin, S. L.; Gruber, D.; Safford, L. A.; Overton, J.; Schmitt, K. | yes | yes | yes | no |  | no |
| 21375430 | Buffering effects of general and medication-specific social support on the association between substance use and HIV medication adherence | AIDS Patient Care STDS | Lehavot, K.; Huh, D.; Walters, K. L.; King, K. M.; Andrasik, M. P.; Simoni, J. M. | yes | yes | no | no |  | no |
| 22770948 | Can lay health workers promote better medical self-management by persons living with HIV? An evaluation of the Positive Choices program | Patient Educ Couns | Roth, A. M.; Holmes, A. M.; Stump, T. E.; Aalsma, M. C.; Ackermann, R. T.; Carney, T. S.; Katz, B. P.; Kesterson, J.; Erdman, S. M.; Balt, C. A.; Inui, T. S. | yes | no | no | no |  | no |
| 18672562 | Case management and health-related quality of life outcomes in a national sample of persons with HIV/AIDS | J Natl Med Assoc | Cunningham, W. E.; Wong, M.; Hays, R. D. | yes | yes | yes | no |  | no |
| 103973292. Language: | Cell Phone Utilization Among Foreign-Born Latinos: A Promising Tool for Dissemination of Health and HIV Information | Journal of Immigrant & Minority Health | Leite, Lorena; Buresh, Megan; Rios, Naomi; Conley, Anna; Flys, Tamara; Page, Kathleen | yes | no | no | no |  | no |
| 25559605 | Clinic-wide intervention lowers financial risk and improves revenue to HIV clinics through fewer missed primary care visits | J Acquir Immune Defic Syndr | Gardner, L. I.; Marks, G.; Wilson, T. E.; Giordano, T. P.; Sullivan, M.; Raper, J. L.; Rodriguez, A. E.; Keruly, J.; Malitz, F. | yes | no | yes | no |  | no |
| 16340472 | Clinician-delivered intervention during routine clinical care reduces unprotected sexual behavior among HIV-infected patients | J Acquir Immune Defic Syndr | Fisher, J. D.; Fisher, W. A.; Cornman, D. H.; Amico, R. K.; Bryan, A.; Friedland, G. H. | yes | no | no | no |  | no |
| 16909323 | Co-location of health care for adults with serious mental illness and HIV infection | Community Ment Health J | Sullivan, G.; Kanouse, D.; Young, A. S.; Han, X.; Perlman, J.; Koegel, P. | yes | no | no | no |  | no |
| 104568605. Language: | Cognitive Behavioral HIV Risk Reduction in Those Receiving Psychiatric Treatment: A Clinical Trial | AIDS & Behavior | Malow, Robert; McMahon, Robert; DÃ©vieux, Jessy; Rosenberg, Rhonda; Frankel, Anne; Bryant, Vaughn; Lerner, Brenda; Miguez, Maria | yes | yes | no | no |  | no |
| 16816559 | Cognitive-behavioral intervention to enhance adherence to antiretroviral therapy: a randomized controlled trial (CCTG 578) | Aids | Wagner, G. J.; Kanouse, D. E.; Golinelli, D.; Miller, L. G.; Daar, E. S.; Witt, M. D.; Diamond, C.; Tilles, J. G.; Kemper, C. A.; Larsen, R.; Goicoechea, M.; Haubrich, R. H. | yes | no | yes | no |  | no |
| 24271348 | Combined HIV prevention, the New York City condom distribution program, and the evolution of safer sex behavior among persons who inject drugs in New York City | AIDS Behav | Des Jarlais, D. C.; Arasteh, K.; McKnight, C.; Feelemyer, J.; Hagan, H.; Cooper, H. L.; Perlman, D. C. | yes | no | no | no |  | no |
| 25567825 | Comparable sustained virologic suppression between community- and academic-based HIV care settings | J Am Board Fam Med | Chu, C.; Heo, M.; Peshansky, A.; Umanski, G.; Meissner, P.; Voss, C.; Selwyn, P. A. | yes | no | no | no |  | no |
| 22098257 | Comparative cost analysis of housing and case management program for chronically ill homeless adults compared to usual care | Health Serv Res | Basu, A.; Kee, R.; Buchanan, D.; Sadowski, L. S. | yes | no | no | no |  | no |
| 21452051 | Computer-based intervention in HIV clinical care setting improves antiretroviral adherence: the LifeWindows Project | AIDS Behav | Fisher, J. D.; Amico, K. R.; Fisher, W. A.; Cornman, D. H.; Shuper, P. A.; Trayling, C.; Redding, C.; Barta, W.; Lemieux, A. F.; Altice, F. L.; Dieckhaus, K.; Friedland, G. | yes | no | no | no |  | no |
| 24384803 | Computerized counseling reduces HIV-1 viral load and sexual transmission risk: findings from a randomized controlled trial | J Acquir Immune Defic Syndr | Kurth, A. E.; Spielberg, F.; Cleland, C. M.; Lambdin, B.; Bangsberg, D. R.; Frick, P. A.; Severynen, A. O.; Clausen, M.; Norman, R. G.; Lockhart, D.; Simoni, J. M.; Holmes, K. K. | yes | no | no | no |  | no |
| 22117123 | Computerized stress management training for HIV+ women: a pilot intervention study | AIDS Care | Brown, J. L.; Vanable, P. A.; Carey, M. P.; Elin, L. | yes | no | no | no |  | no |
| 23138877 | Cost analysis of enhancing linkages to HIV care following jail: a cost-effective intervention | AIDS Behav | Spaulding, A. C.; Pinkerton, S. D.; Superak, H.; Cunningham, M. J.; Resch, S.; Jordan, A. O.; Yang, Z. | yes | no | no | no |  | no |
| 24287787 | Current cigarette smoking among HIV-positive current and former drug users: associations with individual and social characteristics | AIDS Behav | Pacek, L. R.; Latkin, C.; Crum, R. M.; Stuart, E. A.; Knowlton, A. R. | yes | yes | no | no |  | no |
| 19645619 | Demographic, psychological, and behavioral modifiers of the Antiretroviral Treatment Access Study (ARTAS) intervention | AIDS Patient Care STDS | Gardner, L. I.; Marks, G.; Craw, J.; Metsch, L.; Strathdee, S.; Anderson-Mahoney, P.; del Rio, C. | yes | no | no | no |  | no |
| 104461961. Language: | Development and Validation of a Measure for Intention to Adhere to HIV Treatment | AIDS Patient Care & STDs | Nelsen, Andrea; Trautner, Barbara W.; Petersen, Nancy J.; Gupta, Sunita; Rodriguez-Barradas, Maria; Giordano, Thomas P.; Naik, Aanand D. | yes | no | no | no |  | no |
| 23780395 | Differences in human immunodeficiency virus care and treatment among subpopulations in the United States | JAMA Intern Med | Hall, H. I.; Frazier, E. L.; Rhodes, P.; Holtgrave, D. R.; Furlow-Parmley, C.; Tang, T.; Gray, K. M.; Cohen, S. M.; Mermin, J.; Skarbinski, J. | yes | no | no | no |  | no |
| 18193497 | Directly administered antiretroviral therapy for HIV-infected drug users does not have an impact on antiretroviral resistance: results from a randomized controlled trial | J Acquir Immune Defic Syndr | Maru, D. S.; Kozal, M. J.; Bruce, R. D.; Springer, S. A.; Altice, F. L. | yes | no | no | no |  | no |
| 23874575 | Directly administered antiretroviral therapy for HIV-infected individuals in opioid treatment programs: results from a randomized clinical trial | PLoS One | Lucas, G. M.; Mullen, B. A.; Galai, N.; Moore, R. D.; Cook, K.; McCaul, M. E.; Glass, S.; Oursler, K. K.; Rand, C. | yes | no | no | no |  | no |
| 21885212 | Directly observed antiretroviral therapy eliminates adverse effects of active drug use on adherence | Drug Alcohol Depend | Nahvi, S.; Litwin, A. H.; Heo, M.; Berg, K. M.; Li, X.; Arnsten, J. H. | yes | yes | yes | no |  | no |
| 24286967 | Drinking motives as prospective predictors of outcome in an intervention trial with heavily drinking HIV patients | Drug Alcohol Depend | Elliott, J. C.; Aharonovich, E.; O'Leary, A.; Wainberg, M.; Hasin, D. S. | yes | no | no | no |  | no |
| 21937921 | Early retention in HIV care and viral load suppression: implications for a test and treat approach to HIV prevention | J Acquir Immune Defic Syndr | Mugavero, M. J.; Amico, K. R.; Westfall, A. O.; Crane, H. M.; Zinski, A.; Willig, J. H.; Dombrowski, J. C.; Norton, W. E.; Raper, J. L.; Kitahata, M. M.; Saag, M. S. | yes | no | yes | no |  | no |
| 22983536 | Effect of a clinic-wide social marketing campaign to improve adherence to antiretroviral therapy for HIV infection | AIDS Behav | Giordano, T. P.; Rodriguez, S.; Zhang, H.; Kallen, M. A.; Jibaja-Weiss, M.; Buscher, A. L.; Arya, M.; Suarez-Almazor, M. E.; Ross, M. | yes | no | no | no |  | no |
| 17224844 | Effect of clinical pharmacists on utilization of and clinical response to antiretroviral therapy | J Acquir Immune Defic Syndr | Horberg, M. A.; Hurley, L. B.; Silverberg, M. J.; Kinsman, C. J.; Quesenberry, C. P. | yes | no | no | no |  | no |
| 19054631 | Effect of incentives for medication adherence on health care use and costs in methadone patients with HIV | Drug Alcohol Depend | Barnett, P. G.; Sorensen, J. L.; Wong, W.; Haug, N. A.; Hall, S. M. | yes | no | no | no |  | no |
| 24237482 | Effectiveness of healthy relationships video-group-A videoconferencing group intervention for women living with HIV: preliminary findings from a randomized controlled trial | Telemed J E Health | Marhefka, S. L.; Buhi, E. R.; Baldwin, J.; Chen, H.; Johnson, A.; Lynn, V.; Glueckauf, R. | yes | no | no | no |  | no |
| 103926668. Language: | Effects of a Brief Case Management Intervention Linking People With HIV to Oral Health Care: Project SMILE | American Journal of Public Health | Metsch, Lisa R.; Pereyra, Margaret; Messinger, Shari; Jeanty, Yves; Parish, Carrigan; Valverde, Eduardo; Cardenas, Gabriel; Boza, Henry; Tomar, Scott | yes | no | no | no |  | no |
| 18176319 | Effects of a coping intervention on transmission risk behavior among people living with HIV/AIDS and a history of childhood sexual abuse | J Acquir Immune Defic Syndr | Sikkema, K. J.; Wilson, P. A.; Hansen, N. B.; Kochman, A.; Neufeld, S.; Ghebremichael, M. S.; Kershaw, T. | yes | no | no | no |  | no |
| 19154859 | Effects of faith/assurance on cortisol levels are enhanced by a spiritual mantram intervention in adults with HIV: a randomized trial | J Psychosom Res | Bormann, J. E.; Aschbacher, K.; Wetherell, J. L.; Roesch, S.; Redwine, L. | yes | no | no | no |  | no |
| 16847590 | Effects of spiritual mantram repetition on HIV outcomes: a randomized controlled trial | J Behav Med | Bormann, J. E.; Gifford, A. L.; Shively, M.; Smith, T. L.; Redwine, L.; Kelly, A.; Becker, S.; Gershwin, M.; Bone, P.; Belding, W. | yes | no | no | no |  | no |
| 20135214 | Efficacy of a single-session HIV prevention intervention for black women: a group randomized controlled trial | AIDS Behav | Diallo, D. D.; Moore, T. W.; Ngalame, P. M.; White, L. D.; Herbst, J. H.; Painter, T. M. | yes | no | no | no |  | no |
| 25211714 | Efficacy of an adapted HIV and sexually transmitted infection prevention intervention for incarcerated women: a randomized controlled trial | Am J Public Health | Fogel, C. I.; Crandell, J. L.; Neevel, A. M.; Parker, S. D.; Carry, M.; White, B. L.; Fasula, A. M.; Herbst, J. H.; Gelaude, D. J. | yes | yes | no | no |  | no |
| 19996978 | Efficacy of brief interventions in clinical care settings for persons living with HIV | J Acquir Immune Defic Syndr | Lightfoot, M.; Rotheram-Borus, M. J.; Comulada, W. S.; Reddy, V. S.; Duan, N. | yes | no | no | no |  | no |
| 104998713. Language: | Engaging hiv-positive individuals in specialized care from an urban emergency department | AIDS Patient Care & STDs | Leider, J.; Fettig, J.; Calderon, Y. | yes | yes | no | no |  | no |
| 23494224 | Enhancing acceptability and use of sexual barrier products among HIV concordant and discordant couples | AIDS Behav | Jones, D.; Kashy, D.; Villar-Loubet, O.; Weiss, S. | yes | no | no | no |  | no |
| 0 | Establishing an HIV Screening Program Led by Staff Nurses in a County Jail | Journal of Public Health Management & Practice | Spaulding | yes | yes | no | no |  | no |
| 19125548 | Evaluation of the first year of a pilot program in community pharmacy: HIV/AIDS medication therapy management for Medi-Cal beneficiaries | J Manag Care Pharm | Hirsch, J. D.; Rosenquist, A.; Best, B. M.; Miller, T. A.; Gilmer, T. P. | yes | no | yes | no |  | no |
| 23382948 | Examining the link between patient satisfaction and adherence to HIV care: a structural equation model | PLoS One | Dang, B. N.; Westbrook, R. A.; Black, W. C.; Rodriguez-Barradas, M. C.; Giordano, T. P. | yes | no | no | no |  | no |
| 17786561 | Experiences of social stigma and implications for healthcare among a diverse population of HIV positive adults | J Urban Health | Sayles, J. N.; Ryan, G. W.; Silver, J. S.; Sarkisian, C. A.; Cunningham, W. E. | yes | yes | yes | no |  | no |
| 16364566 | Facilitating entry into drug treatment among injection drug users referred from a needle exchange program: Results from a community-based behavioral intervention trial | Drug Alcohol Depend | Strathdee, S. A.; Ricketts, E. P.; Huettner, S.; Cornelius, L.; Bishai, D.; Havens, J. R.; Beilenson, P.; Rapp, C.; Lloyd, J. J.; Latkin, C. A. | yes | yes | no | no |  | no |
| 21165692 | Group motivational interviewing to promote adherence to antiretroviral medications and risk reduction behaviors in HIV infected women | AIDS Behav | Holstad, M. M.; DiIorio, C.; Kelley, M. E.; Resnicow, K.; Sharma, S. | yes | yes | no | no |  | no |
| 22854158 | Health information exchange interventions can enhance quality and continuity of HIV care | Int J Med Inform | Shade, S. B.; Chakravarty, D.; Koester, K. A.; Steward, W. T.; Myers, J. J. | yes | no | no | no |  | no |
| 18569359 | Health literacy of HIV-positive individuals enrolled in an outreach intervention: results of a cross-site analysis | J Health Commun | Drainoni, M. L.; Rajabiun, S.; Rumptz, M.; Welles, S. L.; Relf, M.; Rebholz, C.; Holmes, L.; Dyl, A.; Lovejoy, T.; Dekker, D.; Frye, A. | yes | no | no | no |  | no |
| 103872124. Language: | Health Outcomes for HIV-Infected Persons Released From the New York City Jail System With a Transitional Care-Coordination Plan | American Journal of Public Health | Teixeira, Paul A.; Jordan, Alison O.; Zaller, Nicolas; Shah, Dipal; Venters, Homer | yes | no | no | no |  | no |
| 24533631 | HealthCall for the smartphone: technology enhancement of brief intervention in HIV alcohol dependent patients | Addict Sci Clin Pract | Hasin, D. S.; Aharonovich, E.; Greenstein, E. | yes | no | no | no |  | no |
| 22970975 | Helping clinicians deliver consistent HIV prevention counseling to their HIV-infected patients | AIDS Care | Myers, J. J.; Kang Dufour, M. S.; Koester, K. A.; Rose, C. D.; Shade, S. B.; Maiorana, A.; Morin, S. F. | yes | yes | no | no |  | no |
| 23148715 | Helping people with HIV/AIDS return to work: a randomized clinical trial | Rehabil Psychol | Martin, D. J.; Chernoff, R. A.; Buitron, M.; Comulada, W. S.; Liang, L. J.; Wong, F. L. | yes | yes | no | no |  | no |
| 25225233 | High rates of retention and viral suppression in the US HIV safety net system: HIV care continuum in the Ryan White HIV/AIDS Program, 2011 | Clin Infect Dis | Doshi, R. K.; Milberg, J.; Isenberg, D.; Matthews, T.; Malitz, F.; Matosky, M.; Trent-Adams, S.; Parham Hopson, D.; Cheever, L. W. | yes | no | yes | no |  | no |
| 20827218 | HIV intervention for providers study: a randomized controlled trial of a clinician-delivered HIV risk-reduction intervention for HIV-positive people | J Acquir Immune Defic Syndr | Rose, C. D.; Courtenay-Quirk, C.; Knight, K.; Shade, S. B.; Vittinghoff, E.; Gomez, C.; Lum, P. J.; Bacon, O.; Colfax, G. | yes | no | no | no |  | no |
| 21214376 | HIV quality performance measures in a large integrated health care system | AIDS Patient Care STDS | Horberg, M.; Hurley, L.; Towner, W.; Gambatese, R.; Klein, D.; Antoniskis, D.; Weinberg, W.; Kadlecik, P.; Remmers, C.; Dobrinich, R.; Quesenberry, C.; Silverberg, M.; Johnson, M. | yes | no | no | no |  | no |
| 21341962 | HIV-disclosure, social support, and depression among HIV-infected African American women living in the rural southeastern United States | AIDS Educ Prev | Vyavaharkar, M.; Moneyham, L.; Corwin, S.; Tavakoli, A.; Saunders, R.; Annang, L. | yes | yes | no | no |  | no |
| 20824549 | HIV-infected patients and treatment outcomes: an equivalence study of community-located, primary care-based HIV treatment vs. hospital-based specialty care in the Bronx, New York | AIDS Care | Chu, C.; Umanski, G.; Blank, A.; Grossberg, R.; Selwyn, P. A. | yes | no | yes | no |  | no |
| 20401768 | How to improve the quality of a disease management program for HIV-infected patients using a computerized data system. The Saint-Antoine Orchestra program | AIDS Care | Fonquernie, F.; Lacombe, K.; Vincensini, J. P.; Boccara, F.; Clozel, S.; Ayouch Boda, A.; Bollens, D.; Campa, P.; Pacanowski, J.; Meynard, J. L.; Meyohas, M. C.; Girard, P. M. | yes | no | no | no |  | no |
| 22298930 | Impact of a New York City Health Department initiative to expand HIV partner services outside STD clinics | Public Health Rep | Udeagu, C. C.; Shah, D.; Shepard, C. W.; Bocour, A.; Guiterrez, R.; Begier, E. M. | yes | no | no | no |  | no |
| 22272732 | Impact of adherence counseling dose on antiretroviral adherence and HIV viral load among HIV-infected methadone maintained drug users | AIDS Care | Cooperman, N. A.; Heo, M.; Berg, K. M.; Li, X.; Litwin, A. H.; Nahvi, S.; Arnsten, J. H. | yes | no | no | no |  | no |
| 22860900 | Impact of HIV-specialized pharmacies on adherence and persistence with antiretroviral therapy | AIDS Patient Care STDS | Murphy, P.; Cocohoba, J.; Tang, A.; Pietrandoni, G.; Hou, J.; Guglielmo, B. J. | yes | no | no | no |  | no |
| 18627280 | Implementation and evaluation of a clinic-based behavioral intervention: positive steps for patients with HIV | AIDS Patient Care STDS | Gardner, L. I.; Marks, G.; O'Daniels, C. M.; Wilson, T. E.; Golin, C.; Wright, J.; Quinlivan, E. B.; Bradley-Springer, L.; Thompson, M.; Raffanti, S.; Thrun, M. | yes | no | no | no |  | no |
| 23023860 | Improved antiretroviral refill adherence in HIV-focused community pharmacies | J Am Pharm Assoc (2003) | Cocohoba, J. M.; Murphy, P.; Pietrandoni, G.; Guglielmo, B. J. | yes | yes | no | no |  | no |
| 19243231 | Improved prevention counseling by HIV care providers in a multisite, clinic-based intervention: Positive STEPs | AIDS Educ Prev | Thrun, M.; Cook, P. F.; Bradley-Springer, L. A.; Gardner, L.; Marks, G.; Wright, J.; Wilson, T. E.; Quinlivan, E. B.; O'Daniels, C.; Raffanti, S.; Thompson, M.; Golin, C. | yes | no | no | no |  | no |
| 17428189 | Improvement of psychosocial adjustment to HIV-1 infection through a cognitive-behavioral oriented group psychotherapy program: a pilot study | AIDS Patient Care STDS | Rousaud, A.; Blanch, J.; Hautzinger, M.; De Lazzari, E.; Peri, J. M.; Puig, O.; Martinez, E.; Masana, G.; De Pablo, J.; Gatell, J. M. | yes | yes | no | no |  | no |
| 25301208 | Improvements in HIV care engagement and viral load suppression following enrollment in a comprehensive HIV care coordination program | Clin Infect Dis | Irvine, M. K.; Chamberlin, S. A.; Robbins, R. S.; Myers, J. E.; Braunstein, S. L.; Mitts, B. J.; Harriman, G. A.; Laraque, F.; Nash, D. | yes | yes | yes | no |  | no |
| 24625234 | Improving cervical cancer screening rates in an urban HIV clinic | AIDS Care | Cross, S. L.; Suharwardy, S. H.; Bodavula, P.; Schechtman, K.; Overton, E. T.; Onen, N. F.; Lane, M. A. | yes | no | no | no |  | no |
| 20922510 | Improving coping skills for self-management of treatment side effects can reduce antiretroviral medication nonadherence among people living with HIV | Ann Behav Med | Johnson, M. O.; Dilworth, S. E.; Taylor, J. M.; Neilands, T. B. | yes | no | no | no |  | no |
| 16770694 | Improving dietary habits in disadvantaged women with HIV/AIDS: the SMART/EST women's project | AIDS Behav | Segal-Isaacson, C. J.; Tobin, J. N.; Weiss, S. M.; Brondolo, E.; Vaughn, A.; Wang, C.; Camille, J.; Gousse, Y.; Ishii, M.; Jones, D.; Laperriere, A.; Lydston, D.; Schneiderman, N.; Ironson, G. | yes | no | no | no |  | no |
| 23265919 | Improving sleep hygiene behavior in adults living with HIV/AIDS: a randomized control pilot study of the SystemCHANGE(TM)-HIV intervention | Appl Nurs Res | Webel, A. R.; Moore, S. M.; Hanson, J. E.; Patel, S. R.; Schmotzer, B.; Salata, R. A. | yes | no | no | no |  | no |
| 104288112. Language: | Improving sleep hygiene behavior in adults living with HIV/AIDS: a randomized control pilot study of the SystemCHANGEâ„¢-HIV intervention | Applied Nursing Research | Webel, Allison R.; Moore, Shirley M.; Hanson, Jan E.; Patel, Sanjay R.; Schmotzer, Brian; Salata, Robert A. | yes | yes | no | no |  | no |
| 19127438 | Increases in positive reappraisal coping during a group-based mantram intervention mediate sustained reductions in anger in HIV-positive persons | Int J Behav Med | Bormann, J. E.; Carrico, A. W. | yes | no | no | no |  | no |
| 22547872 | Increasing access to oral health care for people living with HIV/AIDS in the U.S.: baseline evaluation results of the Innovations in Oral Health Care Initiative | Public Health Rep | Fox, J. E.; Tobias, C. R.; Bachman, S. S.; Reznik, D. A.; Rajabiun, S.; Verdecias, N. | yes | yes | yes | no |  | no |
| 20675952 | Increasing HIV-related knowledge, communication, and testing intentions among Latinos: Protege tu Familia: Hazte la Prueba | J Health Care Poor Underserved | Rios-Ellis, B.; Espinoza, L.; Bird, M.; Garcia, M.; D'Anna, L. H.; Bellamy, L.; Scolari, R. | yes | no | no | no |  | no |
| 21042930 | Intensive case management before and after prison release is no more effective than comprehensive pre-release discharge planning in linking HIV-infected prisoners to care: a randomized trial | AIDS Behav | Wohl, D. A.; Scheyett, A.; Golin, C. E.; White, B.; Matuszewski, J.; Bowling, M.; Smith, P.; Duffin, F.; Rosen, D.; Kaplan, A.; Earp, J. | yes | no | no | no |  | no |
| 104418936. Language: | Interest in, Concerns About, and Preferences for Potential Video-Group Delivery of an Effective Behavioral Intervention Among Women Living With HIV | AIDS & Behavior | Marhefka, Stephanie; Fuhrmann, Hollie; Gilliam, Patricia; Lopez, Bernice; Baldwin, Julie | yes | no | no | no |  | no |
| 21890753 | Lack of sustained improvement in adherence or viral load following a directly observed antiretroviral therapy intervention | Clin Infect Dis | Berg, K. M.; Litwin, A. H.; Li, X.; Heo, M.; Arnsten, J. H. | yes | no | yes | no |  | no |
| 17139203 | Life changing words: women's responses to being diagnosed with HIV infection | ANS Adv Nurs Sci | Stevens, P. E.; Hildebrandt, E. | yes | yes | yes | no |  | no |
| 23224290 | Linkage to HIV care for jail detainees: findings from detention to the first 30 days after release | AIDS Behav | Booker, C. A.; Flygare, C. T.; Solomon, L.; Ball, S. W.; Pustell, M. R.; Bazerman, L. B.; Simon-Levine, D.; Teixeira, P. A.; Cruzado-Quinones, J.; Kling, R. N.; Frew, P. M.; Spaulding, A. C. | yes | no | no | no |  | no |
| 23982662 | Linkage, engagement, and viral suppression rates among HIV-infected persons receiving care at medical case management programs in Washington, DC | J Acquir Immune Defic Syndr | Willis, S.; Castel, A. D.; Ahmed, T.; Olejemeh, C.; Frison, L.; Kharfen, M. | yes | yes | no | no |  | no |
| 15989436 | Linking HIV-infected persons of color and injection drug users to HIV medical and other services: the California Bridge Project | AIDS Patient Care STDS | Molitor, F.; Kuenneth, C.; Waltermeyer, J.; Mendoza, M.; Aguirre, A.; Brockmann, K.; Crump, C. | yes | no | no | no |  | no |
| 23704120 | Long-term outcomes of a cell phone-delivered intervention for smokers living with HIV/AIDS | Clin Infect Dis | Gritz, E. R.; Danysh, H. E.; Fletcher, F. E.; Tami-Maury, I.; Fingeret, M. C.; King, R. M.; Arduino, R. C.; Vidrine, D. J. | yes | no | no | no |  | no |
| 23358784 | Managed problem solving for antiretroviral therapy adherence: a randomized trial | JAMA Intern Med | Gross, R.; Bellamy, S. L.; Chapman, J.; Han, X.; O'Duor, J.; Palmer, S. C.; Houts, P. S.; Coyne, J. C.; Strom, B. L. | yes | yes | no | no |  | no |
| 18677084 | Medical and support service utilization in a medical program targeting marginalized HIV-infected individuals | J Health Care Poor Underserved | Cunningham, C. O.; Sanchez, J. P.; Li, X.; Heller, D.; Sohler, N. L. | yes | yes | yes | no |  | no |
| 21925831 | Mindfulness-based stress reduction for HIV treatment side effects: a randomized, wait-list controlled trial | J Pain Symptom Manage | Duncan, L. G.; Moskowitz, J. T.; Neilands, T. B.; Dilworth, S. E.; Hecht, F. M.; Johnson, M. O. | yes | no | no | no |  | no |
| 19430305 | Most positive HIV western blot tests do not diagnose new cases in New York City: implications for HIV testing programs | J Acquir Immune Defic Syndr | Hanna, D. B.; Tsoi, B. W.; Begier, E. M. | yes | yes | no | no |  | no |
| 18077833 | Motivational interviewing and cognitive-behavioral intervention to improve HIV medication adherence among hazardous drinkers: a randomized controlled trial | J Acquir Immune Defic Syndr | Parsons, J. T.; Golub, S. A.; Rosof, E.; Holder, C. | yes | no | no | no |  | no |
| 22994175 | New Jersey's HIV exposure law and the HIV-related attitudes, beliefs, and sexual and seropositive status disclosure behaviors of persons living with HIV | Am J Public Health | Galletly, C. L.; Glasman, L. R.; Pinkerton, S. D.; Difranceisco, W. | yes | no | no | no |  | no |
| 17328657 | Outcomes of a multifaceted medication adherence intervention for HIV-positive patients | AIDS Patient Care STDS | Dieckhaus, K. D.; Odesina, V. | yes | no | no | no |  | no |
| 17563291 | Outreach program contacts: do they increase the likelihood of engagement and retention in HIV primary care for hard-to-reach patients? | AIDS Patient Care STDS | Cabral, H. J.; Tobias, C.; Rajabiun, S.; Sohler, N.; Cunningham, C.; Wong, M.; Cunningham, W. | yes | no | yes | no |  | no |
| 19911481 | Peer support and pager messaging to promote antiretroviral modifying therapy in Seattle: a randomized controlled trial | J Acquir Immune Defic Syndr | Simoni, J. M.; Huh, D.; Frick, P. A.; Pearson, C. R.; Andrasik, M. P.; Dunbar, P. J.; Hooton, T. M. | yes | no | no | no |  | no |
| 24093931 | Perceived stigma reductions following participation in mental health services integrated within community-based HIV primary care | AIDS Care | Farber, E. W.; Shahane, A. A.; Brown, J. L.; Campos, P. E. | yes | no | no | no |  | no |
| 20161771 | Perceptions of audio computer-assisted self-interviewing (ACASI) among women in an HIV-positive prevention program | PLoS One | Estes, L. J.; Lloyd, L. E.; Teti, M.; Raja, S.; Bowleg, L.; Allgood, K. L.; Glick, N. | yes | no | no | no |  | no |
| 21749845 | Personal meaning as a predictor of psychological well-being over time in individuals receiving HIV-related mental health services | Gen Hosp Psychiatry | Farber, E. W.; Woods, A.; Sherman, R. M.; Sharma, S. M.; Campos, P. E. | yes | no | no | no |  | no |
| 18457763 | Pilot testing HIV and intimate partner violence prevention modules among Spanish-speaking Latinas | J Assoc Nurses AIDS Care | Davila, Y. R.; Bonilla, E.; Gonzalez-Ramirez, D.; Grinslade, S.; Villarruel, A. M. | yes | no | no | no |  | no |
| 104483596. Language: | Pilot testing of an HIV medication adherence intervention in a public clinic in the Deep South | Journal of the American Academy of Nurse Practitioners | Konkle-Parker, Deborah J.; Erlen, Judith A.; Dubbert, Patricia M.; May, Warran | yes | no | no | no |  | no |
| 17879926 | Potential risks and benefits of HIV treatment simplification: a simulation model of a proposed clinical trial | Clin Infect Dis | Schackman, B. R.; Scott, C. A.; Sax, P. E.; Losina, E.; Wilkin, T. J.; McKinnon, J. E.; Swindells, S.; Weinstein, M. C.; Freedberg, K. A. | yes | no | no | no |  | no |
| 19234779 | Predicting response to cognitive-behavioral therapy in a sample of HIV-positive patients with chronic pain | J Behav Med | Cucciare, M. A.; Sorrell, J. T.; Trafton, J. A. | yes | no | no | no |  | no |
| 16639542 | Providers' HIV prevention discussions with HIV-seropositive injection drug users | AIDS Behav | Wilkinson, J. D.; Zhao, W.; Santibanez, S.; Arnsten, J.; Knowlton, A.; Gomez, C. A.; Metsch, L. R. | yes | yes | no | no |  | no |
| 18806878 | Quality of care for HIV infection provided by Ryan White Program-supported versus Non-Ryan White Program-supported facilities | PLoS One | Sullivan, P. S.; Denniston, M.; Mokotoff, E.; Buskin, S.; Broyles, S.; McNaghten, A. D. | yes | no | no | no |  | no |
| 23337369 | Randomized clinical trial of HIV treatment adherence counseling interventions for people living with HIV and limited health literacy | J Acquir Immune Defic Syndr | Kalichman, S. C.; Cherry, C.; Kalichman, M. O.; Amaral, C.; White, D.; Grebler, T.; Eaton, L. A.; Cruess, D.; Detorio, M. A.; Caliendo, A. M.; Schinazi, R. F. | yes | yes | no | no |  | no |
| 18626764 | Randomized controlled trial of a cognitive-behavioral intervention for HIV-positive persons: an investigation of treatment effects on psychosocial adjustment | AIDS Behav | Carrico, A. W.; Chesney, M. A.; Johnson, M. O.; Morin, S. F.; Neilands, T. B.; Remien, R. H.; Rotheram-Borus, M. J.; Lennie Wong, F. | yes | no | no | no |  | no |
| 19949848 | Randomized trial of the effects of housing assistance on the health and risk behaviors of homeless and unstably housed people living with HIV | AIDS Behav | Wolitski, R. J.; Kidder, D. P.; Pals, S. L.; Royal, S.; Aidala, A.; Stall, R.; Holtgrave, D. R.; Harre, D.; Courtenay-Quirk, C. | yes | no | no | no |  | no |
| 19505589 | Rationale, design, and sample characteristics of a randomized controlled trial of directly observed antiretroviral therapy delivered in methadone clinics | Contemp Clin Trials | Berg, K. M.; Mouriz, J.; Li, X.; Duggan, E.; Goldberg, U.; Arnsten, J. H. | yes | yes | yes | no |  | no |
| 23432593 | Reducing heavy drinking in HIV primary care: a randomized trial of brief intervention, with and without technological enhancement | Addiction | Hasin, D. S.; Aharonovich, E.; O'Leary, A.; Greenstein, E.; Pavlicova, M.; Arunajadai, S.; Waxman, R.; Wainberg, M.; Helzer, J.; Johnston, B. | yes | no | no | no |  | no |
| 18799777 | Reducing risky sexual behavior and substance use among currently and formerly homeless adults living with HIV | Am J Public Health | Rotheram-Borus, M. J.; Desmond, K.; Comulada, W. S.; Arnold, E. M.; Johnson, M. | yes | no | no | no |  | no |
| 23025248 | Reductions in traumatic stress following a coping intervention were mediated by decreases in avoidant coping for people living with HIV/AIDS and childhood sexual abuse | J Consult Clin Psychol | Sikkema, K. J.; Ranby, K. W.; Meade, C. S.; Hansen, N. B.; Wilson, P. A.; Kochman, A. | yes | no | no | no |  | no |
| 17931064 | Relaxation response with acupuncture trial in patients with HIV: feasibility and participant experiences | J Altern Complement Med | Chang, B. H.; Boehmer, U.; Zhao, Y.; Sommers, E. | yes | no | no | no |  | no |
| 23636311 | Reliability and validity of a treatment fidelity assessment for motivational interviewing targeting sexual risk behaviors in people living with HIV/AIDS | J Clin Psychol Med Settings | Seng, E. K.; Lovejoy, T. I. | yes | no | no | no |  | no |
| 18089983 | Results from a randomized controlled trial of a peer-mentoring intervention to reduce HIV transmission and increase access to care and adherence to HIV medications among HIV-seropositive injection drug users | J Acquir Immune Defic Syndr | Purcell, D. W.; Latka, M. H.; Metsch, L. R.; Latkin, C. A.; Gomez, C. A.; Mizuno, Y.; Arnsten, J. H.; Wilkinson, J. D.; Knight, K. R.; Knowlton, A. R.; Santibanez, S.; Tobin, K. E.; Rose, C. D.; Valverde, E. E.; Gourevitch, M. N.; Eldred, L.; Borkowf, C. B. | yes | no | no | no |  | no |
| 25139373 | Results from a secondary data analysis regarding satisfaction with health care among African American women living with HIV/AIDS | J Obstet Gynecol Neonatal Nurs | Baker, J. L.; Rodgers, C. R.; Davis, Z. M.; Gracely, E.; Bowleg, L. | yes | yes | no | no |  | no |
| 18794720 | Sleep disturbances in women with HIV or AIDS: efficacy of a tailored sleep promotion intervention | Nurs Res | Hudson, A. L.; Portillo, C. J.; Lee, K. A. | yes | no | no | no |  | no |
| 105295242. Language: | Social support and adherence: differences among clients in an AIDS day health care program | JANAC: Journal of the Association of Nurses in AIDS Care | Gardenier, D.; Andrews, C. M.; Thomas, D. C.; Bookhardt-Murray, L. J.; Fitzpatrick, J. J. | yes | yes | no | no |  | no |
| 109830186. Language: | Social Work and the HIV Care Continuum: Assisting HIV Patients Diagnosed in an Emergency Department | Social Work | Edmonds, Amy; Moore, Eric; Valdez, Andre; Tomlinson, Cheri | yes | yes | yes | no |  | no |
| 24093810 | Stages of change for adherence to antiretroviral medications | AIDS Patient Care STDS | Genberg, B. L.; Lee, Y.; Rogers, W. H.; Willey, C.; Wilson, I. B. | yes | no | no | no |  | no |
| 105772863. Language: | Stress management interventions for HIV+ adults: a meta-analysis of randomized controlled trials, 1989 to 2006 | Health Psychology | Scott-Sheldon, L. A.; Kalichman, S. C.; Carey, M. P.; Fielder, R. L. | yes | no | no | no |  | no |
| 18329222 | Substance abuse treatment in human immunodeficiency virus: the role of patient-provider discussions | J Subst Abuse Treat | Korthuis, P. T.; Josephs, J. S.; Fleishman, J. A.; Hellinger, J.; Himelhoch, S.; Chander, G.; Morse, E. B.; Gebo, K. A. | yes | no | no | no |  | no |
| 17712763 | Superiority of directly administered antiretroviral therapy over self-administered therapy among HIV-infected drug users: a prospective, randomized, controlled trial | Clin Infect Dis | Altice, F. L.; Maru, D. S.; Bruce, R. D.; Springer, S. A.; Friedland, G. H. | yes | no | no | no |  | no |
| 23474642 | Supportive-expressive and coping group teletherapies for HIV-infected older adults: a randomized clinical trial | AIDS Behav | Heckman, T. G.; Heckman, B. D.; Anderson, T.; Lovejoy, T. I.; Mohr, D.; Sutton, M.; Bianco, J. A.; Gau, J. T. | yes | no | no | no |  | no |
| 18728987 | Sustained antiretroviral treatment adherence in survivors of the pre-HAART era: attitudes and beliefs | AIDS Care | Fumaz, C. R.; Munoz-Moreno, J. A.; Molto, J.; Ferrer, M. J.; Lopez-Blazquez, R.; Negredo, E.; Paredes, R.; Gomez, G.; Clotet, B. | yes | yes | no | no |  | no |
| 17091022 | Sustained benefit from a long-term antiretroviral adherence intervention. Results of a large randomized clinical trial | J Acquir Immune Defic Syndr | Mannheimer, S. B.; Morse, E.; Matts, J. P.; Andrews, L.; Child, C.; Schmetter, B.; Friedland, G. H. | yes | no | no | no |  | no |
| 105279406. Language: | Symptoms of psychological distress: a comparison of rural and urban individuals enrolled in HIV-related mental health care | AIDS Patient Care & STDs | Basta, T. B.; Shacham, E.; Reece, M. | yes | yes | no | no |  | no |
| 103896180. Language: | Systematic Review of Couple-Based HIV Intervention and Prevention Studies: Advantages, Gaps, and Future Directions | AIDS & Behavior | Jiwatram-NegrÃ³n, Tina; El-Bassel, Nabila | yes | yes | no | no |  | no |
| 19576548 | Telephone nurse counseling improves HIV medication adherence: an effectiveness study | J Assoc Nurses AIDS Care | Cook, P. F.; McCabe, M. M.; Emiliozzi, S.; Pointer, L. | yes | no | no | no |  | no |
| 21809048 | Telephone-administered motivational interviewing reduces risky sexual behavior in HIV-positive late middle-age and older adults: a pilot randomized controlled trial | AIDS Behav | Lovejoy, T. I.; Heckman, T. G.; Suhr, J. A.; Anderson, T.; Heckman, B. D.; France, C. R. | yes | no | no | no |  | no |
| 20118830 | Telephone-administered motivational interviewing to reduce risky sexual behavior in HIV-infected rural persons: a pilot randomized clinical trial | Sex Transm Dis | Cosio, D.; Heckman, T. G.; Anderson, T.; Heckman, B. D.; Garske, J.; McCarthy, J. | yes | no | no | no |  | no |
| 22956397 | Telephone-delivered motivational interviewing targeting sexual risk behavior reduces depression, anxiety, and stress in HIV-positive older adults | Ann Behav Med | Lovejoy, T. I. | yes | no | no | no |  | no |
| 18678684 | Telephone-delivered, interpersonal psychotherapy for HIV-infected rural persons with depression: a pilot trial | Psychiatr Serv | Ransom, D.; Heckman, T. G.; Anderson, T.; Garske, J.; Holroyd, K.; Basta, T. | yes | no | no | no |  | no |
| 23760634 | Test of a web-based program to improve adherence to HIV medications | AIDS Behav | Hersch, R. K.; Cook, R. F.; Billings, D. W.; Kaplan, S.; Murray, D.; Safren, S.; Goforth, J.; Spencer, J. | yes | no | no | no |  | no |
| 17983336 | The combined effect of relaxation response and acupuncture on quality of life in patients with HIV: a pilot study | J Altern Complement Med | Chang, B. H.; Boehmer, U.; Zhao, Y.; Sommers, E. | yes | no | no | no |  | no |
| 22955429 | The effect of a 'universal antiretroviral therapy' recommendation on HIV RNA levels among HIV-infected patients entering care with a CD4 count greater than 500/muL in a public health setting | Clin Infect Dis | Geng, E. H.; Hare, C. B.; Kahn, J. O.; Jain, V.; Van Nunnery, T.; Christopoulos, K. A.; Deeks, S. G.; Gandhi, M.; Havlir, D. V. | yes | no | no | no |  | no |
| 18260805 | The feasibility of modified directly observed therapy for HIV-seropositive African American substance users | AIDS Patient Care STDS | Ma, M.; Brown, B. R.; Coleman, M.; Kibler, J. L.; Loewenthal, H.; Mitty, J. A. | yes | no | no | no |  | no |
| 19372524 | The health impact of supportive housing for HIV-positive homeless patients: a randomized controlled trial | Am J Public Health | Buchanan, D.; Kee, R.; Sadowski, L. S.; Garcia, D. | yes | no | no | no |  | no |
| 17446833 | The impact of a quality improvement program on systems, processes, and structures in medical clinics | Med Care | McInnes, D. K.; Landon, B. E.; Wilson, I. B.; Hirschhorn, L. R.; Marsden, P. V.; Malitz, F.; Barini-Garcia, M.; Cleary, P. D. | yes | no | no | no |  | no |
| 23631790 | The impact of social context on self-management in women living with HIV | Soc Sci Med | Webel, A. R.; Cuca, Y.; Okonsky, J. G.; Asher, A. K.; Kaihura, A.; Salata, R. A. | yes | no | no | no |  | no |
| 23208648 | The impact of substance use, sexual trauma, and intimate partner violence on sexual risk intervention outcomes in couples: a randomized trial | Ann Behav Med | Jones, D. L.; Kashy, D.; Villar-Loubet, O. M.; Cook, R.; Weiss, S. M. | yes | yes | no | no |  | no |
| 24325326 | The role of at-risk alcohol/drug use and treatment in appointment attendance and virologic suppression among HIV(+) African Americans | AIDS Res Hum Retroviruses | Howe, C. J.; Cole, S. R.; Napravnik, S.; Kaufman, J. S.; Adimora, A. A.; Elston, B.; Eron, J. J., Jr.; Mugavero, M. J. | yes | no | no | no |  | no |
| 103916111. Language: | The Validity of Self-Reported Medication Adherence as an Outcome in Clinical Trials of Adherence-Promotion Interventions: Findings from the MACH14 Study | AIDS & Behavior | Simoni, Jane; Huh, David; Wang, Yan; Wilson, Ira; Reynolds, Nancy; Remien, Robert; Goggin, Kathy; Gross, Robert; Rosen, Marc; Schneiderman, Neil; Arnsten, Julia; Golin, Carol; Erlen, Judith; Bangsberg, David; Liu, Honghu | yes | no | no | no |  | no |
| 16019998 | Transforming the meaning of HIV/AIDS in recovery from substance use: a qualitative study of HIV-positive women in New York | Health Care Women Int | Unger, H. V.; Collins, P. Y. | yes | yes | no | no |  | no |
| 106496415. Language: | Transforming the meaning of HIV/AIDS in recovery from substance use: a qualitative study of HIV-positive women in New York | Health Care for Women International | Von Unger, H.; Collins, P. Y. | yes | yes | no | no |  | no |
| 23128979 | Transitional care coordination in New York City jails: facilitating linkages to care for people with HIV returning home from Rikers Island | AIDS Behav | Jordan, A. O.; Cohen, L. R.; Harriman, G.; Teixeira, P. A.; Cruzado-Quinones, J.; Venters, H. | yes | no | no | no |  | no |
| 23725449 | Use of web-based shared medical records among patients with HIV | Am J Manag Care | Ralston, J. D.; Silverberg, M. J.; Grothaus, L.; Leyden, W. A.; Ross, T.; Stewart, C.; Carzasty, S.; Horberg, M.; Catz, S. L. | yes | no | yes | no |  | no |
| 23515640 | Using community health workers to improve clinical outcomes among people living with HIV: a randomized controlled trial | AIDS Behav | Kenya, S.; Jones, J.; Arheart, K.; Kobetz, E.; Chida, N.; Baer, S.; Powell, A.; Symes, S.; Hunte, T.; Monroe, A.; Carrasquillo, O. | yes | no | yes | no |  | no |
| 17113345 | Using digital videos displayed on personal digital assistants (PDAs) to enhance patient education in clinical settings | Int J Med Inform | Brock, T. P.; Smith, S. R. | yes | no | no | no |  | no |
| 23651105 | Utility of an interactive voice response system to assess antiretroviral pharmacotherapy adherence among substance users living with HIV/AIDS in the rural South | AIDS Patient Care STDS | Tucker, J. A.; Simpson, C. A.; Huang, J.; Roth, D. L.; Stewart, K. E. | yes | no | no | no |  | no |
| 20059570 | Yoga lifestyle intervention reduces blood pressure in HIV-infected adults with cardiovascular disease risk factors | HIV Med | Cade, W. T.; Reeds, D. N.; Mondy, K. E.; Overton, E. T.; Grassino, J.; Tucker, S.; Bopp, C.; Laciny, E.; Hubert, S.; Lassa-Claxton, S.; Yarasheski, K. E. | yes | no | no | no |  | no |
| 15751766 | A randomized controlled trial to enhance antiretroviral therapy adherence in patients with a history of alcohol problems | Antivir Ther | Samet, J. H.; Horton, N. J.; Meli, S.; Dukes, K.; Tripps, T.; Sullivan, L.; Freedberg, K. A. | yes | no | no | no |  | no |
| 22010810 | Girlfriends: evaluation of an HIV-risk reduction intervention for adult transgender women | AIDS Educ Prev | Taylor, R. D.; Bimbi, D. S.; Joseph, H. A.; Margolis, A. D.; Parsons, J. T. | yes | no | no | no |  | no |
| 16006211 | HIV vaccine acceptability among women at risk: perceived barriers and facilitators to future HIV vaccine uptake | AIDS Educ Prev | Rudy, E. T.; Newman, P. A.; Duan, N.; Kelly, E. M.; Roberts, K. J.; Seiden, D. S. | yes | yes | no | no |  | no |
| 20178024 | Preliminary outcomes of a model program for increasing treatment access for African American women who use crack cocaine and are at risk for contracting HIV | J Evid Based Soc Work | Okpaku, S.; Macmaster, S. A.; Dennie, S.; Tolliver, D.; Cooper, R. L.; Rasch, R. F. | yes | no | no | no |  | no |
| 105140793. Language: | Relationships between stigma, social support, and depression in HIV-infected African American women living in the rural southeastern United States | JANAC: Journal of the Association of Nurses in AIDS Care | Vyavaharkar, M.; Moneyham, L.; Corwin, S.; Saunders, R.; Annang, L.; Tavakoli, A. | yes | yes | no | no |  | no |
| 17005999 | Workforce reentry for people with HIV/AIDS: intervention effects and predictors of success | Work | Martin, D. J.; Arns, P. G.; Batterham, P. J.; Afifi, A. A.; Steckart, M. J. | yes | no | no | no |  | no |
| 25512179 | Couple-based HIV counseling and testing: a risk reduction intervention for US drug-involved women and their primary male partners | Prev Sci | McMahon, J. M. and Pouget, E. R. and Tortu, S. | yes | no | no | no |  | no |
| 20640958 | Improving adherence and clinical outcomes through an HIV pharmacist's interventions | AIDS Care | Ma, A.; Chen, D. M.; Chau, F. M.; Saberi, P. | yes | no | no | no |  | no |
| 21347897 | Social services utilization and need among a community sample of persons living with HIV in the rural south | AIDS Care | Stewart, K. E.; Phillips, M. M.; Walker, J. F.; Harvey, S. A.; Porter, A. | yes | no | no | no |  | no |
| 26103592 | Social Support, Stigma and Disclosure: Examining the Relationship with HIV Medication Adherence among Ryan White Program Clients in the Mid-South USA | Int J Environ Res Public Health | Pichon, L. C. | yes | yes | no | no |  | no |
| 21939408 | Time to first annual HIV care visit and associated factors for patients in care for HIV infection in 10 US cities | AIDS Care | Sullivan, P. S.; Juhasz, M.; McNaghten, A. D.; Frankel, M.; Bozzette, S.; Shapiro, M. | yes | no | no | no |  | no |
| 26322677 | Service Delivery and Patient Outcomes in Ryan White HIV/AIDS Program-Funded and -Nonfunded Health Care Facilities in the United States | JAMA Intern Med | Huff, A. and Chumbler, N. and Cherry, C. O. | yes | no | yes | no |  | no |
| 22828593 | A low-effort, clinic-wide intervention improves attendance for HIV primary care | Clin Infect Dis | Gardner, L. I.; Marks, G.; Craw, J. A.; Wilson, T. E.; Drainoni, M. L.; Moore, R. D.; Mugavero, M. J.; Rodriguez, A. E.; Bradley-Springer, L. A.; Holman, S.; Keruly, J. C.; Sullivan, M.; Skolnik, P. R.; Malitz, F.; Metsch, L. R.; Raper, J. L.; Giordano, T. P. | yes | no | no | no |  | no |
| 22984780 | Feasibility, acceptability, and preliminary efficacy of the unity workshop: an internalized stigma reduction intervention for African American women living with HIV | AIDS Patient Care STDS | Rao, D.; Desmond, M.; Andrasik, M.; Rasberry, T.; Lambert, N.; Cohn, S. E.; Simoni, J. | yes | yes | no | no |  | no |
| 20025513 | Using the Internet to provide care for persons living with HIV | AIDS Patient Care STDS | Horvath, K. J.; Courtenay-Quirk, C.; Harwood, E.; Fisher, H.; Kachur, R.; McFarlane, M.; O'Leary, A.; Rosser, B. R. | yes | no | no | no |  | no |
| 104152050. Language: | WiLLOW: Reaching HIV-Positive African-American Women Through a Computer-Delivered Intervention | AIDS & Behavior | Klein, Charles; Lomonaco, Carmela; Pavlescak, Rik; Card, Josefina | yes | no | no | no |  | no |
| 16041896 | Improvements in health-related quality of life following a group intervention for coping with AIDS-bereavement among HIV-infected men and women | Qual Life Res | Sikkema, K. J.; Hansen, N. B.; Meade, C. S.; Kochman, A.; Lee, R. S. | yes | no | no | no |  | no |
| 25043931 | Real-time medication adherence monitoring intervention: test of concept in people living with HIV infection | J Assoc Nurses AIDS Care | Pellowski, J. A.; Kalichman, S. C.; White, D.; Amaral, C. M.; Hoyt, G.; Kalichman, M. O. | yes | no | no | no |  | no |
| 23625127 | Spirituality and optimism: a holistic approach to component-based, self-management treatment for HIV | J Relig Health | Brown, J.; Hanson, J. E.; Schmotzer, B.; Webel, A. R. | yes | no | no | no |  | no |
| 18191769 | Improving women's adjustment to HIV infection: results of the Positive Life Skills Workshop Project | J Assoc Nurses AIDS Care | Bova, C.; Burwick, T. N.; Quinones, M. | yes | no | no | no |  | no |
| 25331820 | Emergency department use among HIV-infected released jail detainees | J Urban Health | Boyd, A. T. | yes | yes | no | no |  | no |
| 20024721 | Trends in hospitalizations with psychiatric diagnoses among HIV-infected women in the USA, 1994-2004 | AIDS Care | Bansil, P.; Jamieson, D. J.; Posner, S. F.; Kourtis, A. P. | no | Yes | no | no |  | no |
| 17570298 | Retaining women in HIV medical care | J Assoc Nurses AIDS Care | Andersen, M.; Hockman, E.; Smereck, G.; Tinsley, J.; Milfort, D.; Wilcox, R.; Smith, T.; Connelly, C.; Adams, L.; Thomas, R. | yes | no | yes | no |  | no |
| 17971712 | The influence of psychosocial characteristics and race/ethnicity on the use, duration, and success of antiretroviral therapy | J Acquir Immune Defic Syndr | Pence, B. W.; Ostermann, J.; Kumar, V.; Whetten, K.; Thielman, N.; Mugavero, M. J. | no | yes | yes | no |  | no |
